# Deficit Irrigation Strategies in Almond Orchards: A Review of the Effects on Yield, Physiology, Nut Quality, Economics, and Remote Sensing

**DOI:** 10.3390/plants15213253

**Published:** 2026-10-22

**Authors:** Annalisa Tarantino, Laura Frabboni, Antonio Stasi, Grazia Disciglio

**Affiliations:** Department of Agriculture, Food, Natural Resources and Engineering (DAFNE), University of Foggia, Via Napoli 25, 71122 Foggia, Italy; laura.frabboni@unifg.it (L.F.); antonio.stasi@unifg.it (A.S.); grazia.disciglio@unifg.it (G.D.)

**Keywords:** regulated deficit irrigation (RDI), sustained deficit irrigation (SDI), partial root-zone drying (PRD), low-frequency deficit irrigation (LFDI), water stress, arid and semi-arid regions, water productivity, yield and kernel quality, vegetative growth, remote sensing

## Abstract

Sustainable water use is a major challenge in irrigated agriculture, particularly in arid and semi-arid regions where water availability strongly influences crop productivity and quality. Almond (*Prunus dulcis* (Mill.) D.A. Webb) is an important nut crop cultivated extensively in such environments, making efficient irrigation management essential for sustainable production. A total of 63 studies were included in the review, comprising 62 field studies and one pot experiment, some of which incorporated modelling, remote sensing, physiological monitoring, or sensor-based irrigation-management approaches, to evaluate deficit irrigation (DI) in almond, with particular emphasis on regulated deficit irrigation (RDI), sustained deficit irrigation (SDI), low-frequency deficit irrigation (LFDI), and partial root-zone drying (PRD). The review examines how irrigation strategy, deficit intensity, timing and duration of water restriction, cultivar, tree age, and environmental conditions affect irrigation water savings, yield, kernel quality, water productivity, water-use efficiency, and physiological responses. In addition, the economic implications of DI and recent applications of remote sensing (RS) for detecting water stress and supporting irrigation scheduling are evaluated. Overall, the evidence indicates that DI can substantially reduce irrigation water use while maintaining acceptable yields and, under specific conditions, improving kernel quality. However, responses vary considerably among studies, reflecting interactions among deficit characteristics, environmental conditions, cultivar, and tree age and limiting the development of universally applicable management recommendations. The review further indicates that integrating DI with RS-based monitoring has potential to support site-specific and adaptive irrigation management by improving water allocation, detecting emerging water stress, and enhancing water-use efficiency. Such integrated approaches may become increasingly important in regions experiencing greater drought frequency and competition for water resources. Future research should prioritize multi-environment and multi-cultivar validation of integrated DI–RS approaches, the development of practical decision-support tools and economic thresholds, and the assessment of their transferability from experimental conditions to commercial orchards. Combining physiological indicators, RS technologies, and crop-specific irrigation strategies could contribute to more resilient and sustainable almond production under increasing water scarcity and climate variability.

## 1. Introduction

Water availability for agriculture is becoming increasingly limited, particularly in arid and semi-arid regions. Climate change is expected to exacerbate water scarcity through more frequent and severe droughts, altered precipitation patterns, increased evapotranspiration, and rising irrigation requirements [1]. Consequently, sustainable water management and innovative irrigation strategies are essential for improving agricultural resilience and securing future food production.

Almond (*Prunus dulcis* (Mill.) D.A. Webb) is an economically important nut crop widely cultivated in arid and semi-arid regions, including the Mediterranean basin, parts of the United States, Sub-Saharan Africa, and Australia [2]. Its widespread cultivation is largely attributed to its adaptation to mild winters and hot, dry summers, as well as its relatively low chilling requirement and early flowering habit [3]. In recent years, almonds have gained increasing economic and nutritional importance because of their high content of valuable nutrients and bioactive compounds, including fatty acids, lipids, proteins, amino acids, carbohydrates, vitamins, minerals, and secondary metabolites [4].

Worldwide, almond production is concentrated in several major producing regions. However, annual production varies considerably among countries and from year to year due to differences in climatic conditions, water availability, cultivated area, yield, and production systems.

According to FAOSTAT [5], the United States of America was the leading almond-producing country in 2024, with approximately 2,000,340 tonnes of almonds in shell. California accounts for the vast majority of U.S. almond production. According to USDA/NASS [6], California produced approximately 2.725 billion pounds of shelled almonds in 2024, corresponding to approximately 1.24 million tonnes of almond kernels.

Although almonds were traditionally cultivated under rainfed conditions, modern production has increasingly shifted towards irrigated systems because water deficits can substantially reduce yield and kernel weight, thereby affecting orchard profitability [7]. Almond × peach hybrid rootstocks, such as GF-677 and the G × N series, are widely used because of their high vigour, adaptability to calcareous and relatively dry soils, and good performance under Mediterranean conditions [8,9]. In contrast, the suitability of size-controlling rootstocks, including the Rootpac^®^ series, for drought-prone environments remains less well established, as relatively few studies have evaluated their physiological and agronomic responses to water deficit [10,11,12]. Despite the high genetic diversity of almond varieties in the Mediterranean basin [13], commercial production is currently dominated by a limited number of cultivars [14], particularly in newly established intensive orchards [15]. These systems, including medium-to high-density (MHD; approximately 300–1000 trees ha^−1^) and super-high-density (SHD; more than 2000 trees ha^−1^) plantings, are designed to improve mechanization, sustainability, and production efficiency [16]. The adoption of intensive production systems can also promote earlier bearing. Conventional almond orchards generally begin producing commercially around the third year after planting and may reach full production after approximately six years, whereas high-density systems can achieve earlier production and accelerate the attainment of maximum productivity [17].

As previously mentioned, the global almond industry is characterized by the commercial cultivation of a relatively limited number of cultivars, although cultivar choice varies considerably depending on regional agro-climatic conditions, production systems, and breeding programs. In California, commercial production is dominated by cultivars such as ‘Nonpareil’, ‘Carmel’, ‘Mission’, ‘Texas’, ‘Ne Plus Ultra’, and ‘Ruby’, whereas in Australia, the main commercial cultivars include ‘Nonpareil’, ‘Carmel’, ‘Price’, and ‘Monterey’. In Mediterranean countries, including Spain and Italy, cultivar diversity is comparatively greater, with cultivars such as ‘Soleta’, ‘Ferragnès’, ‘Lauranne^®^ Avijor’, ‘Penta’, and ‘Guara-Tuono’ being widely cultivated. Similarly, ‘Ferragnès’ and ‘Marcona’ are important cultivars in Morocco, while ‘Ferragnès’, ‘Ferraduel’, and ‘Tuono’ are widely grown in Syria and Turkey. In Iran, ‘Badamak’ remains an important local cultivar [15,17,18,19].

Almond is generally considered drought-tolerant rather than drought-resistant [20], as it can withstand moderate and seasonal water deficits through physiological mechanisms that help maintain plant function under water-limited conditions. However, prolonged or severe water deficits can substantially reduce productivity, while adequate water supply is required to maximize yield and kernel size [7]. Conventional irrigation practices generally aim to satisfy most or all of the crop evapotranspiration requirements (ETc), thereby minimizing water stress; however, full irrigation may result in inefficient water use in regions where water availability is limited. In Mediterranean environments, where water scarcity often constrains the application of full irrigation, improving water-use efficiency (WUE) has therefore become a major priority. In this context, deficit irrigation (DI), defined as the deliberate application of water below full crop water requirements, has emerged as a strategy for reducing irrigation water use while maintaining acceptable levels of yield and fruit quality [21]. DI has also been shown to modify certain almond quality attributes, including some biochemical and nutritional characteristics, although responses depend on the irrigation strategy and the quality parameter considered [22]. More broadly, regulated deficit irrigation (RDI) is recognized as an effective water-saving strategy for horticultural crops, particularly fruit and vegetable crops, when deficit periods are appropriately timed and controlled [23,24]. Accordingly, improving irrigation efficiency and water productivity (WP) is essential for achieving sustainable almond production under increasing water scarcity [25]. In parallel, advances in plant water status (PWS) monitoring, based on direct physiological measurements and remote-sensing (RS) techniques, are providing increasingly valuable tools for optimizing irrigation scheduling and improving the implementation of deficit-irrigation strategies. For almond, thermal remote sensing and canopy temperature measurements have demonstrated potential for estimating plant water status and identifying water stress under deficit irrigation.

To clarify the position and novelty of the present synthesis within the existing literature, it is important to consider how its scope compares with previous reviews addressing almond irrigation, water management, crop physiology, nut quality, and remote-sensing applications.

Several studies and reviews have examined individual components of these topics, but their objectives, geographical coverage, and thematic scope differ from the integrated perspective adopted in the present review. Quantitative assessments of irrigation regimes and yield responses have been reported for Mediterranean almond production systems [26], providing important evidence on the relationship between irrigation supply and productivity. However, this work primarily addresses irrigation treatments and yield responses and does not comprehensively integrate physiological indicators, nut quality, and precision-irrigation approaches. Reviews addressing almond nut quality and sensory assessment have focused mainly on nutritional, physicochemical, and sensory characteristics [27], with less emphasis on the interaction between irrigation management and these quality attributes. More broadly, the physiological and agronomic responses of crops to regulated deficit irrigation (RDI), including mechanisms underlying improved water-use efficiency (WUE) and adaptation to water deficit, have been comprehensively reviewed [28]. Although highly relevant for understanding plant responses to water limitation, this review adopts a broader crop perspective rather than focusing specifically on the integrated irrigation-management challenges of almond production. Sustainable irrigation intensification and the use of deficit irrigation (DI) strategies in almond and pistachio production have also been reviewed, with emphasis on improving WUE while maintaining yield under water-limited conditions [29]. However, the scope encompasses more than almond and does not comprehensively connect irrigation strategies with nut quality, physiological indicators, and remote-sensing-based irrigation management. A more recent review specifically examined DI strategies for almond production in California, with particular emphasis on irrigation management and the effects of DI timing and severity on almond production [30]. This represents an important almond-specific contribution; however, its focus is primarily on DI management and production responses in a specific production context, rather than on the broader integration of physiology, nut quality, water productivity, and precision irrigation. The physiological basis of almond irrigation management has also been reviewed, with emphasis on water potential, stomatal conductance, leaf temperature, phenological stage, and cultivar-specific responses as indicators for irrigation scheduling [31]. This work provides an important physiological basis for irrigation decision-making, but does not comprehensively integrate these indicators with DI strategies, yield, nut quality, and remote-sensing technologies. Similarly, remote-sensing approaches for irrigation monitoring have been reviewed broadly across horticultural crops, including applications for crop water status, evapotranspiration, canopy temperature, and precision irrigation [32]. A systematic review specifically addressing remote sensing in almond orchards has further summarized satellite, UAV, and proximal sensing technologies, identifying water management as one of the principal application areas [33]. These reviews primarily focus on sensing technologies and their applications rather than on their integration with irrigation strategy, physiological responses, productivity, and water-use outcomes.

To further clarify the scope of previous reviews and the specific contribution of the present synthesis, Table 1 summarizes the main focus, scope, and thematic coverage of closely related reviews, together with the principal aspects that remain insufficiently integrated in the existing literature. Previous reviews have addressed important individual aspects of almond irrigation, including irrigation regimes, deficit irrigation, plant physiological responses, nut quality, and remote sensing. However, these dimensions have generally been considered separately, with limited integration of irrigation management, plant physiology, nut quality, water productivity, and precision- and remote-sensing approaches within a unified framework. This comparison highlights the complementary nature of previous contributions and positions the present review within the broader literature by emphasizing its integrative approach to almond irrigation management, physiological responses, nut quality, water productivity, and plant water-status monitoring.

## 2. Review Methodology

This review synthesizes current knowledge derived from field studies on deficit irrigation (DI) strategies, including regulated deficit irrigation (RDI), sustained deficit irrigation (SDI), low-frequency deficit irrigation (LFDI), and partial root-zone drying (PRD), as well as their effects on yield quantity and quality, irrigation water savings, plant physiological responses, and cultivar adaptation. The review also examines recent advances in the application of remote sensing (RS) technologies for detecting water stress and improving irrigation management in almond orchards. In addition, a comprehensive bibliography is provided as a resource for researchers and practitioners working on almond irrigation and remote sensing applications.

### 2.1. Review Design and Scope

This article presents a structured narrative review of peer-reviewed literature on deficit irrigation (DI) in almond (*Prunus dulcis* orchards. The review focused on regulated deficit irrigation (RDI), sustained deficit irrigation (SDI), and partial root-zone drying (PRD), with emphasis on irrigation timing and strategy, physiological and biochemical responses, growth, yield and productivity, water-use efficiency, kernel composition and quality, cultivar and tree-age effects, multi-season responses, economic aspects, and remote sensing-based assessment of water stress. Relevant studies were identified through searches of Google Scholar and other scientific databases and publisher platforms using combinations of terms related to almond, water deficit, irrigation management, plant physiology, yield, kernel quality, water-use efficiency, economics, and remote sensing. Reference lists of relevant publications were also examined to identify additional studies. Eligible studies were peer-reviewed investigations involving almond trees and addressing one or more of the review objectives. Studies unrelated to almond or irrigation/water deficit, as well as duplicate and non-scientific publications, were excluded. Information from the selected literature was thematically organized and synthesized qualitatively according to irrigation strategy, phenological stage, physiological response, productivity, kernel quality, long-term effects, cultivar and tree age, economic performance, and remote sensing applications.

### 2.2. Study Selection

**Figure 1:**
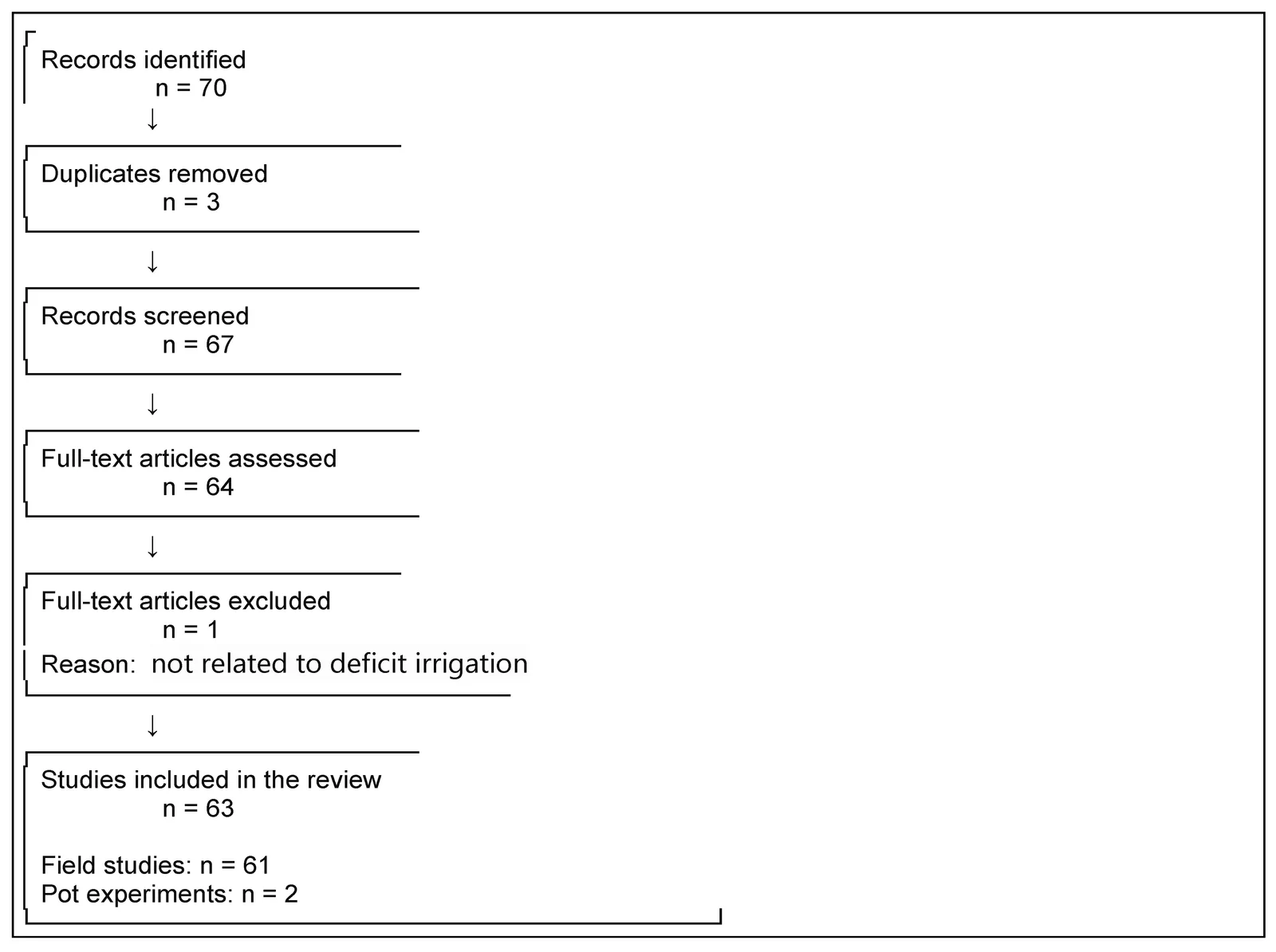
PRISMA-style flow diagram showing the identification, screening, eligibility assessment, and final inclusion of studies in the review.

The study selection process followed a PRISMA-style approach. A total of 70 records were identified through the literature search. After removal of 3 duplicate records, 67 records remained for title and abstract screening. Following screening, 64 articles were assessed in full text for eligibility. One full-text article was excluded because it did not meet the predefined eligibility criteria. The final evidence base comprised 63 studies, including 61 field studies conducted under orchard conditions and two pot-based experiments. The complete study identification, screening, eligibility assessment, and inclusion process is presented in Figure 1.

## 3. Water Requirements of Almond Trees

Water requirements of almond orchards vary considerably depending on climatic conditions, tree age, canopy development, planting density, soil characteristics, and irrigation management. Seasonal water requirements reported for mature almond orchards range from approximately 8500 m^3^ ha^−1^ in Spain to 12,000–13,000 m^3^ ha^−1^ in California, where the average fractional canopy cover commonly exceeds 80% [7,34,35,36]. In Sicily, irrigation requirements have been estimated at approximately 2500 m^3^ ha^−1^, with 1500–2000 m^3^ ha^−1^ required during the period from flowering to harvest [37].

Crop water requirements are commonly expressed in terms of crop evapotranspiration (ETc), which can be estimated from reference evapotranspiration (ET_0_) and the crop coefficient (Kc) according to the relationship: ETc = ET_0_ × Kc.

Reference evapotranspiration (ET_0_) is generally estimated using the standardized FAO Penman–Monteith equation [38]. The crop coefficient (Kc) varies according to phenological stage, canopy development, tree age, planting density, and orchard-management practices [39,40]. Reported Kc values differ substantially among almond orchards and should be interpreted in relation to the specific climatic, phenological, and management conditions under which they were determined [40,41,42,43,44,45,46,47,48,49,50].

**Figure 2:**
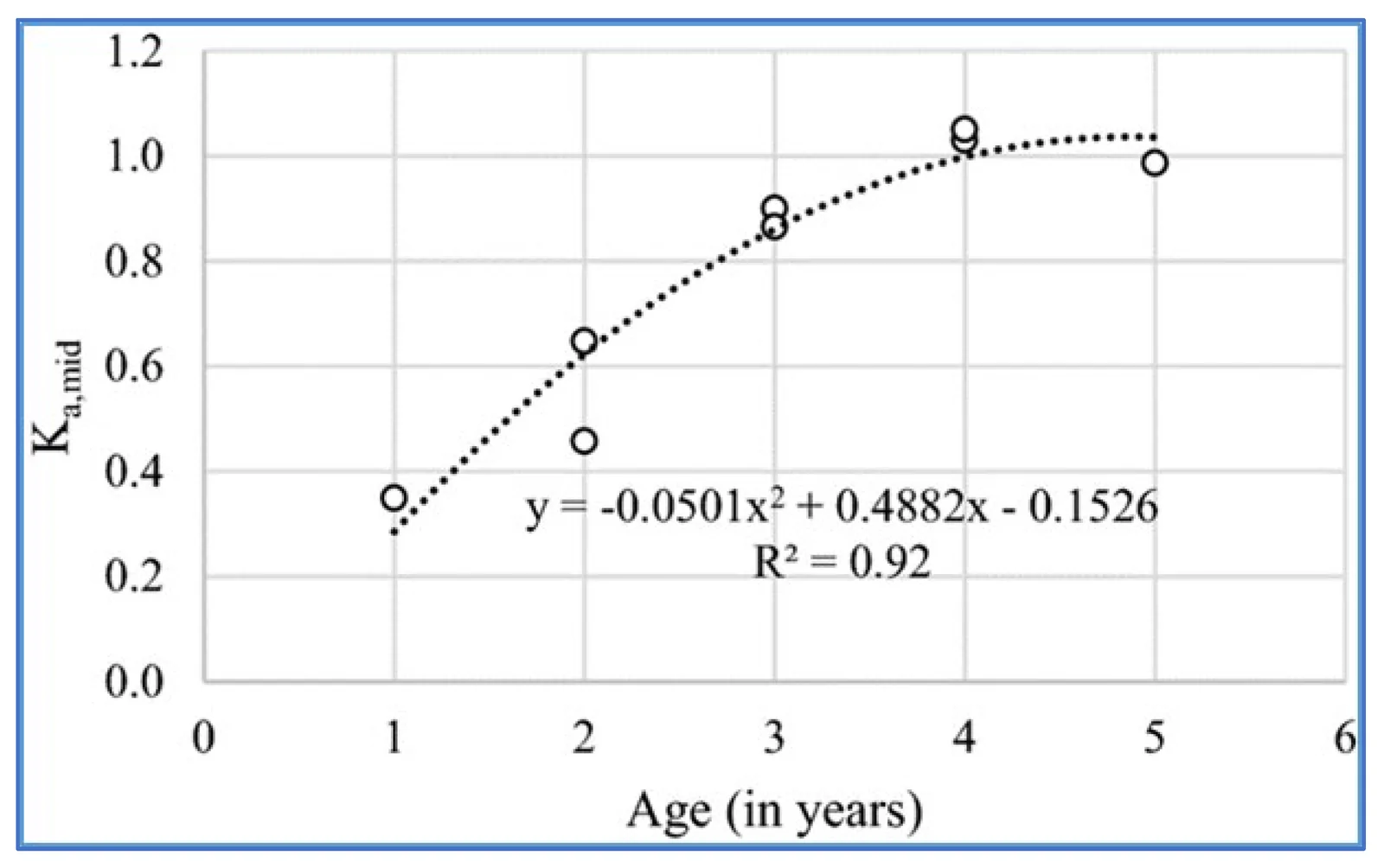
Mid-season actual crop coefficients (Ka mid,) in young almond orchards aged 1–5 years (source: Drechsler et al., 2022a) [47].

Tree age and canopy development are particularly important determinants of Kc. In young almond orchards, Kc values remain relatively low, at approximately 0.35, because of limited canopy cover and the resulting small transpiring surface. As orchards develop, Kc increases with tree age and canopy expansion. For developing almond orchards, reported mid-season crop coefficients (Kc, mid) range from approximately 0.35 in the youngest orchards to 0.55, 0.88, 1.04, and 0.99 in orchards aged 1–5 years, respectively [47] (Figure 2). These values illustrate the progressive increase in crop water use associated with canopy development during orchard establishment.

**Figure 3:**
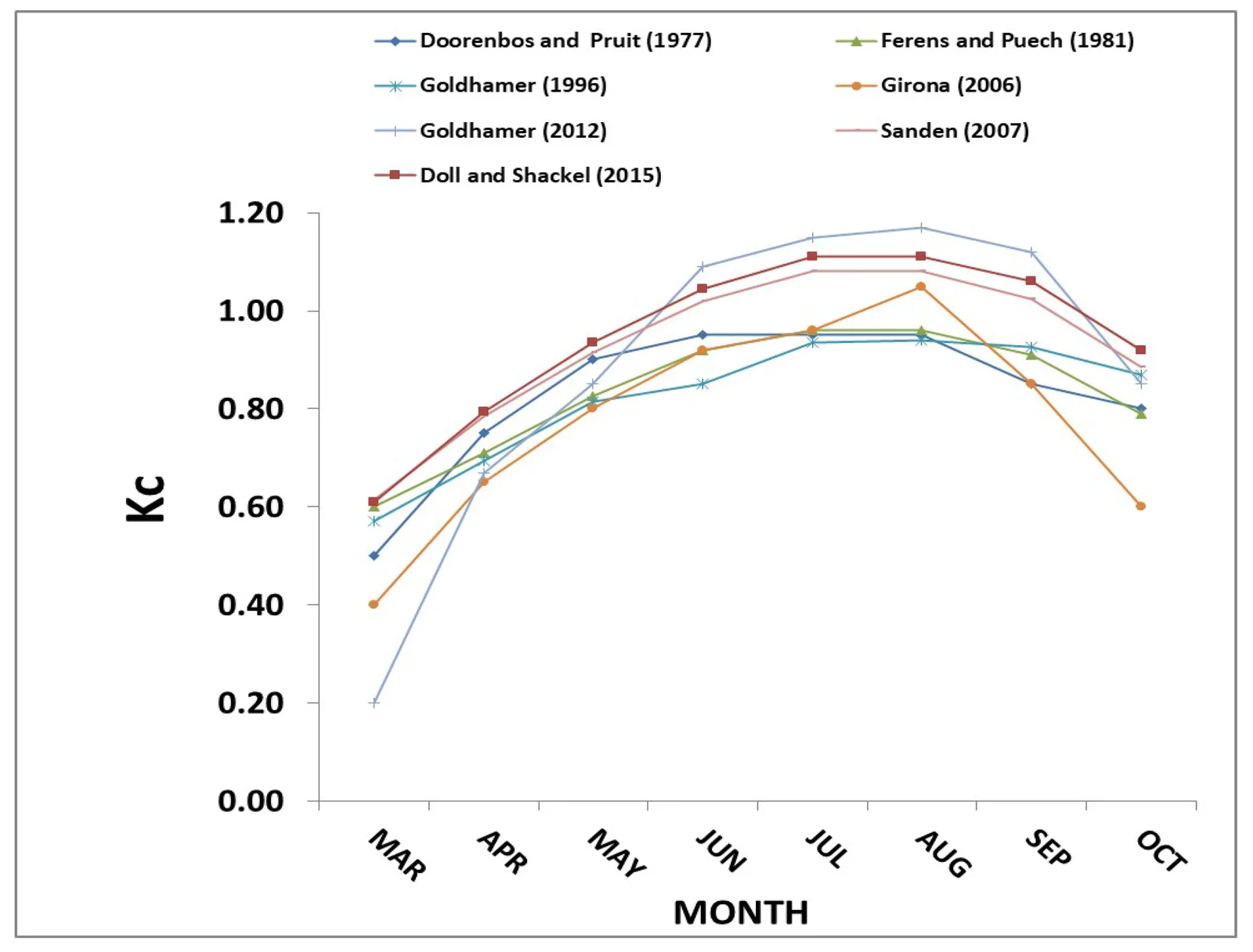
Monthly crop coefficient (Kc) curves for mature almond orchards compiled by the authors from various published sources, including Goldhamer and Girona (2012) [41], Doll and Shackel, 2015 [42], Doorenbos and Pruitt (1977) [43], Sanden (2007) [44], Fereres and Puech (1981) [48], Girona (2006) [49], and Goldhamer (1996) [50], as reported by Steduto et al. (2012) [40].

In mature almond orchards, Kc varies seasonally, generally reaching its highest values during the mid-season period and declining after harvest as a result of leaf senescence Figure 3 presents the estimated monthly Kc values during the growing season [40,41,42,43,44,48,49,50]. Reported maximum Kc values are generally in the range of 0.95–1.08, although values exceeding 1.1 have been observed under high-density or intensively managed conditions [41,42]. These variations highlight the importance of considering orchard age, canopy cover, planting density, irrigation system, and management practices when estimating almond water requirements. The crop evapotranspiration of almond orchards is influenced not only by climatic conditions but also by orchard characteristics, including tree age, canopy size, planting density, and crop load [39]. The net irrigation requirement is subsequently determined by accounting for effective rainfall and other water inputs, as well as soil-water storage and water losses. Therefore, Kc values should not be considered universally applicable but should instead be interpreted according to the specific climatic, phenological, and orchard-management conditions under which they were derived.

Almond yield generally increases with water availability up to an optimum, beyond which additional irrigation provides limited benefits or may reduce yield. In California, ‘Nonpareil’ reached this threshold at approximately 85–90% ETc [7]. In Spain, Mirás-Avalos et al. (2023) [26] reported strong effects of total water input on ‘Guara’ yield, with cultivar-specific differences in water-use efficiency and response to water availability.

## 4. Growth Dynamics of Almond Trees and Fruit Development

**Figure 4:**
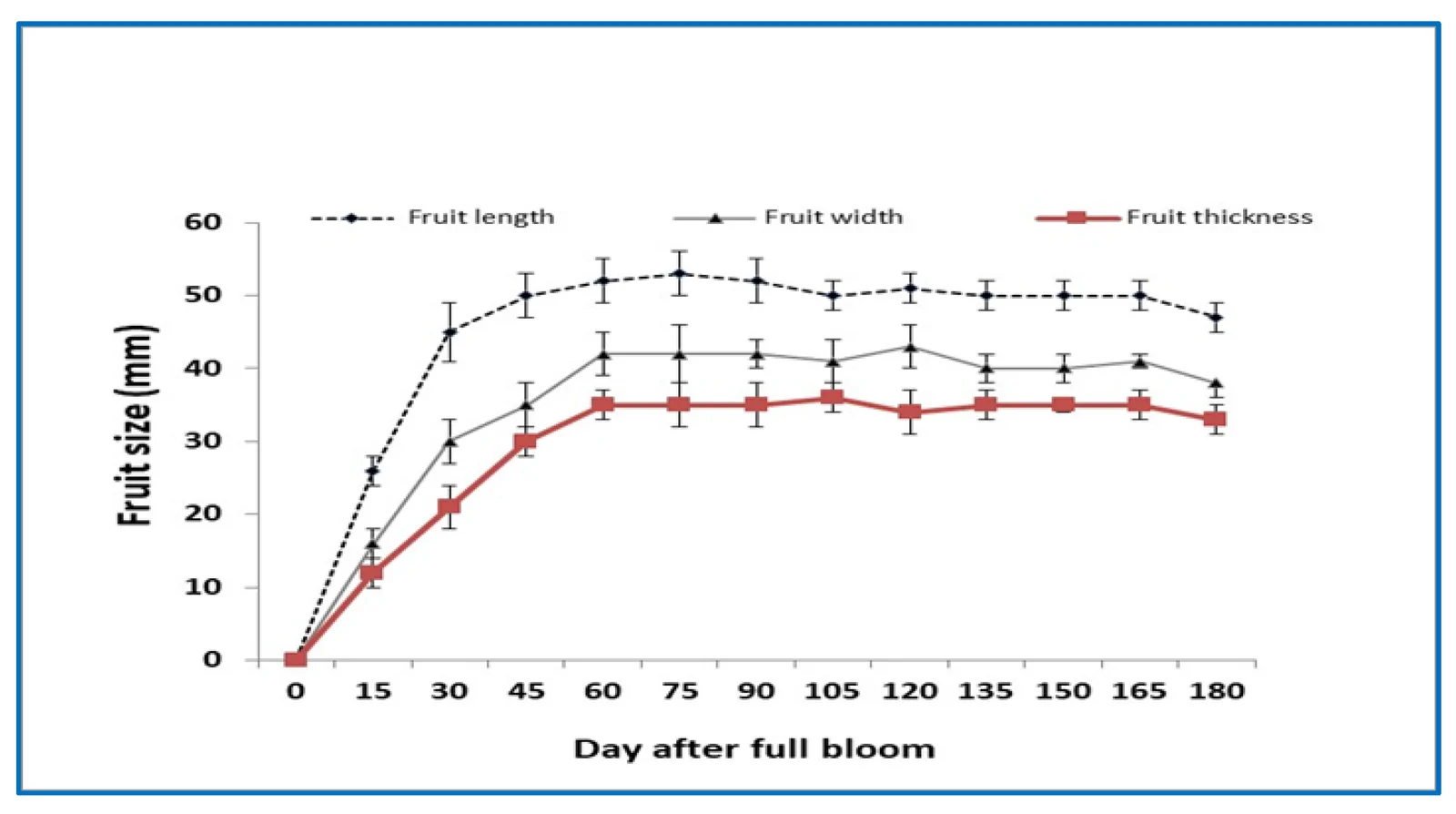
Fruit size parameters (length, width, and thickness) and growth dynamics in the almond cultivar ‘Tuono’ (source: Tarantino et al., 2025 [51]).

**Figure 5:**
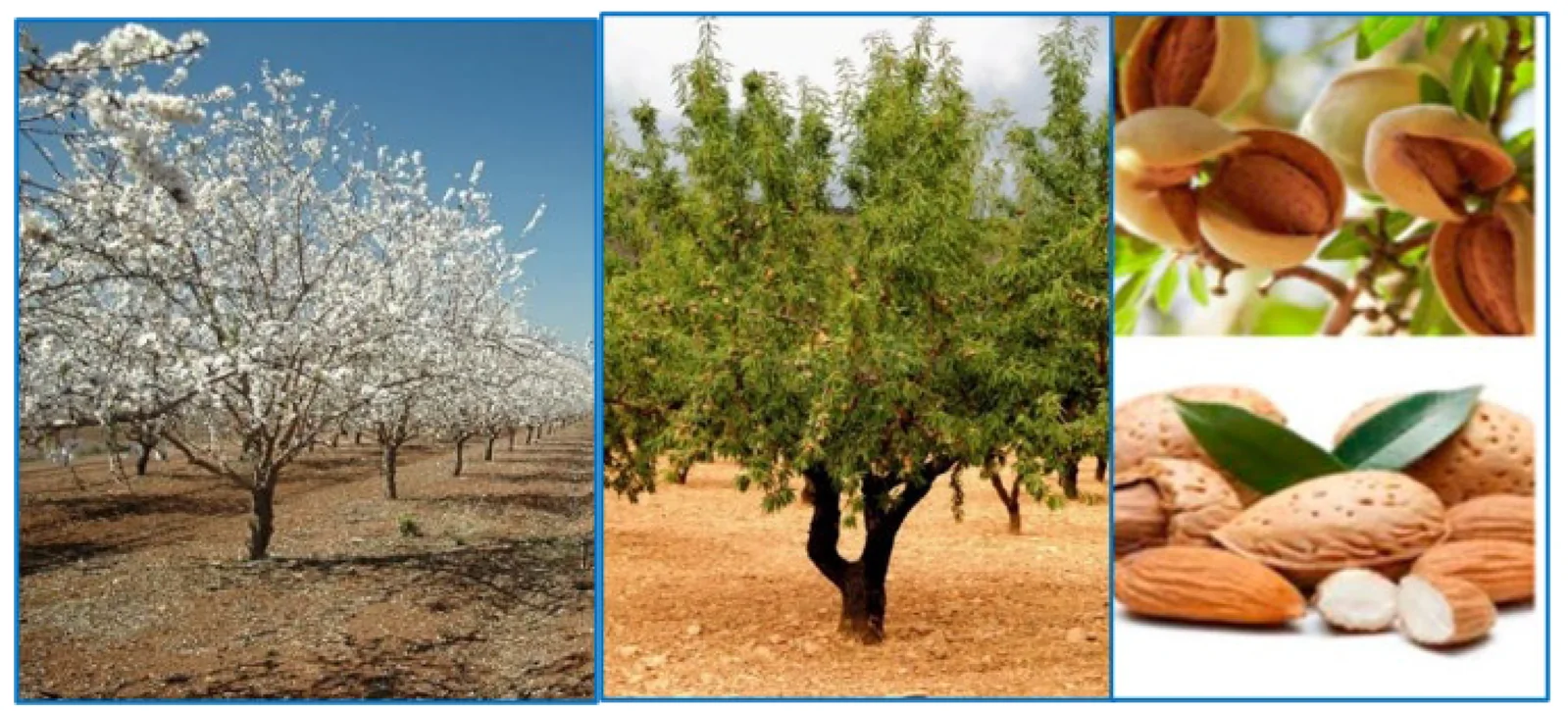
*Prunus dulcis* trees, blooms and fruits (adapted and modified from Massantini et al., 2022 [27]).

Almond trees (*Prunus dulcis*) undergo distinct vegetative and reproductive phases, with seasonal carbohydrate allocation among vegetative growth, fruit development, and storage reserves. Due to their relatively low chilling requirements, flowering and leaf emergence occur early, generally between late January and March in the Northern Hemisphere, depending on the cultivar and climatic conditions. Following bud break and fruit set, rapid vegetative growth and fruit development occur, with fruit size approaching its maximum within approximately 50–55 days, generally during March–April (Figure 4) [51]. Thereafter, assimilates are increasingly allocated to kernel filling and maturation, which generally occur during August–September (Figure 5). After harvest, carbohydrates are accumulated as storage reserves, contributing to growth and productivity during the subsequent season. Reserve accumulation is also associated with flower-bud formation and may be influenced by excessive vegetative growth [52,53].

**Figure 6:**
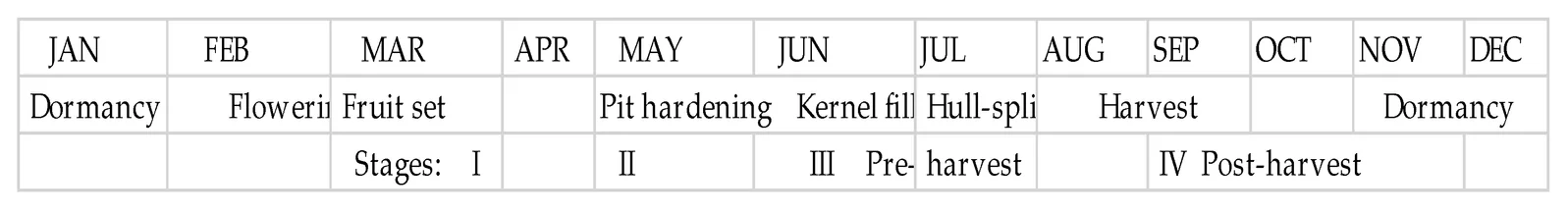
Simplified almond fruit developmental stages commonly referenced in agronomic and water-management studies. Timing may vary depending on cultivar, climate, and local management practices.

In Mediterranean arid and semi-arid climates, drought, high temperatures, and limited water availability can substantially reduce the water status of almond trees and, under severe conditions, cause defoliation and tissue damage [54,55]. Almond trees generally acclimate better to gradual water deficits than to abrupt changes in water availability [56,57]. Their response to water stress varies according to the phenological stage; therefore, the production cycle is commonly divided into four stages: Stage I, flowering and early fruit development; Stage II, rapid fruit growth and shell hardening; Stage III, kernel filling and maturation; and Stage IV, postharvest reserve accumulation and flower-bud differentiation (Figure 6) [7,58,59,60].

## 5. Application of Deficit Irrigation

When irrigation water is available throughout the growing season, for example from on-farm reservoirs or collective water supply systems, DI deliberately supplies less water than the full crop evapotranspiration requirement [28,61]. This strategy aims to improve water-use efficiency (WUE) and promote more sustainable irrigation practices in arid and semi-arid regions, particularly in the Mediterranean basin, where recurrent droughts, climate change, and increasing competition for water among agricultural, urban, industrial, and tourism sectors place growing pressure on water resources during the summer months [62,63,64,65,66].

Depending on the timing, intensity, and method of water reduction, four major DI strategies have been investigated in almond cultivation:

[I] Regulated Deficit Irrigation (RDI): Water deficits are strategically imposed during phenological stages that are relatively tolerant of water stress, while full irrigation is maintained during critical periods of crop development [25,26,67,68,69]. This approach aims to minimize the impact of water deficits on yield while improving irrigation water productivity.

[II] Sustained Deficit Irrigation (SDI): A constant fraction of the full irrigation requirement is applied throughout the growing season [58,70]. Because irrigation is continuously reduced, plant water stress gradually increases over the season as soil water reserves become progressively depleted [71,72].

[III] Partial Root-Zone Drying (PRD): PRD can be implemented under either sustained or regulated irrigation schedules. This technique alternates irrigation between opposite sides of the root system, allowing one portion of the root-zone to dry while the other remains adequately watered [28,73,74]. Unlike SDI and RDI, which primarily reduce water use by imposing controlled soil-water deficits, PRD exploits root-to-shoot chemical signaling. Roots in the drying portion of the soil synthesize abscisic acid (ABA), which is transported through the xylem to the leaves, where it induces partial stomatal closure and reduces stomatal conductance, while the irrigated portion of the root system continues to maintain an adequate plant water status [75,76].

[IV] Low-Frequency Deficit Irrigation (LFDI): LFDI consists of applying irrigation at longer intervals, allowing the soil to dry to predetermined physiological threshold values before irrigation is resumed [77]. This strategy has shown promising results in almond orchards, particularly when applied during the kernel-filling stage, with long-term studies reporting substantial water savings without significant reductions in yield. However, its implementation requires continuous monitoring of plant water status to identify appropriate irrigation thresholds, making it technically demanding and often difficult for growers and irrigation managers to adopt in commercial orchards [77].

## 6. Physiological Responses to Water Stress

**Figure 7:**
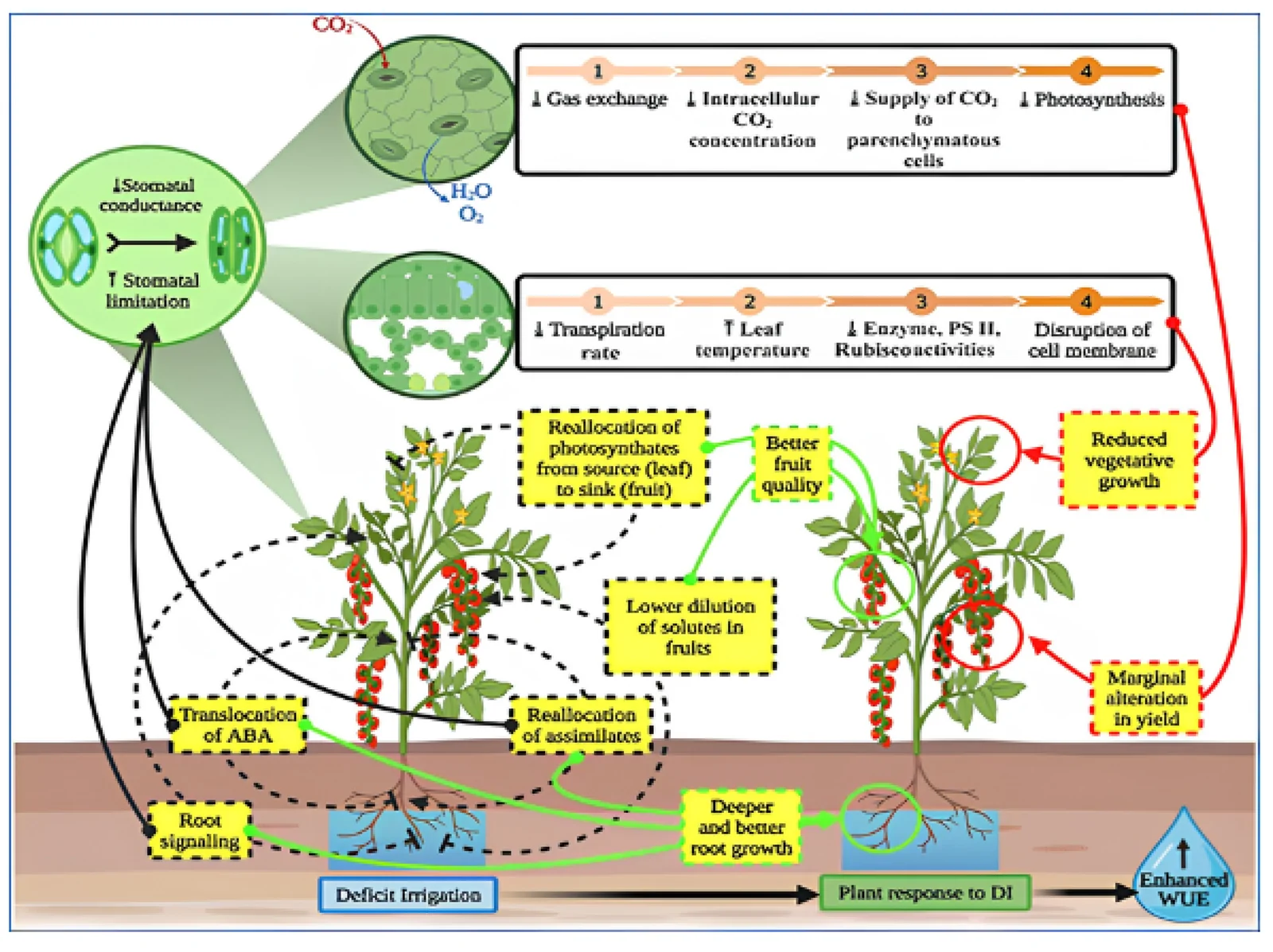
Relationship between deficit irrigation (DI) and plant physiology (source: Champaneri et al. 2024 [82])As water stress intensifies, shoot growth and fruit set may decline, while fruit abscission can increase, potentially reducing yield [68]. The effects of water stress on photosynthesis and productivity depend on stress intensity and duration [84,85]. Almond is generally classified as an anisohydric species and can maintain relatively high stomatal conductance and carbon assimilation under moderate water deficit [62,86]. Leaf water potential Ψ_leaf_ may decline substantially before marked reductions in gas exchange occur, indicating that drought responses extend beyond stomatal regulation [87]. Therefore, reliable physiological indicators and stress thresholds are important for optimizing DI strategies while minimizing effects on growth, yield, and nut quality.

Plants respond to water stress through coordinated morphological, physiological, biochemical, and molecular mechanisms [78]. In *Prunus* species, drought-responsive mechanisms promote root development and structural adaptations that improve water acquisition and reduce water loss [28,79]. Physiological responses are mainly regulated by endogenous hormones, particularly abscisic acid (ABA), which mediates stomatal closure and regulates photoassimilate allocation (Figure 7) [80,81,82]. These responses reduce transpiration and help maintain photosynthetic activity under moderate water deficits. Biochemical adjustments, including osmotic regulation and antioxidant responses, further contribute to cellular protection under water stress. Overall, these mechanisms enhance water-use efficiency (WUE) by improving the balance between carbon assimilation and water loss and strengthening plant adaptation to limited water availability [83].

## 7. Water Stress Assessment

Accurate monitoring of crop water status is essential for improving irrigation management and developing effective strategies to mitigate water stress. Current approaches for monitoring plant water status (PWS) can generally be classified into direct and indirect (remote-sensing (RS)methods [88].

### 7.1. Direct Measurement

**Figure 8:**
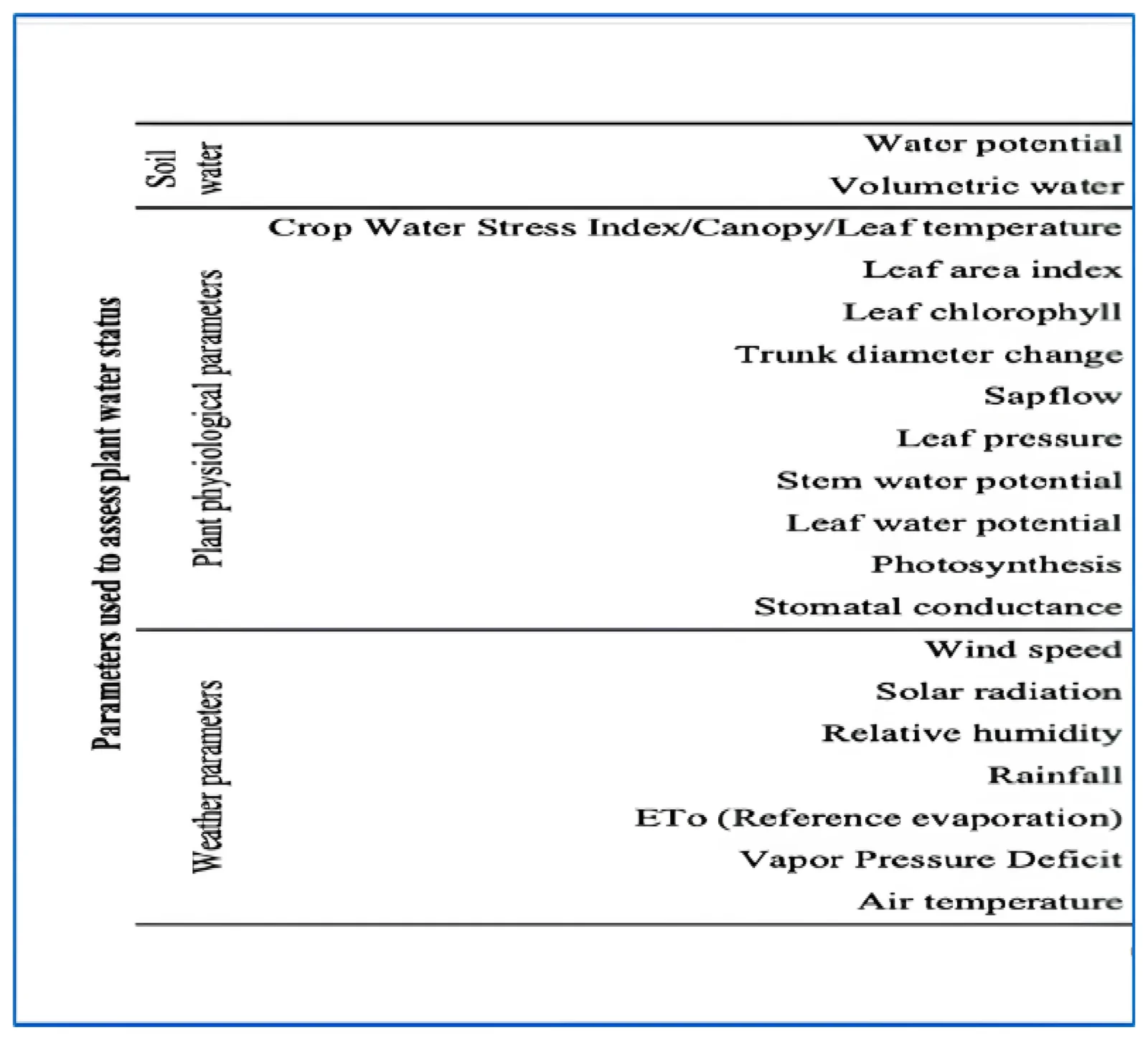
Parameters used for plant water status monitoring or irrigation management (source: Velazquez-Chavez et al. 2024 [89]).

Direct measurements integrate soil-, plant-, and atmosphere-based indicators (Figure 8) [89] and may be complemented by remote-sensing (RS) approaches, depending on the required accuracy, cost, and spatial scale. Combining indicators from different categories can provide a more comprehensive assessment of plant water status (PWS) [89].

Soil-based indicators, including root-zone water potential (Ψr) and volumetric water content (VWC), provide information on rhizosphere water availability and are commonly measured using soil-moisture sensors and related techniques. However, their accuracy can be affected by soil and environmental conditions, and they provide an indirect estimate of PWS. Consequently, plant-based measurements are increasingly used to provide a more direct assessment of plant water status through physiological responses [90].

**Figure 9:**
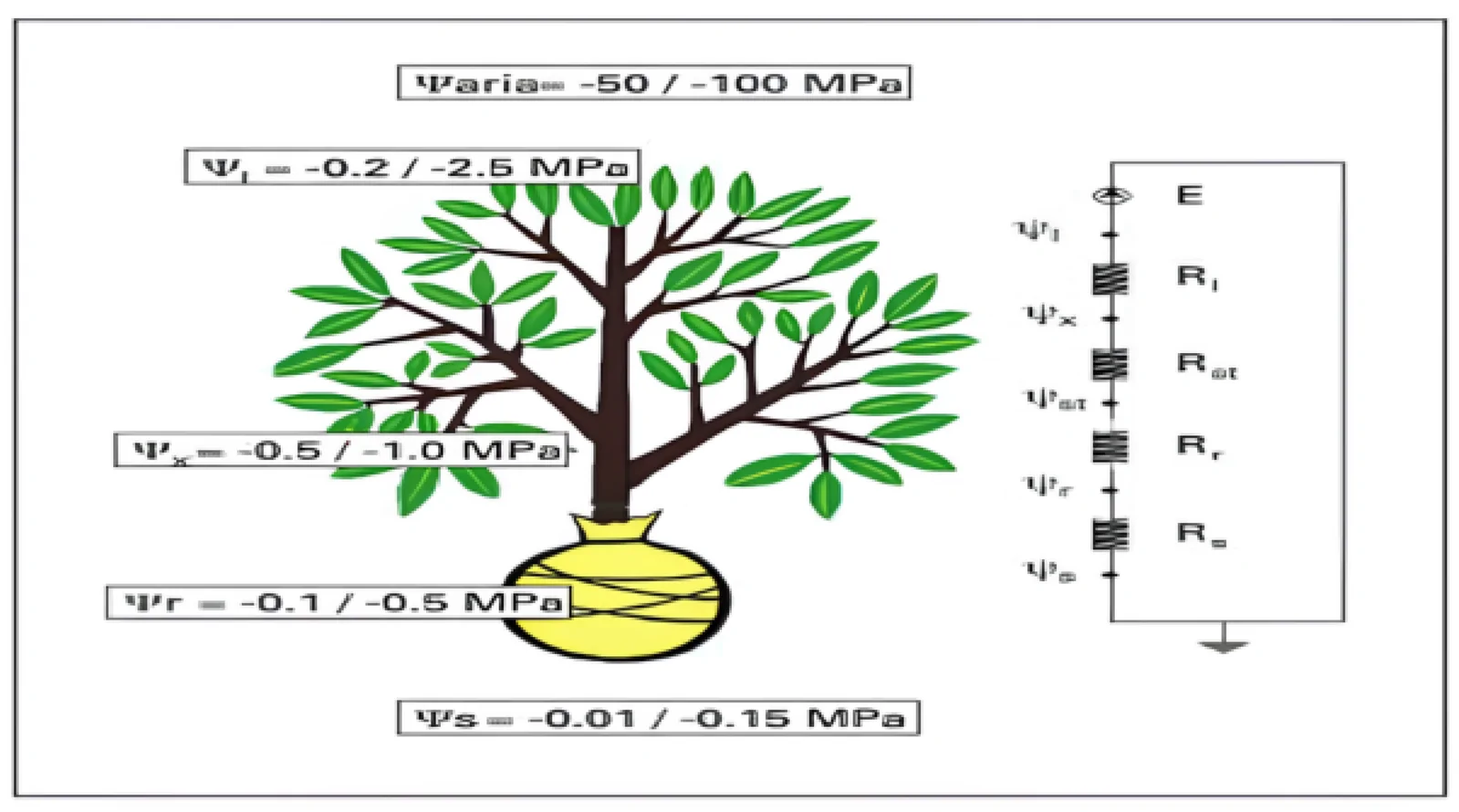
Description of the soil–plant–atmosphere continuum through the analogy with an electrical circuit. Ψs = soil water potential; Ψr = root water potential; Ψx = xylematic water potential; Ψl = leaf water potential; Ψa = water potential of the atmosphere; Rs = soil resistance; Rr = radical resistance; Rst = stem resistance; Rl = leaf resistance; E = external. environment (source: Mugnai 2004 [91]).

This shift is grounded in the principles governing water transport through the soil–plant–atmosphere continuum (SPAC), where plant water status reflects the balance between soil water availability and atmospheric demand. Water moves along water-potential gradients from the soil through the plant to the atmosphere (Figure 9), driven by the negative pressure generated by transpiration [91]. Thus, plant water potential integrates soil water availability and atmospheric evaporative demand, providing a physiologically relevant indicator of crop water status.

Xylem structure reflects a trade-off between hydraulic efficiency and embolism resistance: larger vessels increase hydraulic capacity but cavitation risk, whereas smaller vessels increase resistance at the expense of efficiency [92,93]. Cavitation can impair xylem hydraulic function and plant water transport [94]. P_50_, defined as the xylem water potential causing a 50% loss of hydraulic conductivity, is widely used to assess embolism resistance, with more negative values indicating greater resistance. In almond, ‘Soleta’ and ‘Isabelona’ show P_50_ values of approximately −3.7 to −3.8 MPa, compared with −2.97 MPa for ‘Avijor’ and ≈−3.2 MPa for wild genotypes [93,95]. Xylem anatomy and P_50_ are therefore relevant indicators of drought tolerance and selection traits.

Plant physiological measurements provide integrative indicators of PWS and complement soil- and atmosphere-based approaches. Their application to DI strategies is discussed in Section 8.1. However, proximal sensing can be labor-intensive, costly, and difficult to scale, while leaf-level measurements may not represent whole-plant water status or orchard-scale variability [90,96,97,98,99]. Scalable technologies are therefore needed for large-area PWS monitoring.

Irrigation scheduling traditionally uses meteorological variables to estimate crop water requirements through ETo, ETc, and soil water balance models, while WP assesses irrigation efficiency [100]. However, these approaches do not directly capture plant water status or its spatial variability resulting from differences in tree physiology, canopy, soil, and microclimate. This limitation supports the development of scalable technologies integrating plant responses and spatial information for precise and adaptive irrigation management.

### 7.2. Indirect (Remote) Methods

**Figure 10:**
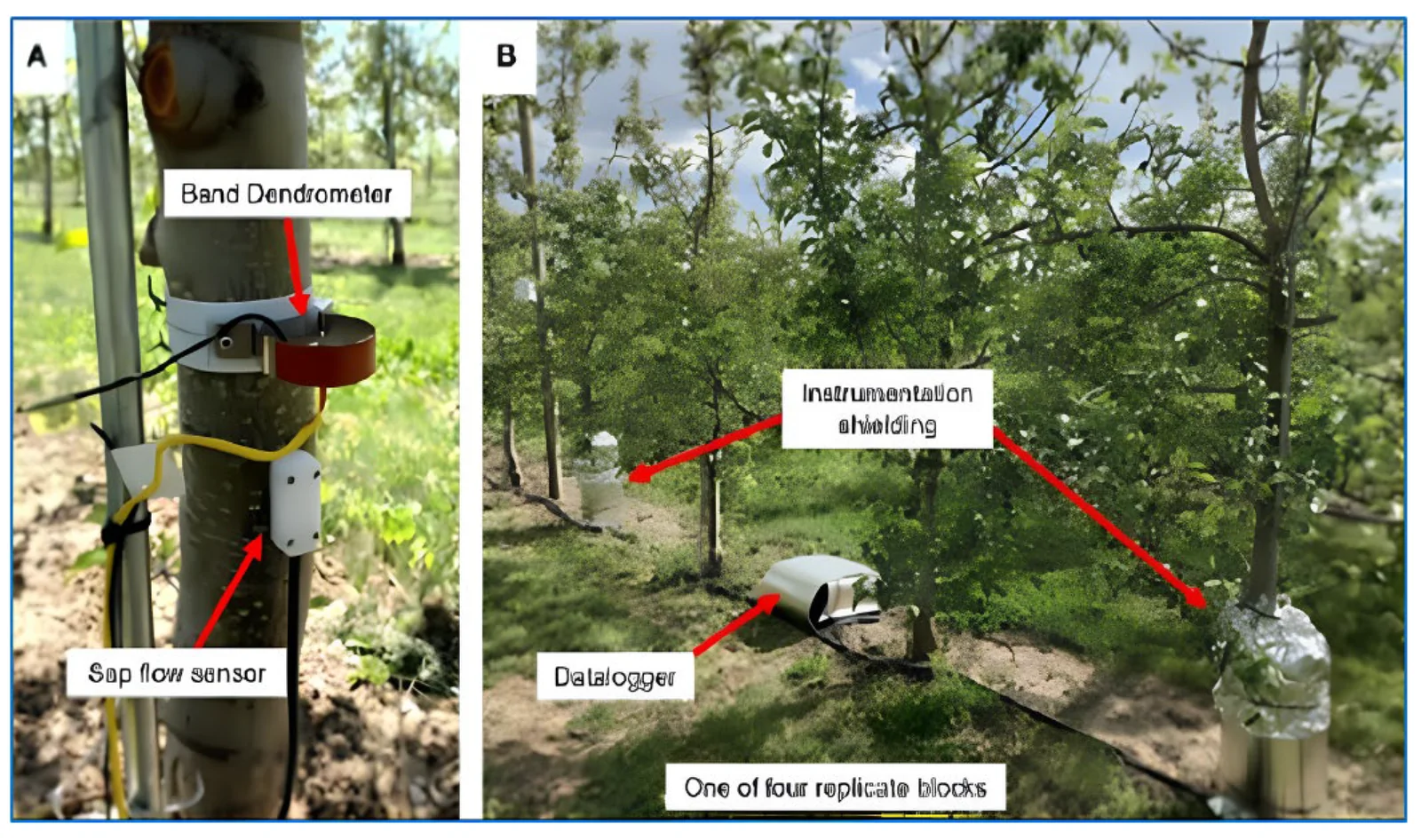
(**A**) A band dendrometer (above) and sap flow sensor (below). (**B**) Dataloggers with insulation (center box) connected to two sensor installations with thermal and protective shielding (left and right of center box) (source: Kishore et al. 2025 [105]).

To address these limitations, recent advances in crop water status monitoring have increasingly focus on RS- and plant-based sensor technologies. High-resolution thermal and multispectral imaging, combined with proximal plant sensors, enables rapid and non-destructive assessment of water stress over large areas. Furthermore, the integration of these technologies with the Internet of Things (IoT) and wireless sensor networks (WSNs) (Figure 10) facilitates continuous, real-time monitoring of crop water status, thereby improving irrigation decision-making and enabling remote management under DI conditions [101,102,103,104]. The application of these technologies within DI strategies is discussed in Section 9.

## 8. Deficit Irrigation in Almonds: Review of the Literature

The DI strategies, including RDI, SDI, LFDI, and PRD, used in almond cultivation in arid and semi-arid regions were reviewed. Field experiments conducted over one or more growing seasons were evaluated to assess the effects of these irrigation strategies on physiological responses, yield, nut quality, vegetative growth, water productivity, and economic performance.

Most of the reviewed studies compared full irrigation (FI; approximately 100% ETc) with DI treatments ranging from very mild (85% ETc) and mild (70–75% ETc) to moderate (60% ETc), moderate-to-severe (50% ETc), severe (40% ETc), and, in some cases, very severe (20–25% ETc) water deficits or irrigation cut-off treatments. Where available, additional irrigation regimes, including rainfed (RF), farmer (FRM) and over irrigated (OI) conditions, were also considered. The selected studies encompassed a wide range of experimental conditions, including trials involving young, non-productive trees [106,107], young, productive trees [56,58,108,109], mature orchards [71,110,111,112,113], and multiple cultivars [114,115,116,117]. More recent studies have incorporated RS technologies for monitoring plant water status, providing innovative approaches for evaluating tree responses to DI [87,118,119,120].

A total of 63 articles met the selection criteria. For each study, key experimental information, including study location and country, irrigation treatments and timing of application, cultivar and tree age, study duration, planting density, and main findings, was extracted and summarized in Table 2.

Table 2 provides a concise overview of the main characteristics of the studies included in the review and is not intended to provide an exhaustive representation of all variables considered throughout the manuscript. In particular, economic indicators and the use of remote sensing are not reported as separate columns in the table, as these aspects are examined in greater detail in Section 8.10 and Section 9, respectively. Thus, the absence of these variables from Table 2 reflects the scope and concise format of the table rather than their exclusion from the review.

The following sections first examine the effects of DI on physiological indicators of plant water status. This is followed by an evaluation of the effects of RDI applied at specific phenological stages and under different levels of water deficit. Separate analyses are then conducted for SDI, LFDI, and PRD. Finally, these irrigation strategies are compared with RDI to provide an integrated assessment of their suitability for almond production under water-limited conditions.

### 8.1. Effects of DI on Physiological and Biochemical Indicators of Plant Water Stress

Key physiological indicators of plant water status include Ψleaf, Ψstem, gs, An, Tr, leaf and canopy temperature, sap flow, and hydraulic and dendrometric traits [62,89,100,113,125,135,159,160]. Biochemical indicators include Fv/Fm, osmotic adjustment, ABA, proline, phenolics, flavonoids, antioxidant enzymes (SOD, CAT and POD), MDA and electrolyte leakage [120,161,162]. Together, these indicators characterize plant water relations, photosynthesis, osmotic regulation, membrane stability and antioxidant responses.

*Leaf and Stem Water Potential*

**Figure 11:**
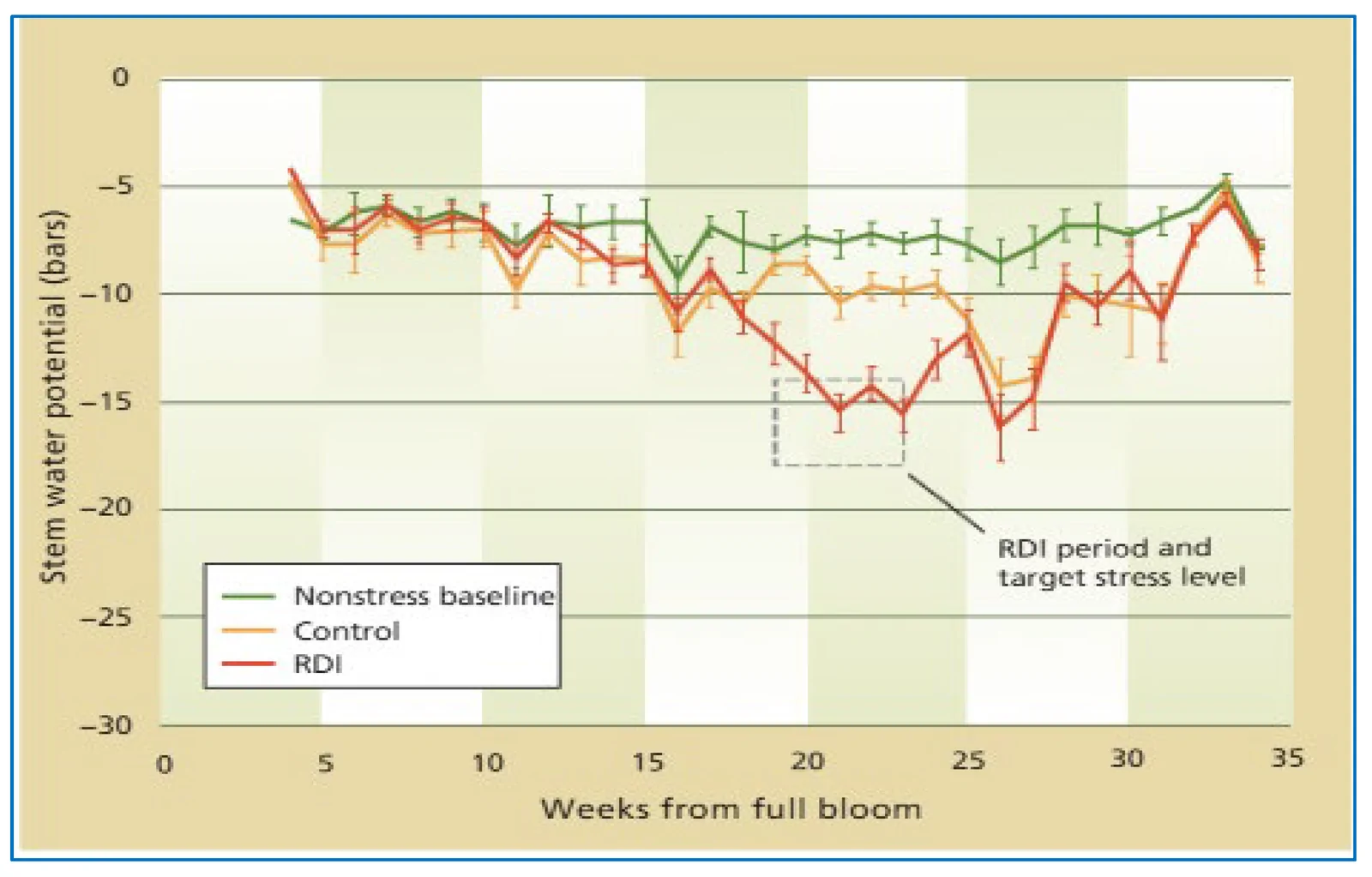
Seasonal average pattern of stem water potential (Ψstem) values for control and RDI treatments, with upper and lower limits of target water stress and average duration of RDI regime, and seasonal average expected Ψstem values for nonstressed almond trees, 2004–2008. Nonstressed baseline values (source: Shackel et al., 1998 [106]) were calculated using te California Irrigation Management Information System (CIMIS) data for atmospheric vapor pressure deficit (source: Stewart et al., 2011 [132]).

Midday stem water potential (Ψms) is widely used for almond irrigation scheduling because it integrates tree water status and is less sensitive to short-term atmospheric variation than Ψml or Ψpd [108,122,163,164,165]. Under well-watered conditions, Ψms is generally −0.5 to −1.0 MPa but may reach ≈−1.5 MPa under high VPD without indicating soil-water stress [111,140]. Seasonal Ψstem commonly declines from ≈−1.1 MPa in spring to −1.5 MPa in midsummer and ≈−1.7 MPa later in the season, while Ψml is generally −1.0 to −1.5 MPa [120,153,154,166]. Moderate and severe stress are commonly associated with Ψstem below −1.7 MPa and around −2.6 to −3.0 MPa, respectively; however, VPD strongly affects Ψstem, making the nonstressed baseline relationship (Ψstem = −0.12 × VPD − 0.41 MPa) preferable to fixed thresholds [57,167]. In mature ‘Nonpareil’, full irrigation maintained Ψstem within the well-watered range, whereas RDI50 reduced preharvest Ψstem to −1.4 to −1.8 MPa, demonstrating its sensitivity to irrigation regime (Figure 11) [132].

Moderate RDI also reduced Ψstem and gs by 30–60%, while severe RDI reduced Ψstem to −2.5 to −3.5 MPa. Moderate RDI caused only minor yield reductions (<10–15%) while saving 20–40% water, whereas severe stress reduced yield by 15–30% [109]. Water deficits trigger stomatal closure, increased canopy temperature and reduced carbon assimilation. In young ‘Nonpareil’, Ψml remained above −4.0 MPa even under severe stress, indicating hydraulic regulation, while mature ‘Marta’ showed declines in Ψleaf from ≈−1.1 MPa under FI to −1.6 to −2.0 MPa under LFDI and RDI50 [77]. Prolonged stress in ‘Nonpareil’ reduced Ψms to ≈−2.6 MPa, CO_2_ assimilation and canopy light interception through premature defoliation [111]. PRD50 maintained higher An than RDI50 despite similar gs, suggesting improved intrinsic water-use efficiency [62]. Under severe preharvest irrigation cut-off, Ψpd reached ≈−4.0 MPa, with increasing yield losses as water stress intensified [112,123]. Overall, DI responses depend mainly on deficit severity, duration and timing, progressing from stomatal regulation to reduced photosynthesis, higher canopy temperature, defoliation and yield loss.

*Stomatal conductance*

Stomatal conductance (gs) is an early drought-response indicator; its reduction limits transpiration and CO_2_ uptake, balancing water conservation and carbon assimilation [68,108]. Under FI, gs generally declines seasonally from 0.40–0.70 to 0.15–0.25 mol m^−2^ s^−1^, reaching 0.05–0.09 under severe drought [113,114,142,154,168,169]. Responses vary with genotype and environment [165]. Severe stress may also induce defoliation and senescence, reducing photosynthetic and reproductive capacity [170]. Moderate RDI can maintain gs during kernel filling while reducing water use, with limited cumulative stress and recovery after re-irrigation.

*Net Photosynthesis*

Under FI, An generally declines from ≈20 to 12 μmol CO_2_ m^−2^ s^−1^, compared with ≈20 to 10 under DI [120]. RDI can reduce An to 4–5 μmol CO_2_ m^−2^ s^−1^, while extreme drought may reduce it to 2.4–2.6 μmol CO_2_ m^−2^ s^−1^ [112,142,167]. Under moderate stress, reduced An is mainly associated with stomatal limitation; with increasing drought, biochemical and photochemical limitations become more important, causing reductions of 55–70% [113,122,153,161,171,172]. These limitations are more pronounced when gs falls below 0.10–0.15 mol m^−2^ s^−1^ or Ψstem below −2.0 MPa [173].

Phenological stage also affects An, which typically declines from 16–20 μmol CO_2_ m^−2^ s^−1^ during early growth to 8–10 μmol CO_2_ m^−2^ s^−1^ during kernel filling [95,111,169], partly due to reduced sink demand and limitations in TPU and Jmax [154]. Thus, physiological thresholds should be interpreted according to developmental stage.

Moderate SDI_75_–SDI_65_ can reduce irrigation from ≈770 to 574–516 mm without significant yield penalties in ‘Guara’, ‘Marta’ and ‘Lauranne’ [154]. Severe deficits reduce gs, An and transpiration while increasing intrinsic WUE and senescence, although some physiological activity persists under extreme drought [142]. In ‘Ferragnès’, severe SDI showed variable performance across years, whereas kernel-filling RDI maintained yields comparable to FI with substantial water savings. Overall, photosynthetic responses depend on deficit severity, timing and climatic conditions.

*Canopy and Leaf Temperature*

Water stress causes stomatal closure and reduced transpirational cooling, increasing Tc and Tleaf, which can therefore indicate tree water status [83,173,174,175]. In almond, infrared thermography and CWSI can assess water status and estimate Ψms under DI [143]. UAV thermal imaging enables spatial monitoring of orchard water stress, with aerial CWSI improving irrigation-zone delineation compared with NDVI or ETc alone [176] CWSI has also been used to distinguish drought-tolerant almond rootstocks [26]. Canopy thermal variability may increase under moderate stress but decline under severe stress as stomatal closure becomes more uniform, making thermal patterns useful for tracking drought development [177].

*Transpiration rate*

Transpiration (Tr) decreases as VPD and water stress increase, through stomatal closure and reduced gs, ultimately increasing Tleaf [27,172]. Under severe drought, Tr may decline from ≈8 to <0.5 mmol H_2_O m^−2^ s^−1^, with cultivar-dependent responses [168,169]. High-density hedgerow systems can also reduce transpiration and improve water productivity compared with open-vase systems [30]. Overall, Tr reflects the integrated effects of water availability, Ψstem, gs, An, genotype and orchard management [133].

*Sap flow*

Sap flow (SF) provides continuous, non-destructive monitoring of tree water use and may detect drought responses earlier than periodic Ψstem measurements [30,178,179]. Under DI, SF declines with stomatal closure and, under prolonged stress, reduced hydraulic conductivity and xylem transport [180,181,182]. In almond, reduced Tr has been associated with Ψstem values of approximately −1.1 to −1.2 MPa, suggesting a potential irrigation threshold [66,179].

In ‘Guara’, SF-based transpiration estimates agreed well with water-balance calculations (R^2^ = 0.81), while transpiration coefficients declined from ≈1.0 under FI to 0.33 under severe DI [183]. Combining SF with TSEB improved orchard-scale estimates of transpiration and canopy conductance [66]. Overall, SF is useful for linking tree water use with physiological responses and supporting precision irrigation.

*Dendrometric Indicators*

Continuous trunk diameter variation (TDV), measured with LVDTs, provides non-destructive monitoring of tree water status. Maximum daily trunk shrinkage (MDS) and its normalized intensity (SIMDS) indicate increasing water stress, with values of ≈1.3–1.6, 2.2–2.8 and >2.8 corresponding to mild, moderate and severe stress [135,180,184]. Daily trunk growth rate (TGR) complements MDS, with >0.3, 0.1–0.3 and <0.1 mm day^−1^ indicating low, moderate and high stress [107,185]. Deficit irrigation increases trunk contraction while reducing Ψstem, gs, An, growth and yield, supporting TDV indicators for continuous irrigation monitoring [120].

*Biochemical indicators*

Deficit irrigation induces biochemical adjustments associated with drought adaptation. In ‘Guara’, increasing DI reduced Ψleaf, gs, An and Tr, while soluble sugars, ABA and protein content changed with increasing stress; mild RDI_70_ maintained relatively high WUE, whereas severe SDI_50_ reduced yield [129]. In ‘Lauranne’, rainfed conditions reduced RWC, gs, An and Tr but increased intrinsic WUE and ABA and IAA, while RDI_35_ during fruit filling-maintained yield with improved WUE [138]. Progressive TPU limitations during kernel filling and postharvest further indicate reduced photoassimilate demand and support these stages as potential periods for RDI [154].

### 8.2. Comparative Assessment of Deficit Irrigation Strategies

The comparative assessment considers regulated deficit irrigation (RDI), sustained deficit irrigation (SDI), and partial root-zone drying (PRD) in relation to almond yield response under water-limited conditions. The comparison focuses not only on the irrigation strategy itself, but also on the amount, severity, and seasonal timing of the water deficit, as these factors may interact and influence yield responses. A key distinction emerging from the reviewed literature is therefore between the quantity of water applied and the way in which water restriction is imposed over space and time. RDI allows water stress to be deliberately concentrated in selected phenological stages, whereas SDI maintains a more continuous level of deficit throughout the season. PRD differs from both approaches by alternating irrigation between portions of the root-zone, thereby creating spatially heterogeneous soil-water availability. This distinction is important when interpreting comparative results, because apparent differences among irrigation strategies may partly reflect differences in total seasonal water supply, deficit intensity, or the phenological period during which stress occurs. Comparisons based on similar water inputs therefore provide a more appropriate basis for assessing whether the irrigation strategy itself contributes to differences in almond yield.

Comparative evidence synthesis: Across the reviewed studies, deficit severity and timing generally appear to have a stronger influence on almond yield than the irrigation strategy itself when comparable amounts of water are supplied. RDI provides greater control over the timing of water stress, SDI imposes a more continuous seasonal deficit, and PRD creates spatially heterogeneous soil drying. However, the available evidence does not demonstrate a consistent yield advantage of one strategy over another under comparable water inputs. The evidence that deficit severity and timing are major determinants of yield response is well-supported, whereas the broader conclusion that irrigation strategy per se has a smaller effect than water amount and timing is moderately supported because direct comparisons remain limited. In particular, the comparative advantage of PRD over RDI remains unresolved, and cultivar-specific responses prevent identification of a universal optimum deficit level.

#### 8.2.1. RDI During Flowering, Fruit Set and Initial Growth (Stage I)

Limited research has investigated the effects of water stress during Stage I and, to a lesser extent, Stage II of almond fruit development. This is partly because, in Mediterranean almond-growing regions, precipitation is generally concentrated from autumn to early spring, maintaining soil-water availability during flowering, fruit set, and early vegetative growth. Moreover, evaporative demand and canopy development are relatively low during the early part of the season. Consequently, winter and early-spring soil-water reserves can buffer trees against early-season water deficits, making severe water stress during Stage I relatively uncommon under Mediterranean conditions.

Nevertheless, water stress during floral bud development and around bloom can affect subsequent reproductive performance and yield, emphasizing the importance of the timing of water deficits in almond [55]. Carbohydrate reserves accumulated during the previous growing season also contribute to the tree’s capacity to support growth and reproduction in the following season. Water stress during the previous harvest period can deplete these reserves and subsequently affect yield determinants in mature almond trees [112].

During Stage I, fruit growth and canopy development occur concurrently and depend on the tree’s available assimilate supply. Consequently, adequate water availability is important for maintaining growth and yield, although almond responses depend strongly on the timing and severity of water stress [33]. Water stress during Stage I can adversely affect both reproductive and vegetative development. Early-season water deficits can increase fruit abscission and restrict fruit and vegetative growth, potentially reducing canopy development and the photosynthetic capacity available to support subsequent growth and fruit development [121]. Girona et al. (1997) [122] further demonstrated that almond responses to water stress vary according to the timing of the deficit, with physiological, growth, and yield responses differing among seasonal stress periods [122].

These findings indicate that, although severe early-season water deficits are relatively uncommon in Mediterranean environments, substantial stress during sensitive reproductive and early-growth periods can compromise fruit retention, vegetative development, and yield potential.

Evidence synthesis: Overall, the evidence indicates that severe water stress during flowering, fruit set, and early vegetative growth can adversely affect reproductive development, fruit retention, and vegetative growth. However, the number of controlled studies specifically addressing Stage I RDI remains limited, and responses depend on stress severity, timing, cultivar, and pre-season soil-water availability. Therefore, the evidence that severe Stage I water stress can impair reproductive and vegetative development is moderate, whereas evidence is insufficient to define a universal Stage I yield-penalty threshold or an optimal RDI level. This limitation is particularly relevant under Mediterranean conditions, where winter and early-spring soil-water reserves may buffer moderate early-season water deficits.

#### 8.2.2. RDI During Rapid Fruit Growth and Pit Hardening (Stage II)

As seasonal evaporative demand increases, almond shoot, spur, and fruit growth become progressively more susceptible to water deficits in the absence of irrigation or rainfall. Consequently, adequate water availability during Stage II is critical for maintaining vegetative growth, fruit development, and final yield. Water stress immediately after fruit set restricts turgor-driven cell expansion and assimilate transport, resulting in smaller fruits, reduced kernel weight, and greater crop-load instability [130,186].

Field studies have consistently demonstrated the adverse effects of water deficits during this stage. In mature ‘Marcona’ trees, complete irrigation cut-off during Stage II reduced yield by 25–45% [122]. Similarly, in ‘Tuono’, moderate deficit irrigation (RDI_50–60_) during Stage II reduced vegetative growth by 20–30% without significantly affecting yield, whereas very severe RDI_25_ reduced vegetative growth by 40–50% and yield by 10–15%. These reductions were associated with decreases in midday stem water potential to approximately −2.0 to −2.5 MPa and slight declines in kernel quality [136].

Likewise, in organically managed ‘Marcona’ orchards, the combination of mild RDI_80_ during Stages II and IV and severe RDI_40_ during Stage III reduced yield, particularly in young trees, while improving kernel quality through increased antioxidant activity and higher concentrations of phenolic compounds and sugars, as well as a greater proportion of beneficial fatty acids. These improvements in kernel quality were more pronounced in older trees [155].

Evidence synthesis: The available studies indicate that the response to Stage II water deficit is strongly dependent on deficit severity. Severe or complete irrigation cut-off can substantially reduce yield, as demonstrated in mature ‘Marcona’, whereas moderate deficits in ‘Tuono’ primarily affected vegetative growth without a significant yield reduction. Thus, the evidence that severe Stage II water stress increases yield risk is moderate, but the response to moderate Stage II RDI remains unresolved across cultivars and growing conditions. The reported 25–45% yield reduction under complete irrigation cut-off should therefore be interpreted as a response to severe stress rather than as a general estimate of the Stage II RDI yield penalty.

#### 8.2.3. RDI During Kernel Filling and Preharvest (Stage III)

Most studies have focused on RDI during Stage III, when crop water demand and kernel dry matter accumulation are at their highest. This period offers considerable potential for water savings because moderate water deficits can substantially reduce irrigation requirements while maintaining yield. Consequently, Stage III has become the principal target for RDI strategies, particularly in arid and semi-arid regions such as the Mediterranean basin, where irrigation demand reaches its peak during summer.

Nevertheless, almond tree responses to water deficits during kernel filling remain variable. Differences among studies can largely be attributed to variations in the timing, intensity, and duration of water stress, as well as cultivar, tree age, orchard management practices, and environmental conditions. Overall, the available evidence indicates that almond trees are relatively tolerant of mild-to-moderate water stress during Stage III, whereas severe or prolonged deficits can adversely affect vegetative growth, kernel development, and, ultimately, yield.

Consistent with this general pattern, several studies have shown that moderate RDI during kernel filling can maintain yield while substantially improving WUE. Although severe water stress may reduce hull split and leaf area [187], moderate water deficits generally preserve productivity with limited adverse effects [130]. In mature ‘Ferragnès’ trees, even the very severe RDI_20_ treatment maintained yields close to those of FI trees while reducing IWU by approximately 30%, thereby improving WUE [58]. Similarly, in ‘Cartagenera’, very severe preharvest RDI_20_ reduced leaf area through growth suppression and defoliation but resulted in only a nonsignificant reduction in yield of approximately 7%, while saving about 30% of irrigation water, provided that stem water potential remained above −2 MPa [125]. Over four consecutive years, the same strategy achieved approximately 28% water savings with only a 7% reduction in yield and reduced operating costs by about 10%, supporting the economic viability of RDI under semi-arid conditions [124]. Moreover, subsurface irrigation enhanced root development and drought tolerance by promoting deeper water extraction and improving water availability through capillary movement [113]. Comparable responses have been reported in other cultivars. In the autochthonous ‘Colorada’ cultivar, an RDI strategy consisting of RDI_70_ during Stages II and IV and RDI_30_ during Stage III reduced irrigation water use by up to 50% without affecting yield, whereas rainfed trees experienced substantial production losses. This stability was associated with osmotic adjustment and improved stomatal regulation. Likewise, in mature ‘Marta’ trees, RDI_50_ during kernel filling reduced water use while causing only minor reductions in kernel weight (approximately 3–7%), with overall fruit quality remaining largely unaffected and performance clearly superior to that of rainfed trees [109]. Similar findings were reported in ‘Guara’, where both LFDI and RDI_30_ during Stage III improved WUE and reduced irrigation water use by approximately 27–40% without significantly reducing nut yield relative to full irrigation [133]. Notably, LFDI increased nut yield by 16% compared with the FI control while saving nearly 170 mm of irrigation water, highlighting its potential as an efficient irrigation strategy under water-limited conditions [133].

Beyond maintaining productivity, moderate DI may also improve kernel quality. In mature ‘Tuono’ trees, RDI_50_ during Stage III maintained yield and had limited effects on physical fruit characteristics, while improving several aspects of kernel quality, including sugar-related traits, polyphenol content, and the sweetness-to-acidity ratio [151]. Similarly, long-term studies showed that moderate RDI_65_ maintained yields comparable to those under full irrigation, whereas more severe stress RDI_35_ reduced yield by up to 25%, although soil water reserves partly buffered the effects of reduced irrigation [149]. These findings support the use of moderate DI as a strategy for improving WUE while limiting yield penalties, whereas prolonged and severe water deficits can compromise physiological performance and productivity [153]. Moderate water deficits may also provide indirect benefits for pest management. In almond orchards, RDI during stage III reduced foliar damage (50–60% less) caused by the two-spotted spider mite (*Tetranychus urticae*) compared with fully irrigated treatments, suggesting that reduced water availability may limit excessive vegetative growth and thereby decrease pest damage [150]. Despite these benefits, a substantial body of evidence indicates that Stage III becomes increasingly sensitive to water stress when deficits are severe or prolonged. Although developing fruits are relatively weak sinks during pit hardening, severe water stress during kernel filling can disrupt assimilate partitioning and plant water relations, potentially resulting in kernel dehydration and the development of the “skinny kernel” disorder [52]. The detrimental effects of severe DI have been reported across cultivars and tree ages. In mature ‘Marcona’ orchards, complete irrigation cut-off during Stage III substantially reduced yield [127]. Likewise, preharvest water deprivation in mature ‘Nonpareil’ trees induced severe water stress (Ψpd ≈ −4.0 MPa), resulting in defoliation, reduced vegetative growth, lower kernel weight, and a marked reduction in full hull split [123]. Similarly, severe preharvest RDI in mature ‘Nonpareil ‘trees reduced yield and kernel weight, whereas a milder deficit of approximately 85% ETc had little effect [75]. In mature ‘Cartagenera’ trees, very severe RDI_20_ reduced leaf expansion and increased leaf abscission, resulting in yield losses despite substantial savings in irrigation water and improvements in WUE [113]. Although such severe deficit strategies may remain economically attractive under extreme water scarcity because of their substantial water savings, prolonged drought can also deplete carbohydrate reserves and inhibit floral induction, potentially reducing productivity in both the current and subsequent seasons [123]. Similar responses have been reported in young ‘Ferragnès’ trees, in which severe DI substantially reduced irrigation water use but, when prolonged, also decreased kernel dry weight, indicating cumulative limitations in carbohydrate accumulation [58]. Likewise, in young ‘Marta’ trees, RDI_40_ during Stage III reduced yield [69], whereas stronger deficits in mature ‘Ferragnès’ trees also resulted in substantial yield reductions despite considerable irrigation water savings [137].

Similarly, in mature ‘Nonpareil’ orchards, moderate DI maintained yield and kernel weight, whereas stronger deficits significantly reduced productivity under both RDI and SDI [141]. In mature ‘Guara’ trees, very severe RDI_15_ reduced seasonal evapotranspiration from 1088 to 699 mm, but yield simultaneously declined from 2508 to 1497 kg ha^−1^, indicating that improvements in irrigation efficiency were accompanied by substantial production losses, while evapotranspiration-based water productivity remained statistically unchanged [104,188].

Recent studies further reinforce the existence of a threshold beyond which water savings are offset by productivity losses. In ‘Marcona’, severe RDI_40_ during Stage III reduced yield, particularly in younger trees, although the combination of organic management and DI improved several kernel-quality traits, including antioxidant, phenolic, sugar, and fatty acid contents, particularly in older orchards [155]. Conversely, very severe RDI_35_ during kernel filling applied to young ‘Ferragnès’ trees over two consecutive years maintained relatively stable yield and physiological status, whereas rainfed and more severe SDI treatments substantially reduced chlorophyll content, photosynthesis, and plant water status [142]. Moreover, the effects of water deficit may be exacerbated by other abiotic stresses. In young potted ‘Guara’ trees, both saline irrigation (EC ≈ 3 dS m^−1^) and RDI_80_ reduced growth and physiological performance, while their combination caused the greatest reductions in g_s_, A_n_, and overall growth [147]. Collectively, these findings indicate that Stage III can tolerate moderate water deficits with limited effects on yield and, in some cases, improved kernel quality and WUE; however, severe or prolonged deficits can cross a physiological threshold beyond which reductions in kernel development, vegetative performance, and current and subsequent yield outweigh the benefits of irrigation saving.

Synthesis of moderate Stage III deficits: Across the cited studies, moderate RDI during kernel filling generally reduced irrigation requirements while maintaining yield close to that obtained under full irrigation in several cultivars. Reported irrigation-water savings were approximately 27–50% in several Stage III treatments, although the magnitude varied among cultivars and irrigation regimes [58,109,124,133]. These findings provide well-supported evidence that appropriately timed moderate Stage III RDI can reduce irrigation inputs with limited yield penalties under several Mediterranean and semi-arid conditions. However, these values represent the range reported among individual studies and should not be interpreted as a statistically pooled estimate.

Synthesis of severe Stage III deficits: The evidence becomes substantially more heterogeneous as deficit severity increases. Severe or prolonged Stage III deficits have reduced yield, kernel weight, leaf area, and vegetative performance in several cultivars, although some severe treatments maintained yield under particular cultivar and environmental conditions [58,69,104,123,137,141]. Therefore, the general relationship between increasing deficit severity and increasing risk of productivity loss is well-supported, whereas the precise deficit threshold beyond which yield losses become unacceptable remains unresolved and strongly context-dependent.

Yield–water trade-off: The available evidence indicates that increasing irrigation savings does not necessarily result in proportional yield losses under moderate, appropriately timed deficits. However, the most severe treatments demonstrate that a threshold can eventually be reached beyond which additional water savings are accompanied by substantial productivity losses. For example, in ‘Guara’, RDI15 reduced seasonal evapotranspiration from 1088 to 699 mm while yield declined from 2508 to 1497 kg ha^−1^ [104,188]. Thus, the existence of a yield–water trade-off under severe deficit is well-supported, but the agronomically or economically optimal threshold remains unresolved.

#### 8.2.4. RDI During Postharvest (Stage IV)

During the postharvest period, almond trees replenish carbohydrate reserves while floral buds differentiate before leaf fall and the onset of winter dormancy. These carbohydrate reserves are essential for supporting flowering and fruit set in the following season. Water stress from hull split through the postharvest period reduces carbohydrate accumulation, disrupts meristem transitions, and decreases both the number and quality of floral buds [55]. Water stress during dormancy may also delay bloom and alter flowering phenology [188]. Among the different phenological stages, the postharvest period is particularly sensitive to irrigation deficits. In ‘Nonpareil’ almond, postharvest water stress has a greater negative impact on subsequent yield than preharvest stress, substantially reducing flowering in the following season. Therefore, when water supplies are limited, irrigation should be prioritized during the postharvest period [123].

The response to postharvest water deficit varies according to cultivar, tree age, and stress intensity. In young ‘Nonpareil’ trees, late-season drought induced by early irrigation cut-off reduced plant water status, photosynthesis, and vegetative growth, while causing leaf rolling, premature defoliation, and incomplete recovery after rewatering [56]. In mature ‘Nonpareil’ trees, irrigation deficits during August and September, coinciding with floral bud development, caused only minor reductions in current-season yield but significantly decreased bloom and fruit set in the following year because of reduced carbohydrate availability and impaired meristem development [55]. Similarly, prolonged harvest-period RDI reduced CO_2_ assimilation by 14% under moderate stress (Ψms = −1.4 to −2.0 MPa) and by 30% under severe stress (Ψms = −2.0 to −2.6 MPa), while g_s_ decreased by 6% and 20%, respectively. Premature defoliation began at approximately −1.8 MPa, thereby reducing canopy light interception [110]. Although short-term postharvest irrigation cut-offs had little immediate effect on kernel weight or yield, prolonged water deficits increased spur mortality and reduced the renewal of fruiting wood, thereby decreasing long-term orchard productivity [112].

Several studies indicate that RDI can be used to reduce irrigation water use while maintaining almond productivity, although the effects depend on the timing and severity of the imposed water deficit [53].

In relatively young Nonpareil, mild postharvest RDI_85_ reduced irrigation water use by approximately 13% without affecting kernel weight, whereas severe stress during August and September significantly reduced bloom and fruit set [132]. Similarly, in adult ‘Nonpareil’ trees severe postharvest irrigation cut-offs reduced bloom by up to 52% and fruit set by as much as 94%, resulting in substantial yield losses [123].

By contrast, Mediterranean cultivars generally exhibit greater tolerance to postharvest water deficits. In mature ‘Marcona’ trees, repeated irrigation cut-offs during Stage IV over several years caused only minor yield reductions (0–15%), primarily affecting fruit growth rather than kernel size [122]. Similarly, adult ‘Colorada’ trees subjected to moderate RDI_70_ in Stage IV maintained yields comparable to those of fully irrigated trees, even under saline conditions, while reducing irrigation water use by approximately 50%; rainfed trees, however, produced significantly lower yields [127]. Comparable results have been reported for ‘Guara’, where moderate to severe postharvest RDI over two years only slightly reduced yield while improving WUE and economic returns [135]. Furthermore, long-term application of moderate postharvest RDI_65_ in ‘Guara’ maintained yields similar to those under FI over six years, with yield reductions generally limited to 0–5% [54].

Short synthesis: Yield response to RDI varies with phenological stage, deficit severity, cultivar, and environmental conditions. Stage III provides the strongest evidence for water savings, with moderate RDI reducing irrigation requirements by approximately 27–50% without significant yield losses in several cultivars. However, severe deficits can substantially reduce yield, as observed in ‘Guara’, where RDI15 resulted in an approximately 40% reduction in yield. Evidence for Stage I remains insufficient to define a consistent yield-response threshold. Severe water stress during Stage II can reduce yield by 25–45%, whereas postharvest irrigation (Stage IV) may also influence subsequent-season reproductive performance. Overall, moderate and appropriately timed RDI can reduce water use with limited yield penalties, but irrigation thresholds remain cultivar- and environment-specific.

### 8.3. Sustained Deficit Irrigation

SDI, in which a controlled water deficit is maintained throughout the growing season, has been widely evaluated in almond orchards. Its effects depend on cultivar, tree age, and the severity and duration of water stress.

Early studies demonstrated a nonlinear relationship between irrigation level and almond productivity. In mature ‘Ruby’ trees irrigated at SDI_50_, SDI_75_, SDI_100_, SDI_125_, SDI_150_, and SDI_175_ over three years, tree growth, plant water status, and nut yield increased with increasing irrigation up to SDI_125_, with no additional benefits from higher irrigation levels (SDI_150_ and SDI_175_) [110]. This finding indicates that irrigation beyond the level required to avoid water limitation does not necessarily translate into additional yield benefits. Similarly, in mature ‘Ferragnès’ trees monitored for three years, SDI_70_ (30% deficit) reduced Ψleaf, g_s_, vegetative growth, and yield, whereas over-irrigation at SDI_130_ stimulated vegetative growth without significantly increasing yield.

Subsequent studies further demonstrated that the magnitude of yield reduction increases as the severity of SDI increases. In mature ‘Nonpareil’ trees, SDI_85_, SDI_70_, and SDI_55_ decreased yield by approximately 5%, 15%, and 30%, respectively, while kernel weight declined by 5–15% as water stress intensified. These yield reductions were mainly associated with smaller kernels rather than reductions in nut number [71]. Similarly, four- to six-year-old ‘Ferragnès’ trees subjected to SDI_70_ for four years produced lower yields and smaller kernels [58]. Comparable responses were observed in young ‘Marta’ trees, where SDI_80_ caused only minor reductions in productivity, whereas SDI_60_ significantly reduced yield, trunk growth, and physiological performance [127].

A six-year study during orchard establishment further demonstrated progressive reductions in vegetative growth and yield with increasing water deficit. However, SDI_60_ increased IWP by 92.5%, reduced IWU by approximately 63%, and limited yield reduction to 29%, indicating its potential as a water-saving strategy [69]. Similarly, increasing water deficits progressively reduced physiological performance and yield, although moderate DI maintained relatively high WUE, whereas severe SDI_50_ resulted in substantial productivity losses [129]. The negative effects of water deficit became more pronounced under severe and prolonged stress. In mature ‘Ferragnès’ trees, SDI_50_ reduced yield by 23% while saving approximately 50% of irrigation water, whereas the RDI treatment with a severe deficit during stage III reduced yield by 31% with similar water savings [137]. Long-term effects were also observed in mature ‘Guara’ orchards, where SDI_25_ reduced kernel size and increased kernel defects, while rainfed conditions resulted in up to 92% tree mortality and persistent yield reductions even after irrigation was resumed [151]. Similarly, extreme drought from mid-May to November in mature ‘Vairo’ orchards resulted in approximately 92% yield loss and 42% tree mortality under rainfed conditions [157].

In contrast, moderate SDI can maintain productivity in cultivars with greater tolerance to water deficits. In mature ‘Guara’ orchards, SDI_65_ maintained yields within 0–5% of those obtained under FI over six consecutive years [149].

Beyond yield responses, moderate DI may also influence kernel quality. SDI_75_ increased the proportion of monounsaturated fatty acids and affected tocopherol concentrations in almond kernels, although the magnitude and direction of these responses depended on the severity of water deficit [138]. Similarly, SDI modified soluble sugars, phenolic compounds, and volatile profiles in ‘Marta’, ‘Guara’, and ‘Lauranne’, with the magnitude of these responses varying among cultivars [97].

In addition to optimizing the amount of water applied, irrigation efficiency can be improved through appropriate water application methods. Based on HYDRUS-2D simulations, pulsed drip irrigation supplying 65% of ETc was shown to improve soil moisture distribution and increase root water-uptake efficiency to 68% in ‘Nonpareil’, ‘Carmel’, and ‘Ne Plus Ultra’, compared with 54–55% under fFI. Consequently, irrigation water productivity was increased by approximately 37%, while approximately 35% of irrigation water was saved, despite an 8% reduction in yield [118]. In contrast, when FI requirements were met, the maximum three-year average yield of 2508.4 kg ha^−1^ was obtained in the almond cultivar ‘Guara’. As the proportion of full irrigation requirements was reduced to 66.9%, 69.7%, and 43.2%, average yields decreased to 2147.5, 2038.2, and 1496.9 kg ha^−1^, respectively [100].

Evidence synthesis: Across the reviewed studies, increasing SDI severity generally increased the risk of reductions in vegetative growth, kernel size, and yield, although the magnitude of the response varied substantially among cultivars and environments. This relationship is well-supported by multi-year studies in ‘Nonpareil’, ‘Ferragnès’, ‘Marta’, and ‘Guara’ [58,69,71,127,149]. Moderate SDI can maintain relatively high productivity in some cultivars, such as ‘Guara’, where SDI65 maintained yield within approximately 0–5% of FI over six years [149]. However, this result is cultivar-specific. Therefore, the evidence that increasing deficit severity increases yield risk is well-supported, whereas the existence of a universal SDI threshold for maintaining yield remains unresolved.

### 8.4. Partial Root-Zone Drying (PRD)

Partial root-zone drying (PRD), originally developed and evaluated in Australian vineyards, is an irrigation strategy in which only part of the root-zone is irrigated at a given time while the remaining portion is allowed to dry, with the irrigated and dry portions subsequently alternated. The approach was developed to reduce vegetative vigour and transpiration while maintaining crop productivity and improving WUE [73]. Although its application in almond orchards has been more limited than that of RDI, studies have demonstrated that PRD can reduce irrigation water use while maintaining relatively high productivity and WUE. In mature ‘Marta’ trees, PRD_50_ reduced irrigation water use without significantly affecting yield or several quality attributes, whereas rainfed trees generally showed lower productivity and poorer fruit quality [131]. These results indicate that moderate PRD can provide substantial water savings without necessarily causing major reductions in almond yield or kernel quality. However, the response to PRD depends strongly on the severity of water restriction. In mature ‘Marta’ trees, PRD_70_, PRD_50_, and PRD_30_ were evaluated over the growing season, with progressively greater water deficits resulting in greater reductions in kernel dry weight. The most severe treatments, particularly PRD_30_ and PRD_50_, reduced final kernel dry weight, whereas PRD_70_ caused comparatively minor reductions and showed a response similar to RDI during kernel filling [130]. Longer-term observations further demonstrated that decreasing water application under PRD progressively reduced tree growth and yield, although water productivity increased markedly as irrigation decreased. WP reached 123% of that under FI in PRD_30_, while all DI treatments negatively affected trunk growth. Moreover, when PRD_70_ and RDI supplied similar annual amounts of water, tree performance was broadly comparable between the two strategies [109]. Thus, moderate PRD can improve WUE with limited effects on productivity, whereas severe or prolonged deficits may reduce kernel weight, yield, and vegetative growth. Overall, both PRD and RDI can reduce irrigation water use, but their effectiveness depends on the severity and timing of water restriction.

Evidence synthesis: The available evidence indicates that moderate PRD can reduce irrigation inputs while maintaining relatively high productivity in some almond cultivars, and that increasing water restriction generally increases the risk of reductions in kernel weight, tree growth, and yield [109,130,131]. The evidence for improved water productivity under PRD is therefore moderate. However, a consistent yield or agronomic advantage of PRD over RDI remains unresolved. Comparisons under similar water inputs suggest that deficit severity and timing may explain more of the variation in yield than the irrigation configuration itself [109,130,189]. This conclusion is nevertheless constrained by the relatively limited number of almond PRD studies and their concentration in particular cultivars and environments.

### 8.5. Comparative Evidence: RDI Versus SDI and PRD

Across the literature, the effects of SDI and RDI on almond yield depend strongly on the severity, timing, and duration of water deficits, as well as on cultivar and tree age. Early comparisons showed that severe RDI imposed during kernel filling could substantially reduce yield, whereas continuous moderate SDI could maintain relatively high productivity while considerably reducing irrigation inputs [120]. Similarly, long-term experiments in ‘Ferragnès’ demonstrated that SDI reduced yield and kernel size as deficit severity increased [58].

In mature ‘Nonpareil’ trees, comparisons between SDI and RDI showed that moderate deficits could maintain yields relatively close to those obtained under FI, whereas stronger deficits resulted in progressively greater yield reductions. In particular, irrigation at approximately 85% of the fully irrigated treatment had little effect on yield or individual kernel weight, while irrigation at 70% or less generally reduced kernel yield, although the response varied among seasons and irrigation strategies [71]. More recent field experiments likewise indicate that moderate SDI can maintain acceptable production, but cultivar responses differ. In young ‘Guara’, ‘Marta’, and ‘Lauranne’ trees, SDI_75_ maintained relatively high productivity, although the magnitude of the response varied among cultivars and environmental conditions [115].

The response to DI is particularly important during orchard establishment, when water stress can affect both vegetative development and reproductive performance. In young almond trees, SDI restricted vegetative growth as deficit severity increased, whereas appropriately timed RDI could reduce water use while limiting excessive vegetative development and maintaining reproductive performance [127] A six-year study further demonstrated that increasing water deficits progressively reduced vegetative growth and yield. However, moderate to severe SDI_60–30_ increased IWP by 92.5%, reduced irrigation water use by approximately 63%, and reduced yield by 29%. In comparison, RDI_40_ applied during kernel filling and moderate SDI_75–60_ achieved relatively highwater productivity, while RDI improved fruiting efficiency and limited excessive vegetative growth. Thus, severe SDI was more appropriate under extreme water scarcity, whereas RDI_40_ and moderate SDI were more suitable under moderate water limitations [69]. From an economic perspective, RDI_40_ and SDI_75–60_ also showed greater feasibility under moderate water scarcity, whereas the economic performance of full irrigation and severe SDI depended on the production and water-saving conditions considered [134]. Overall, these results indicate that differences in yield between SDI and RDI are often relatively small when comparable amounts of water are applied, with the response depending more strongly on deficit severity and timing than on irrigation strategy alone [69].

In mature ‘Ferragnès’ orchards, the timing and intensity of water restriction were also critical. Very severe RDI_20_ imposed during Stage III provided a favourable compromise between yield preservation and water savings, whereas SDI_50_ imposed throughout the season caused greater cumulative water stress and a larger yield reduction. Although fully irrigated trees produced the highest yield, WUE did not differ substantially between the SDI and RDI treatments, indicating that both approaches can be effective where irrigation water is limited [137]. Similarly, in mature ‘Nonpareil’ orchards, moderate deficit irrigation maintained yields relatively close to those under full irrigation, whereas stronger deficits reduced production. The relative performance of SDI and RDI varied among seasons, indicating that climatic conditions and seasonal water availability can influence the response to each strategy [141]. In young ‘Ferragnès’ trees, severe RDI_35_ during kernel filling maintained yield and physiological performance over two seasons, whereas rainfed conditions and severe SDI_35_ resulted in yield reductions and pronounced phenological, morphological, and physiological adjustments, including early summer leaf shedding as a mechanism for reducing water loss [142]. Similarly, long-term experiments in ‘Guara’ showed that LFDI maintained yields comparable to those of fully irrigated trees, whereas RDI_50_ applied during kernel filling resulted in significantly lower yields [77].

Comparisons between PRD and RDI indicate that almond yield responses are driven mainly by deficit severity and timing rather than irrigation strategy. Under similar soil moisture conditions, PRD_50_ improved leaf-level water-use efficiency and afternoon plant water status, although effects on stomatal regulation and midday water status were inconsistent [62]. Similarly, differences in kernel yield and quality were primarily related to deficit severity rather than irrigation method [109,130], consistent with broader evidence showing no consistent yield advantage of PRD over RDI [189]. Overall, RDI provides greater control over stress timing, SDI enables consistent seasonal deficits, and PRD may improve plant water relations through heterogeneous soil drying, although its advantages are not consistently observed under field conditions.

The reviewed studies provide a basis for comparing regulated deficit irrigation (RDI), sustained deficit irrigation (SDI), and partial root-zone drying (PRD) in terms of their effects on almond yield under different levels and timings of water restriction. Across the reviewed studies, deficit severity and timing generally appear to have a stronger influence on almond yield than the irrigation strategy itself when comparable amounts of water are supplied. RDI provides greater control over the timing of water stress, SDI imposes a more continuous seasonal deficit, and PRD creates spatially heterogeneous soil drying. However, the available evidence does not demonstrate a consistent yield advantage of one strategy over another under comparable water inputs. The evidence that deficit severity and timing are major determinants of yield response is well-supported, whereas the broader conclusion that irrigation strategy per se has a smaller effect than water amount and timing is moderately supported because direct comparisons remain limited. In particular, the comparative advantage of PRD over RDI remains unresolved, and cultivar-specific responses prevent identification of a universal optimum deficit level.

### 8.6. Effects of DI on Long-Term Yield and Productivity

Water stress induces a range of physiological and agronomic responses, with their magnitude depending on the severity, duration, and timing of the stress [190]. In almond, yield is determined by several components, including flower production, fruit set, and kernel weight, with the abundance of flowers and the maintenance of productive fruiting structures being particularly important determinants of yield [151,191]. Long-term productivity therefore depends strongly on the maintenance and renewal of fruiting spurs, which are influenced by the annual growth and survival of existing spurs as well as the formation of new shoots that develop into future fruiting sites. Spurs with greater leaf area have greater survival and flowering potential in subsequent seasons, whereas excessive fruiting can reduce spur leaf area and weaken the structures supporting future production, ultimately increasing spur mortality and reducing subsequent yield [192,193,194]. These relationships highlight the importance of maintaining sufficient vegetative growth and healthy fruiting spurs for sustaining almond productivity over successive seasons.

The impact of water stress on long-term productivity depends strongly on its timing. Short-term deficits imposed during specific developmental stages may primarily reduce kernel growth, whereas prolonged or poorly timed stress can affect reproductive development and the renewal of fruiting structures. In particular, water deficits imposed before harvest can have relatively limited effects on subsequent reproductive performance, whereas severe postharvest water deprivation can reduce flowering and fruit set in the following season [123]. Prolonged water stress may also reduce photosynthetic activity and carbohydrate accumulation, thereby limiting the resources available for subsequent growth and reproduction [58].

Repeated or prolonged water deficits can therefore produce cumulative effects even when current-season yield is initially maintained. In mature ‘Nonpareil’ trees, repeated irrigation cut-offs around harvest progressively reduced nut load and yield over successive years, with the effects associated with changes in fruiting-spur dynamics and the renewal of productive structures [112]. Postharvest water deprivation can also delay physiological recovery following stress, while prolonged deficits may reduce the accumulation of carbohydrate reserves required to support subsequent reproductive development [112]. Conversely, moderate RDI can reduce irrigation water use without necessarily causing significant reductions in yield over several years, although kernel weight may decline slightly under sustained stress [132]. These findings indicate that the absence of an immediate yield penalty does not necessarily imply that prolonged water stress is without consequences for long-term tree productivity.

Evidence from other cultivars further demonstrates the importance of stress duration and intensity. In mature ‘Ferragnès’ trees, a very severe RDI_20_ treatment imposed during kernel filling maintained yield during the first two years but subsequently reduced kernel growth and yield, indicating a cumulative response to repeated seasonal water deficits [58]. In mature ‘Cartagenera’ trees, repeated RDI during kernel filling resulted in approximately 7% lower production while substantially reducing irrigation water use, demonstrating that moderate seasonal deficits can maintain relatively high productivity over several years [113]. Likewise, a six-year study in ‘Marta’ showed that increasing SDI severity progressively reduced tree growth and yield, with the effects particularly evident when severe water stress was imposed during kernel filling [69].

However, carefully timed DI can maintain long-term productivity while improving WUE. LFDI applied during kernel filling maintained yields comparable to FI over the long term in ‘Guara’, demonstrating that irrigation timing and the control of plant water status can be more important than simply reducing the total amount of water applied [77]. Nevertheless, the sustainability of deficit irrigation depends on avoiding prolonged or excessive stress. Recent work has shown that poorly managed DI can reduce photosynthetic productivity and compromise yield, emphasizing the need to match irrigation supply with tree water requirements and to monitor plant water status when implementing water-saving strategies [120].

Repeated exposure to moderate water stress may also induce physiological acclimation that improves drought tolerance. Such acclimation can involve osmotic adjustment, solute accumulation, and changes in tissue properties that help maintain leaf turgor and physiological activity under declining water availability [56,195]. More generally, controlled exposure to water stress can induce physiological adjustments that reduce the effects of subsequent stress in woody plants [195].

Water stress can also modify root–shoot allocation and promote greater investment in root growth under some conditions, thereby improving soil exploration and water uptake. Experimental studies have shown that water stress can maintain or stimulate root growth relative to shoot growth, particularly under moderate stress, although the response depends on species, stress intensity, and developmental stage [196]. Similar changes in root–shoot allocation and hormonal regulation have been widely recognized as components of plant adaptation to water limitation [84,190,197]. In young ‘Nonpareil’ almond trees, drought responses are primarily associated with reductions in stomatal conductance, photosynthetic activity, and vegetative growth, while severe or prolonged late-season stress can impair recovery following rewatering [56].

Overall, the long-term effects of DI depend not only on the amount of water withheld but also on the timing, duration, and recurrence of the stress. Moderate and well-timed deficits can maintain productivity while improving water-use efficiency, whereas severe or repeated deficits may progressively impair kernel growth, carbohydrate accumulation, spur renewal, and reproductive development. Consequently, sustainable deficit-irrigation strategies should aim to maintain sufficient tree water status during the developmental stages most critical for yield formation and the maintenance of future fruiting structures.

### 8.7. Effects of DI on Tree Age

Almond responses to DI also vary considerably with tree age. Young trees are particularly sensitive to water deficits because they are undergoing rapid vegetative development and have a relatively limited root system and smaller structural reserves. Consequently, water stress during the early stages of tree development can substantially restrict vegetative growth and trunk development [106,107]. In young almond trees, growth-based indicators such as trunk growth rate are particularly responsive to changes in plant water status, demonstrating the sensitivity of developing trees to water limitation [107].

Because tree size and canopy development are important determinants of almond productivity, restrictions in vegetative growth during orchard establishment have been identified as important determinants of almond yield. Irrigation management during the early years can therefore influence the productive potential of the mature orchard [109,110,112,198]. Accordingly, the early application of severe or prolonged RDI or SDI may reduce future productivity by limiting canopy development and the formation of productive fruiting structures [71,113]. This sensitivity is particularly important during orchard establishment. Although almond is generally considered a drought-tolerant species, water availability during the establishment phase remains critical for rapid canopy development and the attainment of full productive potential. Severe water deficits or prolonged irrigation suspension during this period can markedly restrict vegetative growth and delay orchard development [56]. Therefore, DI in young orchards should be applied cautiously, with particular attention to the severity and timing of the imposed deficit.

Evidence from mature ‘Ferragnès’ trees shows that sustained or severe deficits can reduce growth and yield, but these responses should not be extrapolated directly to young orchards because developing trees have different growth requirements and resource allocation patterns [58]. As trees mature, their larger root systems and more developed canopies allow them to access a greater soil-water volume and tolerate moderate water deficits more effectively than young trees. However, mature trees are not insensitive to water stress, and prolonged or severe deficits can still reduce physiological performance, kernel growth, and productivity. For example, mature ‘Marta’ trees subjected to contrasting DI strategies showed reductions in trunk growth under all deficit treatments, with the magnitude of the response increasing as the amount of irrigation water decreased [130]. Thus, the greater tolerance of mature trees should be interpreted as a relative difference in their response to water limitation rather than as an absence of sensitivity.

Recent evidence further highlights the importance of tree age and production system. In the ‘Marcona’ cultivar, organic management reduced productivity particularly in young, two-year-old trees, mainly through lower vegetative growth. Moreover, nut quality in young trees was influenced more strongly by the production system than by irrigation treatment, whereas irrigation effects became more evident in mature, seven-year-old trees [155]. These findings indicate that the relative importance of irrigation and other orchard-management factors changes as trees progress from establishment to full production.

Overall, young almond trees are generally more vulnerable to severe water deficits because water availability directly affects canopy establishment, vegetative growth, and the development of the structural framework required for future fruit production. In mature orchards, moderate DI can be used more flexibly because established trees have greater root exploration and structural development; nevertheless, severe or prolonged stress can still compromise yield and tree performance. Consequently, irrigation strategies should be adjusted according to tree age, with greater emphasis on maintaining adequate water availability during orchard establishment and greater flexibility for carefully managed deficit irrigation once the canopy and root system are fully developed.

### 8.8. Effects of DI on Different Cultivars

Cultivar responses to DI and drought vary considerably, reflecting differences in physiological regulation, water-use strategies, hydraulic characteristics, and reproductive responses. This genotypic variability is particularly important in water-limited environments, where cultivar selection can contribute to maintaining productivity and improving resilience to increasing water scarcity. Consequently, the suitability of DI depends not only on the amount and timing of water supplied but also on the intrinsic drought response of the cultivar [199]. In a study of 23 almond cultivars grown in Apulia, southern Italy, differences in response to water stress were also detected using the incidence of hull-tight nuts as an indicator of stress sensitivity, highlighting the importance of cultivar-specific adaptation to semi-arid conditions [200].

Considerable cultivar differences have been reported among young almond trees subjected to SDI. In young ‘Guara’, ‘Marta’, and ‘Lauranne’ trees, SDI_75_ and SDI_65_ produced different effects on yield, plant water status, and physiological performance, demonstrating that the response to a given irrigation deficit is cultivar dependent [116]. In this experiment, ‘Lauranne’ generally showed a relatively stable response to irrigation restriction, whereas ‘Guara’ and ‘Marta’ exhibited greater changes in physiological and yield-related traits under deficit conditions. However, the magnitude of the response varied according to the parameter considered, and therefore these cultivars should not be classified simply as tolerant or sensitive on the basis of a single trait [116]. Complementary work under young-tree conditions showed that biostimulant application could modify the response of ‘Guara’, ‘Marta’, and ‘Lauranne’ to SDI_75_, indicating that management practices can interact with cultivar-specific drought responses [114].

Cultivar differences are also evident in mature trees and in fruit-quality responses. In mature ‘Marta’, ‘Guara’, and ‘Lauranne’ trees subjected to FI, over-irrigation at 150% ETc, or RDI_65_ during kernel filling, ‘Marta’ was identified as the cultivar most sensitive to water restriction and showed the greatest response to moderate RDI. Nevertheless, irrigation treatment produced relatively small changes in overall morphological and physicochemical quality attributes, although moderate RDI modified selected color, sugar, organic-acid, and unsaturated-fatty-acid parameters [117]. These findings indicate that cultivar-specific sensitivity to water deficit does not necessarily translate into large changes in overall nut quality. Similarly, under SDI, ‘Guara’ showed greater reductions in yield than ‘Marta’ and ‘Lauranne’, whereas the latter two cultivars showed comparatively stable yield responses under moderate water restriction [115].

Further evidence of genotypic variability comes from mature cultivars subjected to severe or moderate RDI. Under severe RDI_35_ during kernel filling, ‘Constantí’ maintained yield comparable to FI, whereas ‘Vairo’ showed a reduction in cumulative yield, indicating contrasting cultivar responses to the same level and timing of water restriction [18]. Similarly, a recent comparison of ‘Guara’, ‘Masbovera’, ‘Glorieta’, ‘Francolí’, ‘Lauranne’, and ‘Ferragnès’ under RDI_40_ and rainfed conditions demonstrated substantial differences in drought-response traits and productivity, confirming that drought tolerance is strongly genotype dependent [157]. These differences emphasize that irrigation recommendations should be developed with consideration of cultivar-specific responses rather than applied uniformly across almond orchards.

Differences among cultivars are also associated with contrasting strategies of plant-water regulation. ‘Soleta’ and ‘Isabelona’ displayed characteristics associated with a more anisohydric behaviour, whereas ‘Avijor’ showed a more conservative, isohydric response to declining water availability. The three cultivars also differed in hydraulic vulnerability, with ‘Soleta’ and ‘Isabelona’ exhibiting lower water potentials at 50% loss of hydraulic conductivity than ‘Avijor’ [95]. These contrasting hydraulic strategies can influence the capacity of cultivars to tolerate fluctuations in plant water status, although a greater capacity to maintain gas exchange under drought may also be associated with greater water use and hydraulic risk under severe stress. Therefore, anisohydric behaviour should not automatically be interpreted as greater suitability under extreme water scarcity.

In one-year-old almond trees grown under well-watered conditions, ‘Guara’ and ‘Soleta’ showed comparatively high growth and WUE, whereas ‘Penta’ exhibited weaker performance. However, ‘Soleta’ also used approximately 25% more water, indicating that high growth and WUE do not necessarily correspond to the lowest water requirement [17]. This distinction is particularly relevant when selecting cultivars for environments where irrigation water is severely limited.

Stomatal regulation is another important component of cultivar-specific drought responses. Cultivars differ in the degree to which stomatal conductance is reduced as plant water status declines, thereby affecting the balance between carbon assimilation and water conservation. The contrasting hydraulic and stomatal behaviours observed among almond cultivars support the distinction between more anisohydric and more isohydric strategies [95,153,201]. Under field conditions, Ψstem, particularly when interpreted together with atmospheric VPD, can therefore provide a useful basis for comparing cultivar water status and improving irrigation scheduling [31].

Other field studies have likewise demonstrated substantial differences in drought tolerance among almond cultivars. Under water restriction, ‘Arrubia’ and ‘Texas’ exhibited greater physiological acclimation and maintained higher yields than ‘Tuono’, whereas ‘Cossu’ showed a lower capacity to cope with combined water and heat stress. These differences were associated with cultivar-specific stomatal regulation, anatomical traits, and morphological characteristics affecting leaf hydraulic conductance and gas exchange, highlighting the importance of genetic background in determining physiological acclimation and yield stability under water limitation [168,202].

Earlier studies similarly demonstrated contrasting responses among cultivars, with ‘Lauranne’ and ‘Francolí’ showing relatively low sensitivity to irrigation restriction in some Mediterranean environments [163].

Cultivar-specific responses can also occur under stage-specific irrigation. In ‘Butte’, ‘Aldrich’, and ‘Nonpareil’, irrigation restriction during hull split produced different effects on kernel and shell characteristics, with ‘Aldrich’ showing increased kernel thickness under RDI_75_, whereas ‘Butte’ exhibited changes in the proportion of sealed shells under RDI_75_ and RDI_50_ [152]. Such responses further demonstrate that the effects of DI may extend beyond total yield to individual components of nut development and quality.

Morphological and anatomical characteristics provide additional mechanisms underlying cultivar differences in drought adaptation. Traditional cultivars such as ‘Bonita’, ‘Casanova’, ‘Parada’, ‘Pegarinhos’, and ‘Verdeal’ have been reported to possess leaf anatomical characteristics associated with greater drought resistance than commercial cultivars such as ‘Ferragnès’ and ‘Glorieta’. In particular, the greater sensitivity of ‘Ferragnès’ was associated with a thinner cuticle, a lower palisade-to-spongy-mesophyll ratio, and greater xylem vulnerability [203]. Differences in gas exchange and water-use efficiency have also been reported between cultivars; for example, ‘Masbovera’ showed higher photosynthetic performance, WUE, and stomatal regulation than ‘Lauranne’ under water stress [204]. Likewise, ‘Garrigues’ showed greater capacity for drought adjustment than ‘Ramillete’, particularly through stronger stomatal control and osmotic adjustment during water stress [166].

Biochemical responses provide further evidence of genotypic differences in drought adaptation. Under severe water stress, ‘Supernova’ and ‘Azar’ maintained comparatively high photosynthetic performance and WUE, whereas ‘Marcona’ and ‘Sahand’ were more sensitive. ‘Supernova’ and ‘Azar’ also maintained relatively high leaf water status under severe stress, indicating greater capacity for osmotic adjustment and drought acclimation [205]. More recently, ‘Garnem’, ‘Shahrood 8’, and ‘Shahrood 12’ exhibited high relative water content, antioxidant activity, and phenolic accumulation under drought, indicating stronger physiological and biochemical protection against water stress [206]. Similarly, ‘A-7-100’, ‘A-100-13’, and ‘A-100-8’ genotypes showed enhanced drought tolerance associated with greater proline accumulation, higher relative water content, and improved Fv/Fm values [207].

Root characteristics and genetic background also contribute to drought resilience. Wild almond relatives, including ‘*Prunus webbii*’ and ‘*Amygdalus webbii*’, have been reported to exhibit greater drought tolerance than cultivated almond, partly through differences in leaf area, stomatal characteristics, and other structural traits [207,208]. Stomatal traits further contribute to this variability, with cultivated almond showing different responses in stomatal density compared with ‘*A. webbii*’ under water stress [208]. Root-system architecture is also important because deeper and more extensive roots can increase access to soil water and improve recovery following periods of stress [209]. In addition, differences in recovery after re-irrigation indicate that cultivars can differ in their hydraulic adjustment and capacity to recover from water stress [210].

Cultivar differences are not restricted to drought. Similar genotype-dependent responses occur under salinity stress, with ‘Guara’ and ‘Penta’ maintaining better physiological performance and ion balance than the more sensitive ‘Avijor’, while ‘Vialfas’ showed an intermediate response [211]. Finally, irrigation technology can interact with cultivar characteristics. For example, pulsed drip irrigation improved root water-uptake efficiency and irrigation water productivity in ‘Nonpareil’, ‘Carmel’, and ‘Ne Plus Ultra’, demonstrating that irrigation-system design can complement cultivar selection to improve water use under limited water availability [118].

Overall, the available evidence demonstrates that almond cultivars differ substantially in their physiological, anatomical, biochemical, hydraulic, and agronomic responses to water limitation. However, drought tolerance is not a single trait: a cultivar may maintain yield under moderate deficit irrigation while exhibiting high water use, or maintain plant water status through strong stomatal closure at the expense of carbon assimilation. Therefore, cultivar selection for water-limited environments should consider yield stability, water-use efficiency, hydraulic safety, physiological acclimation, recovery capacity, and the specific timing and severity of the irrigation deficit rather than relying on a single indicator of drought tolerance.

### 8.9. Effects of DI on Kernel Composition and Quality

Water availability is an important determinant of almond kernel composition, nutritional value, and the development of hydroSOStainable (hydroSOS) almonds produced under controlled water stress. Consequently, research on DI has examined its effects on kernel morphology and proximate composition, as well as on nutritionally and functionally important compounds, including oil, fatty acids, sugars, organic acids, minerals, phenolics, antioxidant compounds, and volatile metabolites [22,212]. HydroSOStainable almonds are produced through controlled deficit irrigation and may exhibit changes in functional and sensory characteristics while reducing irrigation water use.

Among the cultivars evaluated, irrigation effects on kernel quality were strongly dependent on both irrigation level and genotype. In mature ‘Marta’, ‘Guara’, and ‘Lauranne’ trees, over-irrigation at 150% ETc generally increased kernel size and weight but did not consistently improve quality. In contrast, moderate RDI_65_ during kernel filling enhanced several textural and sensory attributes, including kernel hardness and fracturability, while also modifying skin color and selected sugars, organic acids, and fatty acids. The response was particularly evident in ‘Marta’, which showed greater sensitivity to irrigation level, whereas ‘Guara’ and ‘Lauranne’ exhibited comparatively greater stability under water limitation. ‘Guara’ produced the largest and hardest kernels, while ‘Lauranne’ also showed high kernel hardness and ‘Marta’ was characterized by darker skin coloration [116]. These findings demonstrate that moderate water restriction can modify kernel quality without necessarily causing an overall deterioration in marketability.

Mineral composition showed a more conservative response to irrigation. In ‘Guara’, ‘Marta’, and ‘Lauranne’, potassium, calcium, magnesium, and manganese concentrations were generally not substantially altered by irrigation treatment, although small treatment effects were detected for some elements. Mean potassium concentration was approximately 508 mg 100 g^−1^, exceeding the nutritional threshold specified in the European nutritional-labelling framework, while calcium concentrations were higher than those previously reported for ‘Vairo’ [22,116]. Thus, although irrigation can modify some nutritional components, mineral composition appears comparatively stable across moderate irrigation regimes.

In ‘Vairo’, the application of RDI and SDI during kernel filling produced relatively limited effects on most major physicochemical characteristics. Citric acid, sucrose, antioxidant activity, and total phenolic content were generally not strongly affected by the irrigation treatments, although stressed treatments modified malic acid and glucose and moderate RDI increased the abundance of some volatile compounds. Overall, yield and most quality parameters were maintained under DI, with moderate RDI producing some favourable biochemical responses [22].

The effects of water stress on the functional quality of ‘Vairo’ almonds were more evident when individual phenolic compounds were considered. Moderate RDI increased total phenolic content in the almond skin by approximately 9.8% and enhanced proanthocyanidin accumulation and antioxidant activity. These changes provide a biochemical basis for the hydroSOS concept, in which controlled water stress can increase selected bioactive compounds while simultaneously reducing irrigation requirements [144].

Comparable cultivar-dependent responses have been observed under sustained deficit irrigation. In ‘Guara’, ‘Marta’, and ‘Lauranne’, SDI modified several biochemical and aroma-related characteristics, including sugars, phenolic compounds, and volatile metabolites. Sugar concentrations increased under DI, with the magnitude of the response varying among cultivars, while total phenolic content increased particularly in ‘Guara’ and ‘Marta’. SDI also modified the volatile profile, demonstrating that irrigation management can influence not only nutritional composition but also compounds associated with almond aroma and sensory quality [97].

Irrigation also affected the lipid fraction of almond kernels. In ‘Nonpareil’, moderate SDI_85_ increased the oleic-to-linoleic acid ratio, whereas the more severe SDI_50_ treatment reduced this ratio; tocopherol concentration, however, remained relatively stable among irrigation treatments [138]. These findings suggest that moderate water restriction may favour a more desirable fatty-acid profile without necessarily compromising antioxidant protection provided by tocopherols. More broadly, studies of ‘Ferragnès’ and ‘Texas’ have shown that irrigation level can influence maturation and oil composition, with irrigation affecting oleic acid and sucrose concentrations, whereas delayed harvest increased kernel dry matter, oil, and sugar contents but could negatively affect some aspects of oil quality [98].

The potential of DI to produce hydroSOS almonds is therefore associated not simply with maintaining kernel quality under reduced irrigation, but with the possibility of selectively enhancing certain functional and sensory characteristics. Moderate RDI has been particularly promising because it can maintain yield while modifying phenolic compounds, fatty-acid composition, sugars, organic acids, and aroma-related metabolites. However, these responses are cultivar- and trait-dependent, and severe or prolonged water stress should not be assumed to improve quality uniformly.

Finally, water management can influence quality beyond harvest through its effects on storage behaviour. In ‘Marta’, almonds produced under rainfed conditions showed smaller kernels and differences in sugar concentration, phenolic retention, antioxidant activity, and sensory attributes during storage compared with irrigated treatments. By contrast, almonds produced under FI, PRD, and RDI showed broadly comparable quality and storage stability, indicating that moderate water-saving strategies can reduce irrigation inputs without necessarily compromising postharvest quality [130]. These findings further support the potential of appropriately managed DI for producing almonds with stable nutritional and sensory characteristics while improving water-use efficiency.

### 8.10. Economic Analyses of DI (Overview)

Economic analyses of DI in almond orchards have used approaches including production-function optimization, discounted cash-flow analysis, and cost–benefit assessment to evaluate the trade-offs between water savings, yield, production costs, and profitability. These analyses are particularly relevant in semi-arid regions, where irrigation water is limited and its allocation among agricultural uses has a direct economic value. One of the early demonstrations of the economic potential of RDI was provided by Romero et al. (2005) [126]. In a mature almond orchard in southeastern Spain, irrigation water use was reduced under RDI while kernel yield was maintained at an economically acceptable level. Consequently, higher economic water productivity was achieved compared with FI, demonstrating that appropriately timed water deficits can increase the economic value obtained per unit of irrigation water without causing substantial yield losses [126].

The relationship between water availability and economic returns was further evaluated using water–yield response functions combined with microeconomic optimization [213]. The economic optimum under relatively unrestricted water availability was estimated at approximately 927 mm, corresponding to about 95% of FI. Under severe water scarcity, however, the maximum return per unit of water was obtained at a substantially lower irrigation level of approximately 251 mm, equivalent to 26% of FI. Under these water-limited conditions, irrigation water productivity (WP) increased from 0.27 to 0.52 kg m^−3^, while the average return per unit of water increased from €10.16 to €16.89 m^−3^ [213]. These results indicate that the economically optimal irrigation level is strongly determined by water availability and its opportunity cost rather than by yield maximization alone.

Similar economic responses have been reported in mature ‘Cartagenera’ orchards. Irrigation costs were reduced by approximately 28% under RDI, whereas yield was reduced by only about 7%. As a result, lower production costs per kilogram of almonds and higher economic returns than under FI were achieved under water-limited conditions [124].

Further evidence has been provided by long-term financial assessments. Using discounted cash-flow analysis, favourable economic returns were obtained with moderate deficit strategies, particularly SDI_75–60_, through substantial reductions in irrigation inputs combined with limited yield penalties. In contrast, profitability was reduced under more severe deficit treatments when yield losses exceeded the economic benefits derived from water savings. Thus, the economic advantage of DI is greatest when the deficit is maintained within a range in which the value of saved water compensates for the associated reduction in production [134]. In addition to irrigation-cost savings, potential economic benefits may be generated through improvements in kernel quality. Under moderate water restriction, selected kernel composition and sensory characteristics can be maintained or enhanced. However, these responses are dependent on cultivar and irrigation regime and should therefore be considered as potential rather than universal economic benefits of DI [97]. Overall, the greatest economic benefits of DI are generally obtained under moderate water restriction, whereas severe deficits may become economically disadvantageous when yield losses outweigh the value of the water saved.

### 8.11. Summary Tables

Table 3, Table 4, Table 5 and Table 6 provide a synthetic and integrated comparison of the main irrigation strategies and deficit-irrigation responses reported across the reviewed studies. The tables are intended to complement the detailed discussion presented in the preceding sections by summarizing the quantitative evidence on yield and water use (Table 3), the overall evidence for SDI, RDI, and PRD (Table 4), the strength of evidence and unresolved conclusions (Table 5), and the comparative advantages and limitations of the main irrigation approaches under water-limited conditions (Table 6). Together, these summaries highlight the strong dependence of almond responses on deficit severity, timing, duration, cultivar, tree age, and environmental conditions, while also distinguishing well-supported conclusions from areas where evidence remains limited or context-dependent.

Across the reviewed studies, the reported water-saving ranges indicate substantial potential for reducing irrigation inputs under SDI, RDI, and PRD; however, these ranges should not be interpreted as universal treatment effects because of differences in cultivar, tree age, climate, soil, deficit severity, timing, experimental duration, and methods used to quantify water use.

*Short synthesis*

Overall, almond responses to deficit irrigation depend primarily on deficit severity, timing, and duration, with substantial variation among cultivars, tree ages, and growing environments. Moderate SDI, RDI, and PRD can reduce irrigation inputs while maintaining relatively high productivity under appropriate conditions, whereas severe or prolonged deficits generally reduce yield, kernel weight, and vegetative growth. Differences among irrigation strategies are less consistent than the effects of deficit severity and timing, and PRD has not demonstrated a consistent yield advantage over RDI. Higher water productivity may occur under severe deficit despite reductions in yield, indicating that water productivity should not be interpreted independently of productivity preservation. Long-term and developmental-stage responses remain strongly context-dependent, and quantitative thresholds for optimizing the trade-off between water saving and yield preservation are still insufficiently defined.

## 9. Remote Sensing Assessment of Water Stress in Deficit-Irrigated Almonds Orchards

**Figure 12:**
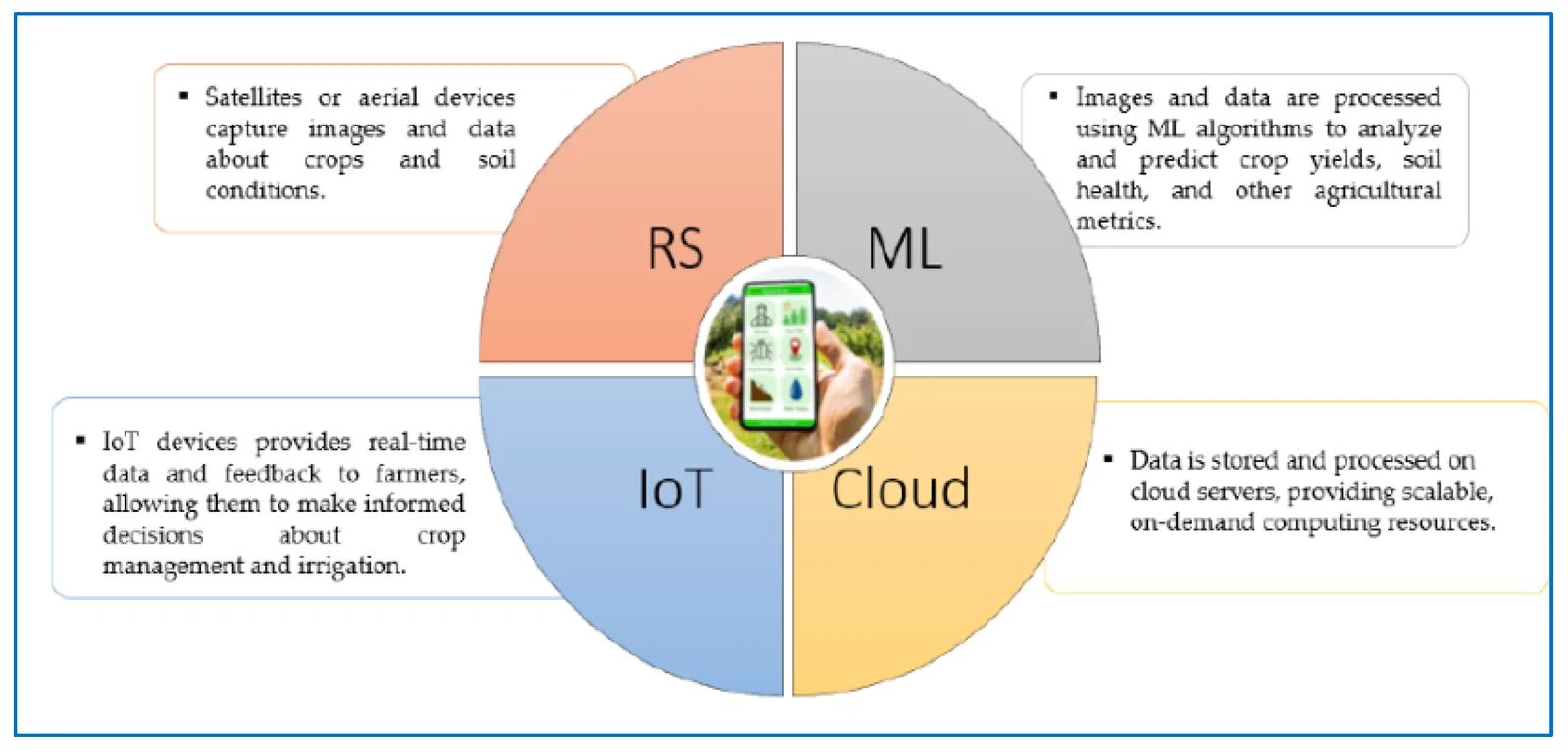
Technologies like satellite remote sensing (RS), machine learning (ML), internet of things (IoT) and internet-based computing and storage systems (Clouds) (source: Bassine et al. 2023) [214].

**Figure 13:**
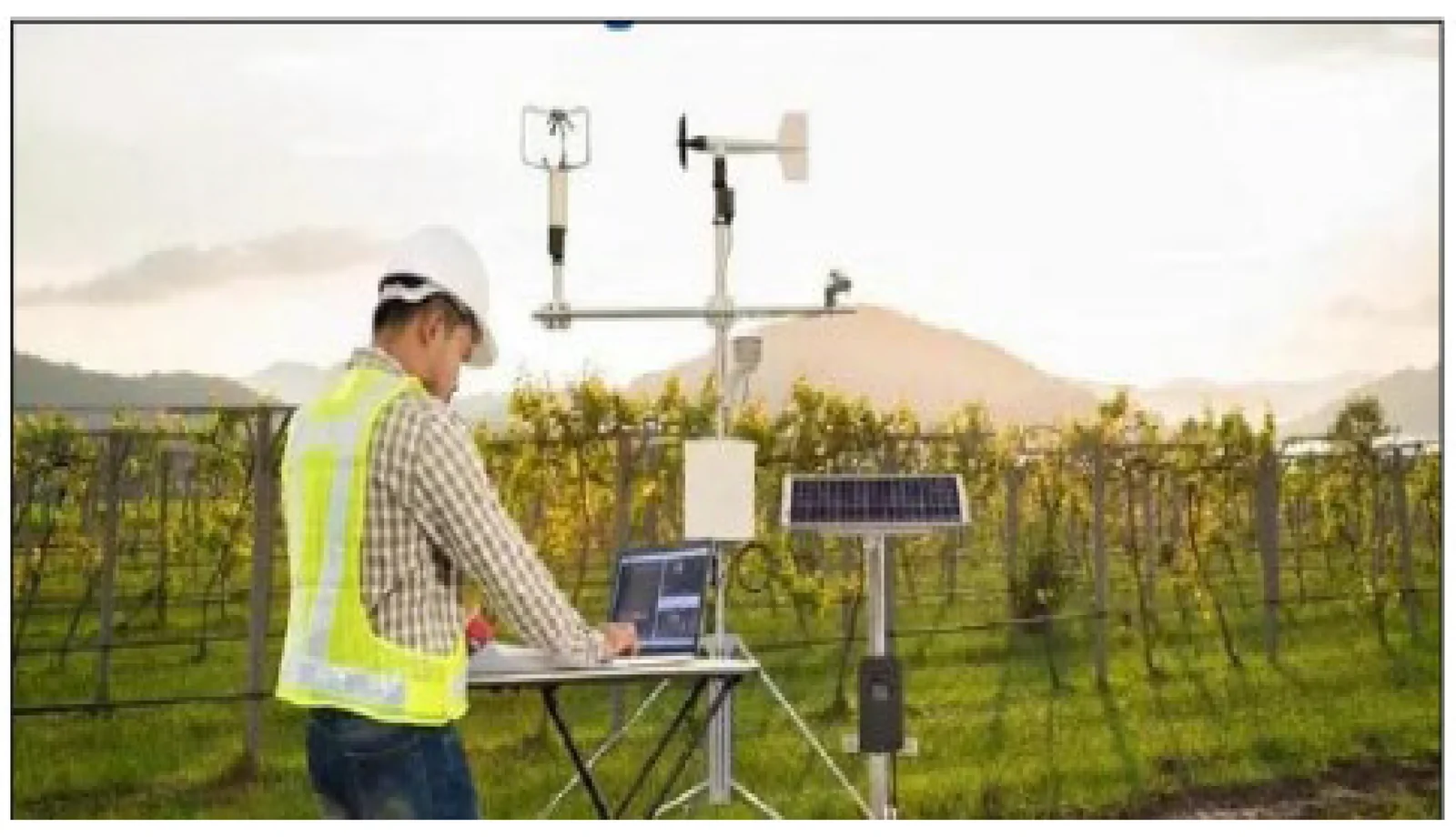
IoT-Powered Smart Irrigation System for Agriculture (source: Kishore et al., 2025) [105].

Climate-smart agriculture (CSA) supports efficient water management under scarcity by integrating AI, remote sensing, machine learning, and IoT to monitor crops, optimize irrigation, and detect stress. These technologies can improve water-use efficiency, productivity, and sustainability [105,214] (Figure 12 and Figure 13).

**Figure 14:**
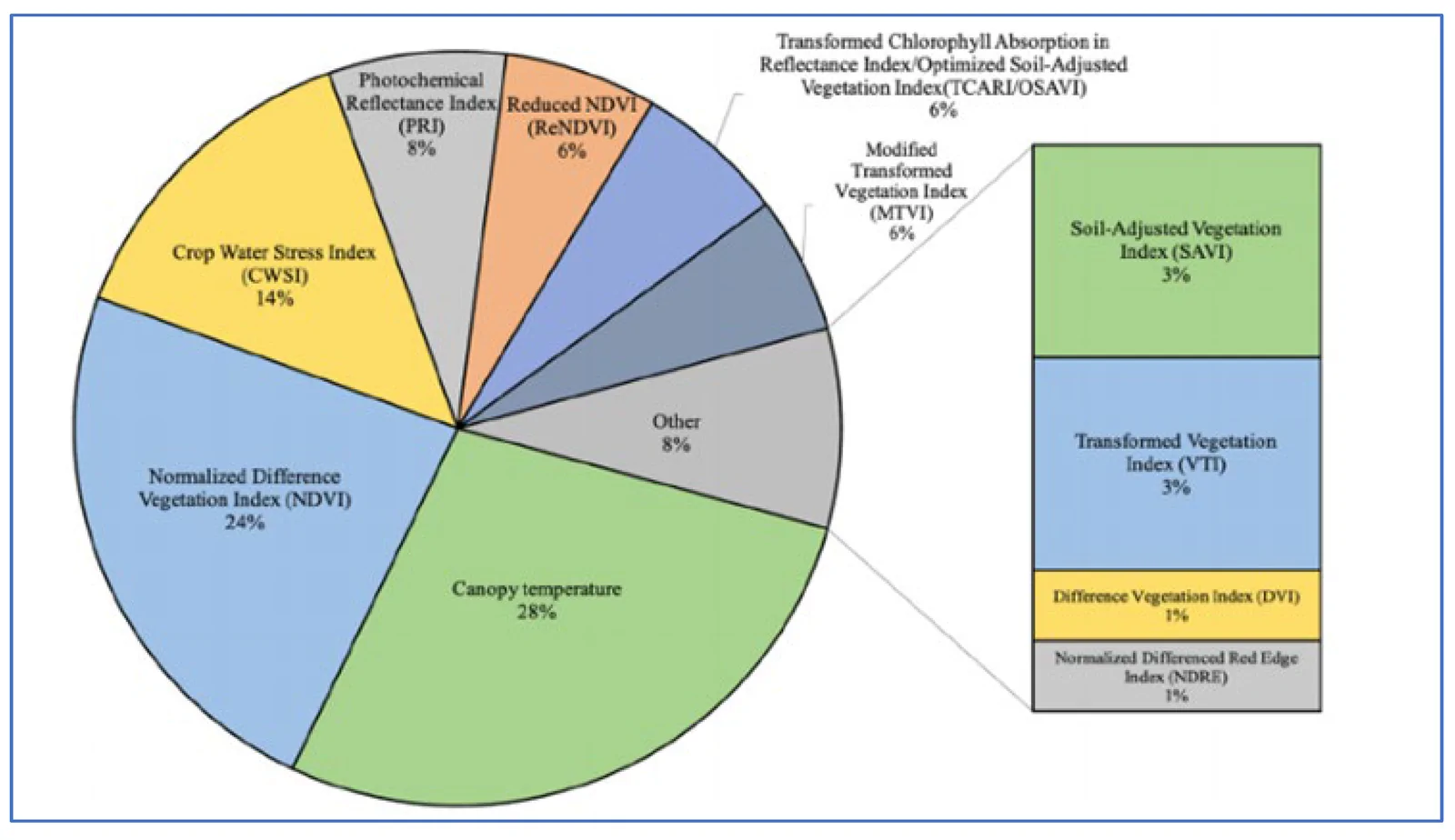
Percentage of selected studies analyzing vegetation and water stress indices using remote sensing techniques (source: Velazquez-Chavez et al. 2024) [89].

Among RS platforms, satellites are most widely used in almond orchards, followed by UAVs and MAVs [33]. RS data across UV, VIS, NIR, SWIR, and TIR regions enable vegetation and water-stress assessment using indices such as NDVI, NDRE, NDWI, EVI, SAVI, GNDVI, PRI, and CWSI. CWSI relates canopy temperature to environmental conditions to quantify plant water stress [89] (Figure 14). These indices support monitoring crop water status and precision irrigation management [33,89,215].

**Figure 15:**
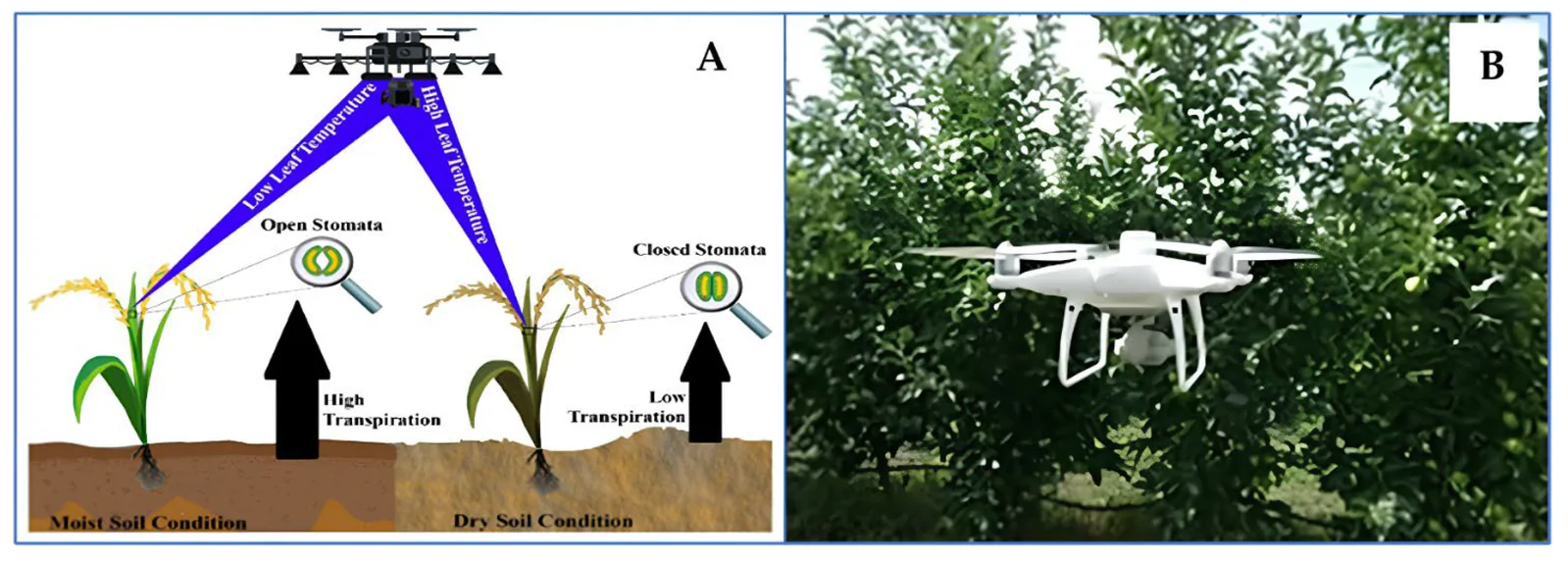
(**A**): Remote-sensing estimation of the crop water stress using leaf transpiration, temperature, cooling, and heating effects, and comparing it with the air and soil moisture levels (source: Ahmad et al., 2021) [225]; (**B**): UAS flighting inside the orchard (source: Popescu et al., 2023) [226].

**Figure 16:**
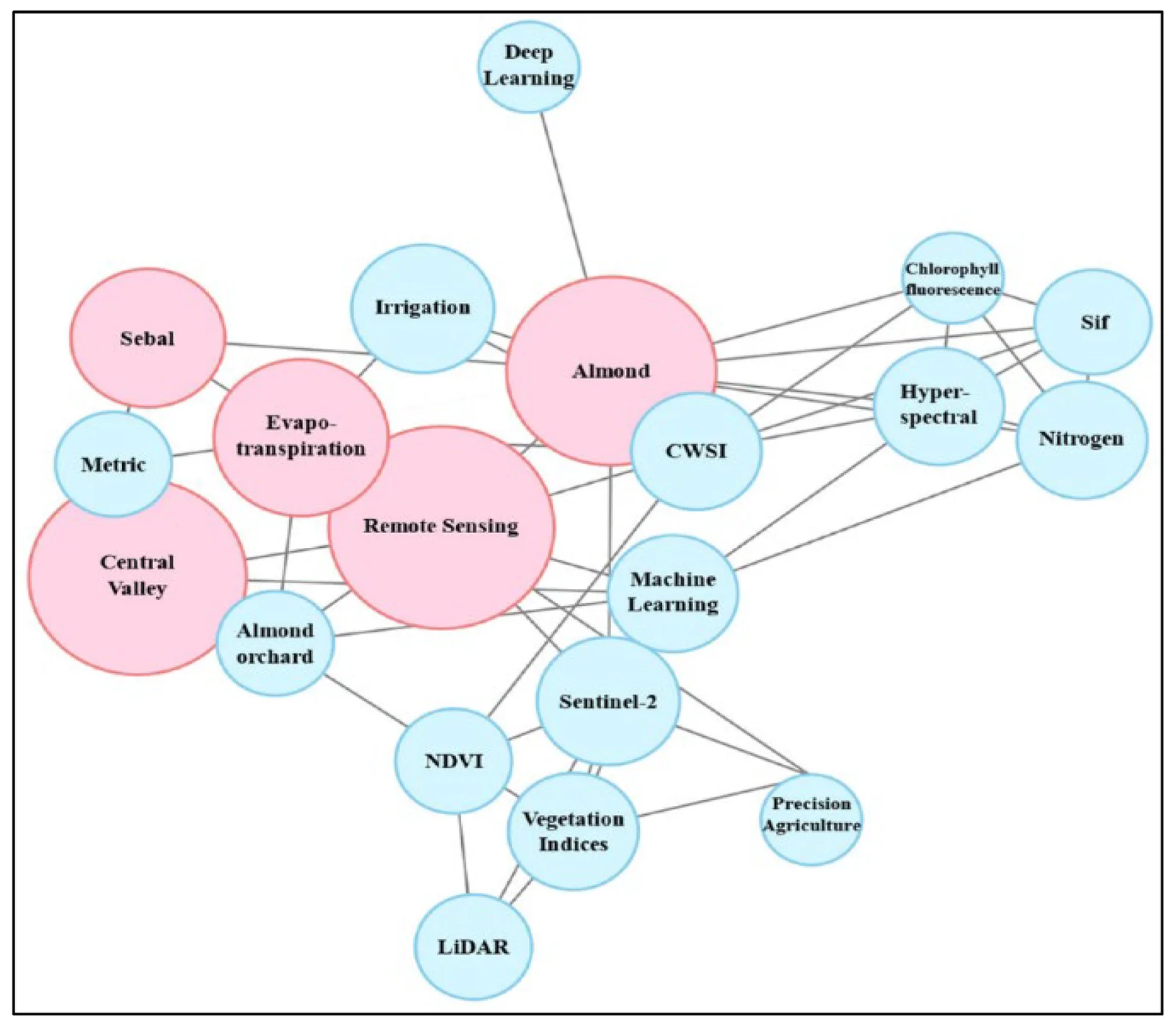
Key terms highlighted in the studies, including remote sensing, almond, Central Valley, evapotranspiration, and the Surface Energy Balance Algorithm for Land (SEBAL) (source: Guimarães et al. 2024) [33].

Sentinel-2 and Landsat are widely used for satellite monitoring, while UAVs and LiDAR systems equipped with multispectral, thermal, and RGB sensors provide higher-resolution tree-level and orchard-scale information [216]. These technologies support assessment of field variability [176,217,218], evapotranspiration estimation [27,66,219] irrigation monitoring [220,221,222,223,224,225,226], and early water-stress detection [87,176] (Figure 15 and Figure 16).

Almond water stress under DI has been assessed using UAV-based multispectral and thermal sensing combined with physiological measurements. Red-edge indices may detect mild stress more sensitively than NDVI, although NDVI remains useful for monitoring canopy vigor and irrigation responses [227,228,229]. UAV imagery can also estimate Ψstem and gs, with reported R^2^ values of up to 0.74 and 0.71, respectively [230].

Thermal sensing complements spectral approaches by detecting increases in Tc associated with reduced transpiration. Tc has been related to gs and Ψstem, while intra-crown thermal variability can indicate early stress [176]. Low-cost thermal cameras may facilitate field monitoring [87,231]. Trunk diameter fluctuation measured with LVDTs provides continuous water-status monitoring, with MDS and SIMDS used for stage-specific irrigation thresholds [135]. HYDRUS-2D has also been applied to simulate soil-water dynamics and root water uptake under contrasting irrigation regimes [118].

Physiological and remote-sensing indicators provide complementary information: Ψstem is sensitive to early stress, whereas CWSI provides greater spatial coverage; NDVI is generally less sensitive to early stress [119]. In ‘Guara’, row-level thermal imaging has supported field-scale monitoring [87]. Across ‘Guara’, ‘Lauranne’, and ‘Marta’, Ψleaf was highly responsive, while CWSI values of ≈0.12–0.15 and a Tc increase of ≈1 °C were proposed as irrigation thresholds, although cultivar-specific baselines are required [143].

Combining thermal and multispectral data improves water-status assessment, but calibration for cultivar, atmosphere, canopy structure, and baseline conditions remains necessary [231]. Satellite and aerial imagery have also supported evapotranspiration and energy-balance estimation, including TSEB, METRIC, and SEBAL approaches [28,221,232]. SCOPE-based methods have been used to assess drought-related photosynthetic responses [233]. UAV-derived CWSI has shown strong relationships with Ψstem and intrinsic WUE, while pigment- and NIR-based indices provide complementary information on physiological status and yield [119,234]. Multispectral indices such as NDVI, GNDVI, and NDRE combined with CWSI have further improved Ψstem prediction [218].

Fluorescence-based sensing, including SIF and FRF, provides additional information on water-stress responses but remains limited by canopy heterogeneity, sensor requirements, and validation [217,235]. Satellite observations combined with TSEB enable spatial ETa estimation, while UAV-TSEB estimates of transpiration have agreed with sap-flow measurements [36,66,232]. Machine learning and IoT approaches integrating vegetation indices, canopy temperature, soil moisture, weather, and automated control further support precision irrigation [120,156,236,237]. LiDAR and thermal sensing combined with water-balance models have also been applied to irrigation scheduling [156]. An integrated model using fAPAR, NDMI, canopy chlorophyll, ETa, and meteorological variables achieved an RMSE of 399.1 kg ha^−1^ for yield prediction, which has the same units as the response variable, in this case crop yield [238]. okNIRS provides complementary non-destructive assessment of almond quality and should be considered separately from orchard water-stress monitoring [239].

## 10. General Discussion

The findings synthesized in this review complement previous studies by demonstrating that almond responses to deficit irrigation (DI) cannot be fully interpreted from irrigation amount or yield response alone. Previous research has provided important evidence on irrigation regimes and productivity [26] nut quality [27], physiological responses to water deficit [28,31], sustainable deficit-irrigation strategies [29,30], and remote-sensing (RS) approaches for monitoring crop water status [32,33]. By considering these dimensions together, the present synthesis highlights the relationships among irrigation amount and timing, plant water status (PWS), physiological responses, water-use efficiency (WUE), productivity, and nut quality. The reviewed evidence indicates that the effects of DI are strongly dependent on environmental conditions, phenological stage, cultivar, soil characteristics, and the severity and timing of water restriction. Consequently, water-saving responses cannot be generalized across almond production systems without considering these interacting factors.

An important implication emerging from this synthesis is the potential value of combining physiological measurements with RS approaches for characterizing PWS and supporting irrigation scheduling. However, the available evidence remains heterogeneous, and the translation of physiological and RS indicators into consistent irrigation recommendations requires further validation across cultivars, environments, and management conditions. The present review therefore extends previous syntheses by integrating irrigation management, productivity, nut quality, physiological responses, WUE, and PWS monitoring within a common framework. Rather than focusing on a single DI strategy or response variable, the synthesis considers the interactions and trade-offs among these components and identifies areas where the available evidence remains inconsistent or insufficient. Future research should prioritize integrated experiments that simultaneously evaluate irrigation regime, plant physiological responses, water use and productivity, nut quality, and water-status indicators. Such approaches would help clarify the trade-offs between water savings and production outcomes and strengthen the practical application of DI for sustainable almond production.

The effectiveness of regulated deficit irrigation (RDI) and sustained deficit irrigation (SDI) depends strongly on irrigation timing because almond-tree sensitivity to water deficits varies among phenological stages. Full irrigation (FI) is generally recommended during the most water-sensitive stages, including flowering, fruit set, and periods of rapid fruit growth, because water deficits during these stages can strongly affect yield formation. Water deficits during the postharvest period can also have substantial negative effects on subsequent tree growth and productivity, even when reductions in applied irrigation water are smaller than those imposed during the preharvest period. Nevertheless, moderate water deficits have been successfully imposed during periods of rapid fruit growth and/or postharvest without substantial reductions in yield or tree performance in some field studies [113,135,148].

Controlled water deficits can also be imposed during relatively less water-sensitive periods, particularly during kernel filling, which coincides with periods of high evaporative demand, to improve WUE while minimizing reductions in yield [50,122]. This response may be associated with the strong sink activity of developing kernels, which can maintain assimilate partitioning toward reproductive organs during moderate water deficits [69,113]. However, severe water stress during kernel filling can impair physiological functioning, reduce yield, and potentially cause carry-over effects on tree performance in subsequent seasons [55,57,151]. Moreover, because final nut size is largely determined during the fruit-development period, excessive water stress can reduce kernel and nut size and consequently decrease market value [71].

Despite the potential suitability of kernel filling for DI, almond responses to RDI during this stage have been inconsistent. Significant reductions in kernel yield have been reported under some RDI strategies, whereas no significant yield reductions have been observed under other irrigation treatments with comparable levels of water restriction [68,132,136,141]. These contrasting responses may be attributed to differences in the magnitude, timing, and duration of water deficits, as well as variations in cultivar, climatic conditions, soil properties, orchard age, and irrigation system. Differences in the amount and distribution of applied irrigation water among DI strategies may also contribute to the variability observed among studies.

Cultivar-specific responses further highlight the importance of considering genetic differences when evaluating DI strategies. For example, cv. ‘Marta’ maintained yields comparable to those obtained under FI, which was associated with a conservative response to water stress, whereas cv. ‘Lauranne’, despite exhibiting physiological responses similar to those of cv. ‘Guara’, achieved the highest yield under moderate-to-severe SDI conditions [116]. Soil properties may also influence the effectiveness of DI strategies, although their role in almond production remains insufficiently documented. In particular, the effectiveness of partial root-zone drying (PRD) can be influenced by soil texture and hydraulic properties, because water redistribution is generally more pronounced in the vertical direction in coarse-textured soils, whereas finer-textured soils can promote greater lateral water movement. Early irrigation cut-off can induce water stress, resulting in stomatal closure, premature leaf senescence, and reductions in vegetative growth and yield; however, the magnitude of these effects may be moderated by greater soil water-holding capacity and differences in irrigation-system characteristics [56].

Orchard age is another important factor affecting almond water requirements and responses to DI. Young trees have relatively low crop water requirements because of their smaller canopy size, but their water requirements and crop coefficients (Kc) increase rapidly as canopy development progresses. In young almond orchards, Kc and water use have been shown to increase substantially during the first four years after planting, highlighting the importance of considering orchard age when establishing irrigation requirements [47]. Cultivar-specific differences in the response of young almond trees to SDI have also been reported, with differences in yield response observed among cultivars such as ‘Marta’, ‘Lauranne’, and ‘Guara’ [116]. More recently, the effects of irrigation strategy and production system on almond yield and quality have been shown to vary with orchard age, with differences becoming more pronounced as trees approach full production [155].

Across several studies, no consistent differences between RDI and SDI have been reported, with responses depending on the timing, severity, and total amount of water applied [54,58,69,100,137]. However, higher yields under RDI than SDI have been reported under some conditions when comparable irrigation volumes were applied [71]. Similarly, PRD has generally not provided clear additional benefits over RDI or SDI when equivalent amounts of irrigation water were supplied [130]. In contrast, low-frequency deficit irrigation (LFDI) imposed during kernel filling has been shown in some studies to maintain yield while reducing irrigation inputs, indicating its potential as a water-saving strategy.

Long-term studies further indicate that mild and short-term water deficits can be compatible with sustained productivity, whereas prolonged or severe water stress can reduce vegetative growth and result in cumulative yield losses. Consequently, long-term irrigation strategies should account for climatic conditions, irrigation management, orchard age, cultivar, and the magnitude and timing of water deficits to maximize water savings while maintaining yield close to that obtained under FI [240].

Regarding kernel quality, moderate DI generally has limited effects on the major nutritional and physicochemical characteristics of almonds, although changes in specific fatty acids, sugars, organic acids, and other quality attributes may occur depending on cultivar and irrigation regime [53]. Experimental studies have also shown that moderate RDI can maintain overall almond quality while producing changes in selected compositional and sensory characteristics [53]. However, severe water stress can adversely affect kernel development and some quality attributes [50]. Thus, the response of almond quality to DI depends on the intensity and timing of water deficits, as well as on cultivar and environmental conditions. Overall, moderate DI can improve water productivity (WP) and WUE while maintaining acceptable yield and kernel quality under appropriate management conditions [72].

Under severe water limitations, complementary management practices may help reduce crop water requirements. For example, canopy management through mechanized disc pruning has been investigated as a strategy for reducing canopy size and water demand; however, its effects on stem water potential, stomatal conductance, leaf transpiration, and yield should be considered when evaluating its suitability as a complementary strategy to DI [156]. More broadly, DI can improve WUE and WP under limited water availability by increasing production or economic returns per unit of water consumed, provided that the imposed water deficit remains within the tolerance limits of the crop [25,72].

The effectiveness of DI ultimately depends on accurate monitoring of crop water status and timely irrigation management. In this context, RS has become an increasingly important tool for the non-destructive and spatially distributed assessment of almond water status. RS applications have been used to estimate evapotranspiration (ET), characterize canopy temperature (Tc) and crop water stress, and support irrigation management. Among the available platforms, satellite observations provide broad spatial coverage, whereas UAV and airborne platforms offer higher spatial resolution for orchard-scale and tree-level assessment. The integration of multispectral and thermal imagery with energy-balance models has improved the characterization of evapotranspiration and plant water status in almond orchards [34,156,241].

Satellite-derived vegetation indices and remotely sensed crop coefficients (Kc) have also been used to monitor irrigation and quantify water use in almond orchards. Landsat and Sentinel observations have been integrated with soil-water-deficit models, field measurements, and energy-balance approaches to estimate irrigation requirements, actual evapotranspiration, and crop responses to different irrigation and soil-management practices [36,223,225]. Overall, combining multispectral and thermal indicators, particularly red-edge indices and the crop water stress index (CWSI), with physiological measurements provides complementary information for assessing almond water status and has potential to support irrigation management under DI. However, further validation across cultivars, environments, orchard ages, and irrigation systems is needed before these approaches can be consistently translated into operational irrigation recommendations.

### Limitations of the Review

Several limitations should be considered when interpreting the findings of this review, First, the studies included in the review varied considerably in cultivar, tree age, soil characteristics, climatic conditions, irrigation systems, severity and timing of water restriction, and experimental duration. These differences make direct comparisons among studies difficult and may contribute to inconsistencies in reported responses to deficit irrigation. Second, many experiments were conducted over relatively short periods, whereas the effects of repeated deficit irrigation over multiple seasons remain less well documented. This is particularly important for assessing cumulative effects on tree health, productivity, and orchard longevity. In addition, economic assessments are strongly influenced by local water costs, energy prices, almond market values, and production conditions, limiting the transferability of economic results among regions. Finally, the increasing use of remote sensing for water-stress assessment in almond orchards is promising, but differences in sensors, spatial and temporal resolution, environmental conditions, and validation approaches currently limit the comparability and broader application of reported results. These limitations highlight the need for standardized, multi-year field studies and integrated approaches combining irrigation, physiological measurements, economic assessment, and remote sensing.

## 11. Conclusions

Increasing water scarcity under climate change is challenging almond production in arid and semi-arid regions, highlighting deficit irrigation (DI) as an important strategy for improving water-use efficiency (WUE). This review shows that the effectiveness of SDI, RDI, PRD, and LFDI depends strongly on cultivar, orchard age, soil and climate, deficit intensity and duration, and irrigation timing. Among these strategies, appropriately timed and moderate RDI generally provides a favorable balance between water savings and productivity, whereas severe or prolonged deficits can reduce yield, kernel development, and tree performance. Kernel quality and economic returns should therefore be considered alongside water savings when designing DI strategies.

Reliable irrigation management also requires accurate assessment of plant water status. Ψstem and Ψleaf provide sensitive physiological indicators, while thermal and multispectral sensing offer complementary information with greater potential for spatial monitoring. However, water-status thresholds require cultivar- and site-specific calibration. Future research should prioritize standardized methodologies and long-term commercial trials to improve comparability and assess the cumulative effects of DI on productivity, orchard longevity, and soil health. The integration of proximal, UAV, and satellite sensing with physiological measurements, evapotranspiration models, crop-water balances, and machine learning offers promising opportunities for adaptive, precision irrigation. Overall, sustainable almond production under increasing water scarcity will depend on combining well-timed DI with site-specific monitoring and decision-making to improve WUE while maintaining yield, kernel quality, and long-term orchard resilience.

## Figures and Tables

**Table 1:** Comparison of previous reviews related to almond irrigation, deficit irrigation, plant physiology, nut quality, and remote sensing, and the scope of the present synthesis.

Reference	Main Scope	Main Focus	Geographical/Crop Scope	Main Aspects Covered	Main Aspects Insufficiently Integrated	Contribution of the Present Review
[26]	Irrigation regimes and yield responses	Effects of irrigation supply on almond productivity	Mediterranean almond production systems	Irrigation treatments; yield responses; productivity	Plant physiological indicators; nut quality; precision irrigation; remote sensing	Integrates irrigation strategies with physiology, nut quality, water productivity, and precision-irrigation approaches
[27]	Almond nut quality and sensory assessment	Nutritional, physicochemical, and sensory characteristics	Almond	Nutritional composition; physicochemical properties; sensory quality	Interaction between irrigation management and nut quality	Links irrigation regimes and water availability with physiological responses and nut-quality attributes
[28]	Physiological and agronomic responses to RDI	Mechanisms underlying plant adaptation to water deficit and improved WUE	Broad crop perspective	RDI; plant physiology; water deficit; WUE; adaptation mechanisms	Almond-specific irrigation challenges; nut quality; remote sensing; precision irrigation	Provides an almond-specific synthesis integrating RDI/DI, physiological responses, productivity, and water-management strategies
[29]	Sustainable irrigation intensification and DI	Improving WUE while maintaining yield under water-limited conditions	Almond and pistachio	Sustainable irrigation; DI; WUE; yield responses; water scarcity	Almond-specific integration of nut quality, physiological indicators, and remote-sensing-based irrigation management	Extends the focus specifically to almond and integrates irrigation, physiology, nut quality, water productivity, and monitoring technologies
[30]	DI strategies for almond production	Effects of DI timing and severity on almond production	California almond production	DI management; timing; deficit severity; production responses	Broader physiological basis; nut quality; water productivity; remote sensing; applicability beyond California	Provides a broader and more integrated assessment across production environments, physiological indicators, quality, and precision irrigation
[31]	Physiological basis of almond irrigation management	Physiological indicators for irrigation scheduling	Almond	Water potential; stomatal conductance; leaf temperature; phenological stage; cultivar-specific responses	Integration with DI strategies, yield, nut quality, and remote-sensing technologies	Connects physiological indicators with irrigation strategies, productivity, quality, and remote-sensing applications
[32]	Remote sensing for irrigation monitoring	Monitoring crop water status and irrigation requirements	Broad horticultural crop perspective	Crop water status; evapotranspiration; canopy temperature; precision irrigation	Almond-specific applications; integration with irrigation strategy, physiology, productivity, and nut quality	Evaluates remote sensing specifically within an integrated almond irrigation-management framework
[33]	Remote sensing in almond orchards	Applications of satellite, UAV, and proximal sensing	Almond orchards	Satellite imagery; UAVs; proximal sensing; water management	Integration of sensing technologies with irrigation strategies, physiological responses, productivity, and WUE	Integrates remote sensing and plant water-status monitoring with irrigation management, physiological responses, yield, and water productivity

Against this background, the present review differs from the previous literature by providing an integrated synthesis of deficit irrigation in almond production, linking irrigation strategies with water use and productivity, yield and nut quality, plant physiological responses, and plant water status monitoring through physiological and remote-sensing approaches. Rather than examining these components independently, we critically consider their interactions and trade-offs under different irrigation regimes, environmental conditions, phenological stages, and management strategies. This integrated perspective enables the identification of knowledge gaps concerning the relationships between irrigation intensity and timing, water savings, productivity, nut quality, and physiological responses, as well as the potential of plant water status and remote-sensing indicators to support irrigation scheduling. Thus, the present synthesis extends previous reviews by connecting established knowledge on deficit irrigation, almond productivity, and plant physiology with emerging approaches for monitoring crop water status, providing a comprehensive framework for improving water-efficient almond production under increasing water scarcity.

**Table 2:** Summary of published single-season and multi-season studies on deficit irrigation strategies and remote sensing applications in almond orchards (the abbreviations are defined at the beginning of the article).

Study/Country	Irrigation Treatments and Time of Application	Cultivar/ *Tree Age*	Study Duration (Years)	Planting Density	Main Results
Fereres et al., 1981 [56] California	*Three treatments:* (i) FI. (ii) *Cut-off* in early stage IV (Sept.). (iii) *Cut-off* in late stage IV (Oct–Nov.).	’Nonpareil’ grafted Nemaguard *2 years old*	4	6 × 6 [56]	Irrigation termination in September in young trees caused drought stress leading to leaf senescence and reduced growth and yield.
Hutmacher et al., 1994 [110] California	*Six treatments:* (i) SDI_50_. (ii) SDI_75_. (iii) SDI_100_. (iv) SDI_125_. (v) SDI_150_. (vi) SDI_175_.	’Ruby’ grafted Nemaguard *Adult*	3	6 × 6	Tree growth, nut yield, and plant water status indicators improved significantly with increases in applied water up to the SDI_125_ level.
Girona et al., 1993 [121] Spain	*Four treatments:* (i) FI. (ii) SDI_130_. (iii) SDI_70_. (iv) RDI_70-III_.	’Ferragnes’ Grafted GF-677 *6 to 10*	3	5 × 6	Uniform seasonal deficits SDI_70_ reduced yield RDI_70-III_ maintained near-normal yields.
Goldhamer and Smith, 1995 [55] California	*Five treatments:* (i) FI. (ii) SDI_100_ up to 406 mm. (iii) SDI_75_. (iv) SDI_50_. (v) Variable: 80% through mid-May; 60% through mid-June; 40% through early-July; 60% until harvest.	’Nonpareil ’ grafted Nemaguard *Mature*	3	6 × 6	Adequate irrigation after a drought year, particularly during bud development (August–September), is essential to maintain soil moisture and ensure successful bloom and fruit set in the following season.
Girona et al., 1997 [122] Spain	*Five treatments:* (i) FI. (ii) RDI_I-_*cut-off*. (iii) RDI_II_-*cut-off*. (iv) RDI_III_-*cut-off*. (v) SDI *continuous cut-off.*	’Marcona ’ grafted GF-677 *16-Adult*	3	5 × 5	Irrigation cut-off during early stages I and II significantly reduces yield (25–45%), whereas Stage III (preharvest) is relatively tolerant, with minimal yield loss (0–15%). Continuous water deficit throughout the season (SDI) was not a suitable irrigation strategy.
Goldhamer and Viveros, 2000 [123] California	*Three treatments:* (i) FI. (ii) RDI_III_-*cut-off*. (iii) RDI_IV-_*cut-off*.	’Nonpareil ’ grafted to Nemaguard *Adult*	2	6 × 6	Preharvest cut-off: mainly affected kernel size and harvest quality, with relatively limited impact on subsequent yield. Postharvest water deprivation: caused major reductions in the next year’s crop, with ~74% maximum kernel yield loss under severe stress.
Klein et al., 2001 [111] California	*Two treatments:* (i) FI. (ii) RDI_IV_-*cut-off*.	’Nonpareil ’ grafted Nemaguard *Adult*	3	6 × 8	Postharvest-period irrigation cut-off accelerates leaf senescence and reduces function and persistence in mature almond trees.
Esparza et al., 2001a [112] California	*Two treatments:* (i) FI. (ii) RDI_IV_-*cut-off*	’Nonpareil ’ grafted Nemaguard *7 to 9*	3	6 × 8	Water stress during postharvest has little immediate effect but reduces yield in the following season.
García et al., 2004 [124] Spain	*Two treatments:* (i) FI. (ii) ssRDI_20-III_.	’Cartagenera’ grafted Franco *13 to 17*	4	7 × 5	A 28% saving in irrigation water was achieved with RDI during the kernel-filling stage, while almond production decreased by only 7%. Despite this slight reduction in yield, RDI was considered economically and agronomically appropriate under semi-arid conditions, owing to a 10% reduction in annual operating costs and improved water-use efficiency.
Romero et al., 2004b [125] Spain	*Two treatments:* (i) FI. (ii) ssRDI_20-III_.	’Cartagenera’ grafted Franco *13*	2	7 × 5	Very severe RDI reduced g_s_, A_n_ and Tr by 64%, 67% and 56%, respectively, and increased leaf abscission while reducing leaf growth. Kernel yield was reduced by 7% (not significant), whereas IWU decreased by 30% and WUE improved. RDI was considered suitable when Ψpd remained above −2 MPa.
Romero et al., 2004a [113] Spain	*Five treatments*: (i) FI *_surface drip_*. (ii) RDI_20-III_-*_surface drip_*. (iii) RDI_20-III-*subsurface drip*_. (iv) RDI_20-III and 75-IV *subsurface drip*_. (v) RDI_20-III and 50-IV *subsurface drip*_.	’Cartagenera’ grafted Franco *15*	4	7 × 5	Moderate water stress is safe; very severe RDI_20_-during kernel filling and moderate to severe RDI_50_-postharvest stages reduces vegetative growth and leaf area while yield was maintained with no significant reduction. WUE was improved under subsurface drip irrigation.
Romero et al., 2006 [126] Spain	*Five treatments* (similar to the previous article).	‘Cartagenera’ grafted Franco *15*	4	7 × 5	RDI reduced IWU by approximately 30% and irrigation costs by 29%, while yield was reduced by 7% but remained statistically nonsignificant. Net returns were maintained, and water-use efficiency was improved under subsurface drip irrigation.
Girona et al. 2005 [58] Spain	*Three treatments:* (i) SDI_130_ (*over-irrigation*) (ii) mSDI_70_; (iii) ssRDI_20-III._	‘Ferragnes’ grafted GF-677 *4 to 6*	4	5 × 6	Very severe RDI_20_ during kernel filling stage maintained yield in the first two years, but long-term reductions of ~20% were observed; therefore, RDI must be carefully managed.
Goldhamer et al., 2006 [71] California	*Seven treatments*: (i) FI. (ii) SDI _55_. (iii) SDI_75_. (iv) SDI_85_. (v) RDI_55-I_. (vi) RDI_75-II_. (vii) RDI _85-III_.	‘Nonpareil’ grafted Nemaguard *15 to 18*	4	6 × 6	Seasonal irrigation level significantly affected almond yield. The highest yields were obtained at 85% ETc, whereas 70% ETc caused moderate yield reductions and 55% ETc resulted in substantial yield losses. Postharvest water stress maintained higher yields than preharvest stress, while SDI produced intermediate responses.
Nortes et al., 2005 [107] Spain	*Three treatments:* (i)SDI_120 (control)_. (ii) SDI_60_. (iii) RDI_40-III_.	‘Marta’ grafted Mayor *2-young*	1	7 × 6	Trunk growth rate (TGR) was more sensitive to water stress than maximum daily trunk shrinkage (MDS) and *Ψ*_pd_ and *Ψ*_st_ indicating its usefulness for quantifying water-stress intensity and guiding irrigation decisions in young almond trees.
Nortes et al., 2008 [127] Spain	*Two experimental plots:* ***(a)*** Irrigation with moderate saline water (4.2 dS m^−1^). *Three treatments:* (i) SDI_125 (control)_. (ii) RDI_70-II_, _30-III, 70-IV_. (iii) Rainfed.	Colorada’ (*autochthona*) *Adult*	4	6 × 6	The autochthonous variety showed minimal yield reduction, tolerating deficit irrigation even with saline water. In contrast, young ‘Marta’ trees experienced a strong yield decline under continuous SDI_60_, while RDI_40_ and RDI_20_ during kernel filling stage helped limit stress.
	***(b)*** Irrigation with fresh water: *Five treatments:* (i) SDI_120_ _(control)_. (ii) SDI_80_. (iii) SDI_60_. (iv) RDI_40-III_. (v) RDI_20-III_.	‘Marta’ grafted Mayor *2 to 4* *young*	3	7 × 6	
Nortes et al., 2009 [128] Spain	*Two treatments* (i) FI. (ii) mo-sSDI_50_.	‘Marta’ grafted Mayor *5*	1	7 × 6	Moderate to severe SDI_50_ did not alter seasonal leaf structure or photosynthetic capacity but reduced leaf mass and nitrogen content more in non-fruiting leaves than in fruiting leaves.
Egea et al. 2010 [109] Spain	*Two treatments:* (i) FI. (ii) mRDI_70-III_.	‘Marta’ grafted Mayor *7 to 10*	3	7 × 6	Mild RDI_70_ during kernel filling reduced IWU by about 30% without affecting almond yield, despite temporary reductions of over 50–60% in photosynthesis, stomatal conductance, and transpiration.
Egea et al. 2012 [129] Spain	*Three treatments:* (i) FI. (ii) mRDI_70-III_. (iii) sSDI_50_.	‘Guara’ grafted GF-677 ***5***	1	6 × 7	DI progressively reduced physiological performance and yield. Mild RDI_70_ maintained it, severe SDI_50_ caused major losses.
Egea et al., 2009 [130] Spain	*Five treatments* (i) FI. (ii) RDI_50-III_. (iii) PRD_30-SDI_. (iv) PRD_50-SDI_. (v) PRD_70-SDI_.	‘Marta’ grafted Mayor *5 to 7* *Adult*	3	7 × 6	Continuous PRD_30–50-SDI_ cut kernel weight by 15–20%, whereas msRDI_50_ during kernel filling reduced it only 3–7%. Similar performance is observed under PRD_70_ and RDI when annual water supply is comparable. Overall, PRD did not present a clear advantage (or disadvantage) over RDI with regard to almond fruit growth and quality.
Cornacchia et al., 2010 [131] Spain and Italy	*Four treatments:* (i) FI. (ii) PRD_50_. (iii) RDI_30-III_. (iv) Rainfed.	‘Marta’ grafted Mayor *Adult*	1	7 × 6	PRD and RDI reduce WU while maintaining yield and quality comparable to FI. These water-saving strategies are considered sustainable without clear evidence of reduced storage viability. Rainfed showed lower yield and poorer nut quality.
Stewart et al., 2011 [132] California	*Two treatments:* (i) FI. (ii) mRDI_85-iii-IV_.	‘Nonpareil’ grafted Nemaguard *9 to14* *Adult*	5	3.7 × 7.3	Maintaining Ψstem values −1.4 to −1.8 Mpa for control and RDI treatments during hull split (final kernel filling stage) saved about 127 mm of water annually. After five years, yield was unaffected, although kernel weight was slightly reduced. These results show that water savings are possible without yield loss, even in low water-holding capacity soils.
García-Tejero et al., 2011 [133] Spain	*Three treatments:* (i) FI; (ii) RDI_30-III_ (iii) LFDI: based on Ψstem thresholds around −2.0 Mpa	‘Guara’ grafted GF677 *5*	1	7 × 6	LFDI under mild-to-moderate stress increased productivity 16% over FI and out performed RDI_30_ during the kernel filling.
Egea et al., 2013 [69] Spain	*Four treatments:* (i) FI. (ii) sRDI_40-III_. (iii) moSDI_75–60_ (iv) msSDI_60–30_	‘Marta’ grafted Mayor *2 to 6* *young to* *adult*	6	7 × 6	Long-term deficit irrigation reduced vegetative growth and yield, with the magnitude of reduction depending on irrigation severity, while IWP increased proportionally to the mean relative water shortage.
Alcon et al., 2013 [134] Spain	*Four treatments:* FI (ii) sRDI_40 III_; (iii) mmSDI_75–60_. (iv) msSDI_60–30_. (*similar to previous article*).	‘Marta’ grafted Mayor *2 to 6*	6	7 × 6	A discounted cash flow analysis (DCFA) showed that mild to moderate SDI_75–60_ achieved greater financial savings than RDI_40-_ during kernel filling period.
Puerto et al., 2013 [135] Spain	*Four irrigation treatments:* (i) FI. (ii) RDI_1_ based on SI**_MDS_**. (iii) RDI_2_, based on SI**_MDS_**. (iv) FRM (farmer treatment). Sensor-based irrigation management.	‘Guara’ grafted GF-677 *12*	2	7 × 6	RDI strategies based on maximum daily trunk shrinkage signal intensity (SI**_MDS_**) were able to generate moderate to severe water stress in almond trees, which resulted in seasonal water savings around 50%. Moderate RDI caused little yield loss and was more cost-effective than FI or farmer deficit irrigation.
Phogat et al., 2013b [118] Australia	*Two irrigation treatments:* (i) FI_p_. (ii) FI_c_ SDI_65p_. *The model evaluated the daily fluctuations in water under pulsed (FI_p_) and SDIand continuous (FI_c_) irrigation.* HYDRUS-2D modelling.	‘Nonpareil’ Rootstok not reported *Adult*	1	6 × 7	Pulsed subsurface drip irrigation (SDIp) improved soil-water distribution and maintained tree water status under sustained stress. Compared with conventional continuous drip irrigation, SDIp increased water productivity by 37%, while yield was reduced by 8% and irrigation water use was reduced by 35% relative to full irrigation. HYDRUS-2D accurately predicted soil water and salinity dynamics.
Razouk et al., 2013 [136] Morocco	*Three treatments:* (i) FI. (ii) m-sRDI_50 II-III_. (iii) ssRDI_25 II-III_. *Duration: ~30–45 days (mid-season)*.	‘Tuono’ Rootstok (not reported) *Adult*	4	6 × 5	Moderate to severe RDI_50_ reduced water use by approximately 50% while maintaining stable yield, whereas very severe RDI_25_ decreased yield by 10–15%. RDI_50_ enhanced almond kernel oil concentration, and soluble solids content (SSC) increased under both RDI levels.
Mañas et al., 2014 [137] Spain	*Six treatments:* (i) FI. (ii) SDI_25_. (iii) RDI_50-I-II-IV_ and _15-III_. (iv) mo-s SDI _50_. (v) ssRDI_20-III_. (vi) Near-rainfed *(irrigation only to keep trees alive)*.	Cultivar and Rootstock not reported *Adult*	4	7 × 5	FI produced the highest yield (≈1260 kg ha^−1^). RDI and SDI showed no significant differences in yield or WUEI. Moderate to severe SDI_50_ and very severe RDI_20-III_ reduced yield by 23% and 31%, respectively, but saved 50% and 55% of irrigation water.
Zhu et al., 2015 [138] Australia	*Five treatments:* (i) FI. (ii) SDI_120_. (iii) mmSDI_85_. (iv) mSDI_70_. (iv) m-sSDI_55_.	‘Nonpareil’ grafted Nemaguard 7-*Adult*	3	5 × 5	Moderate water deficits can substantially reduce irrigation water use while maintaining the fatty acid composition and tocopherol content he oleic/linoleic ratio increased under moderate water deficiency, but decreased under severe and extreme water deficiency.
Garcia-Tejero et al. 2018 [139] Spain	*Three treatments* (i) FI, (ii) RDI_50-III_, (iii) LFDI, *using RS and CWSI*.	‘Guara’ grafted GF-677 *5-Young*	2	6 × 5	Thermal RS accurately detected irrigation-induced water stress in almond trees. Deficit irrigation increased canopy temperature and CWSI values, which were closely related to plant water status. The study confirmed thermal imaging as a useful tool for rapid and non-destructive irrigation management.
Espadafor et al., 2017 [140] Spain	*Three treatments*: (i) FI. (ii) mSDI_55_. (iii) SDI_38_. (iv) Rainfed.	‘Guara’ grafed GF-677 *Adult*	1	6 × 7	Moderate water deficit reduced almond tree transpiration below a Ψstem threshold of −1.1 MPa, reaching approximately 50% of the fully irrigated control at −1.6 MPa. Transpiration efficiency was not improved by water deficit.
Monks et al., 2017 [141] Australia	*Height treatments:* (i) FI. (ii) RDI_120 (over irrigated)_. (iii) RDI_85-III_. (iv) RDI_70-III_. (v) RDI_55-III_. (vi) SDI_85_. (vii) SDI_70_. (viii) SDI_55_.	Cultivar, Rootstock and tree ages not reported	3	7 × 5	Irrigation RDI and SDI at 85% of FI maintained kernel yield and weight, whereas irrigation at ≤70% reduced kernel yield, except for SDI_70_ in the third season. Water deficit also accelerated hull split.
García-Tejero et al., 2018b [87] and 2019 [77] Spain	*Three treatments:* (i) FI. (ii) sRDI_50, III_. (iii) LFDI defined by threshold value of Ψleaf (−2 Mpa) during the kernel-filling stage.	‘Guara’ grafted GF-677 *5-young*	1	6 × 7	CWSI would be the most appropriate thermal index to monitor the almond water status especially when thermal readings are taken between 11:30 and 17:30 h, these coinciding with daily period of maximum evapotranspirative demand. No differences in yield between FI and LFDI, leading to important water savings (27 and 40%).
López-López et al., 2018a [100] Spain	*Four treatments:* (i) FI. (ii) mSDI_75_. (iii) sRDI_40-III_. (iv) ssRDI_15-III_.	‘Guara’ grafted GF-677 *5 to 7*	3	6 × 7	Maximum average yield of 2508.4 kg ha^−1^ were obtained from control trees, while the three deficit irrigation strategies yielded 2147.5, 2038.2, and 1496.9 kg ha^−1^ Although values varied from year to year, the WP averaged 0.23 kg/m^3^ for the 3 years and did not differ among treatments.
Prgomet et al., 2020 [142] Portugal	*Four treatments:* (i) FI. (ii) SDI_70_. (iii) SDI_35-III_. (iv) Rainfed.	‘Ferragnes’ grafted GF-677 *Young*	2	6 × 4	RDI_35_ during kernel filling caused no yield loss while maintaining acceptable physiological status. Under rainfed or severe water restriction, trees undergo a series of phenological, morphological, physiological, and biochemical changes.
García-Tejero et al., 2020 [97] Spain	*Three treatments:* (i) FI. (ii) SDI_75_. (iii) SDI_65_.	‘Guara’ ‘Lauranne’ ‘Marta’ grafted GN15 *7*	1	8 × 6	Moderate SDI_65–75_ can improve almond quality traits without severely compromising production.
Gutierrez-Gordillo et al., 2019a [114] Spain	*Three treatments:* (i) FI. (ii) SDI_150 over-irrigation_. (iii) RDI_65-III_.	‘Guara’ ‘Lauranne’ ‘Marta’ grafted GN15 *10-Adult*	2	8 × 6	‘Marta’ responded positively to over-irrigation, suggesting greater sensitivity to drought, while ‘Guara’ and ‘Lauranne’ maintained yields under RDI_65_ during the kernel-filling stage. with water savings of approximately 3000 m^3^ ha^−1^. Yield and physiological responses differed significantly among cultivars.
Gutierrez-Gordillo et al., 2019b [115] Spain	*Two treatments:* (i) FI. (ii) mSDI_75_. *With biostimulants and traditional fertilization only.*	‘Guara’ ‘Lauranne’ ‘Marta’ grafted GN15 *5-Young*	1	8 × 6	Mild SDI_75_ reduced kernel yield by 14% in ‘Guara’ and 13% in ‘Marta’, whereas yield was unaffected in ‘Lauranne’. Biostimulant application increased yield by 15–20% in ‘Guara’ and eliminated the yield reduction under SDI_75_ in ‘Marta’; irrigation water productivity also increased under SDI_75_ with biostimulant application.
Gutierrez-Gordillo et al., 2020 [116] Spain	*Three treatments:* (i) FI. (ii) mSDI_75_. (iii) SDI_65_.	‘Guara’ ‘Lauranne’ ‘Marta’ grafted GN15 *6*	2	8 × 6	‘Guara’ showed a sharp yield decline under SDI_65_; ‘Lauranne’ and ‘Marta’ maintained yields under SDI. In addition ‘Marta’ showed the highest antioxidant activity, ‘Guara’ was the richest in organic acids, and ‘Lauranne’ had the highest fatty acid content. SDI strategies improved almond quality parameters related to their nutritional and sensory composition, with significant water savings (reductions of 25–35%) and without important yield loss.
Gutierrez-Gordillo et al., 2020b [143] Spain	*Three treatments:* (i) FI. (ii) mSDI_75_. (iii) SDI_65_.	‘Guara’ ‘Lauranne’ ‘Marta’ grafted GN15 6–7	2	8 × 6	Crop water monitoring was done by measurements of Tc, Ψleaf, and gs. Ψleaf was the most responsive parameter to reflect differences in almond water status.
Lipan et al., 2019a [22] Lipan et al., 2019b [144] Spain	*Four treatments* *using the stress integral (SI):* (i) FI. (ii) mRDI **_<_**_−1.5 MPa III_. (iii) sRDI _SWP <−2 MPa III_. (iv) SDI *in a constant daily rate around 1–2 mm per day, with a maximum water applied of 100 mm.*	‘Vairo’ Rootstock not reported 7	1	6 × 8	Short-term RDI and SDI had little effect on yield or on nutritional quality, including sugars, organic acids, phenolics, volatile compounds, and antioxidant content. Overall, irrigation strategies did not markedly affect almond fruit quality; however, moderate RDI can improve certain quality attributes of the nuts.
Lipan et al., 2020a [145] Spain	*Four treatments* (*similar to previous article*).	‘Vairo’ Rootstock not reported *7 to 9*	3	6 × 8	Long-term RDI can enhance quality, particularly dry matter, color, K, Fe, Zn, sugars, organic acids, and antioxidant activity.
Lipan et al., 2020b [117] Spain	Three *treatments*: (i) FI. (ii) SDI _150_. (iii) RDI_65-III_.	‘Marta’ ‘Guara’ ‘Lauranne’ grafted GF-677 *11*	1	6 × 8	Marta’ was more sensitive to deficit irrigation and showed the greatest improvements under RDI65, whereas ‘Guara’ and ‘Lauranne’ were more tolerant of water deficit.
Razouk et al., 2021 [146] Morocco	*Three treatments:* (i) FI. (ii) RDI_75-II and III_. (iii) RDI_50 II and III_.	‘Tuono’ Rootstock not reported *7–9*	3	5 × 3	RDI_-50_ maintained almond yield and fruit size, while increasing kernel soluble sugars, polyphenol content and oil concentration; irrigation water use was reduced by 50%. However, RDI_-50_ increased the depth of almond kernel epidermal grooves, potentially reducing commercial value.
Gutiérrez-Gordillo et al., 2021 [119] Spain	*Three treatments* (i) FI. (ii) RDI_70–40-III_ UAV-based CWSI and NDVI.	‘Guara’ ‘Marta’ ‘Lauranne’ ‘Marcona’ grafted GN15 *5*	1	6 × 8	CWSI detected moderate to severe RDI stress but failed to detect mild stress, whereas Ψstem was more sensitive under mild stress. NDVI was less sensitive than CWSI, and no cultivar-specific differences were detected. Thus, UAV-based CWSI is suitable for monitoring water status, while Ψstem is preferred for early stress detection.
Vivaldi et al., 2021 [147] Italy	*Four treatments:* (i) FI_130_ over irrigated with desalinated reclaimed water, ECw ≈ 1 dS m^−1^. (ii) FI with saline reclaimed water (RW), ECw ≈ 3 dS m^−1^. (iii) RDI_80-III_ with desalinated reclaimed water, ECw ≈ 1 dS m^−1^. (iv) RD_80-III_ with saline reclaimed water ECw ≈ 3 dS m^−1^.	‘Guara’ grafted Rootpac^®^ 20 *3–6*	3	Potted trees	Water defict and saline reclaimed water (RW) reduced plant water status and gs, while their combination caused a greater reduction in An. RW combined with RDI was therefore unsuitable for almond irrigation.
García-Tejero et al. 2018 [148] Italy	*Three treatments:* (i) FI. (ii) m RDI_75-III_. (iii) s RDI_50-III_.	‘Guara’ grafted GF-677 7	1	6 × 6.5	Thermal indices, particularly CWSI, effectively discriminated irrigation treatments and correlated with almond water status, supporting their use for monitoring RDI-induced water stress and irrigation scheduling.
Moldero et al., 2021b [149] Spain	*Four treatments:* (i) FI. (ii) moSDI_65_. (iii) moRDI_65-III_. (iv) sRDI_35-II- III and IV_.	‘Guara’ grafted GF-677 *Adult*	6	6 × 8	Moderate RDI_65_ and SDI_65_ produced similar yields, whereas severe RDI_35_ caused the greatest yield loss. No decline in DI performance was detected over the six-year period, indicating sustained long-term productivity under moderate deficit irrigation.
González-Zamora et al., 2021 [150] Spain	*Two treatments:* (i) FI. (ii) mRDI_70_.	‘Guara’ ‘Vairo’ Rootstock not reported *7–10*	4	6 × 8	Restricted water use such as RDI_70_ can help reduce the foliar damage of important pests (*T. urticae*) and the abundance of other secondary pests in almond orchards.
Moldero et al., 2022 [151] Spain	*Three treatments:* (i) FI. (ii) ssSDI_25_. (iii) Rainfed, *imposed in the first year*.	‘Guara’ grafted GF-677 *Adult*	3	7 × 6	Rainfed and very severe SDI_25_ caused lasting yield losses, with rainfed killing 92% of trees. In the two following years, the negative impact on yields persisted due to reductions in fruit load (carry-over effects) even though trees in DI and RF were restored to full-irrigation levels.
Jofre-Čekalović et al., 2022 [36] Spain	*Four treatments:* (i) FI; (ii) Mild to severe *SDI_100–25_*. (iii) Moderate SDI_100–75_. (iv) Mild to moderate SDI_100–40_. TSEBS2 + S3 mode.	‘Vairo’ grafted GF-677 *Adult*	4	6 × 6	TSEBS2 + S3 estimated almond actual evapotranspiration (ETa) with an RMSD of 0.6–2.5 mm day^−1^ against the soil-water-balance model. The most water-limited treatment showed 13–20% lower cumulative ETa than the fully irrigated treatment, confirming the model’s ability to discriminate water-use differences among irrigation regimes.
Drechsler and Kisekka, 2022b [152] California	*Three treatments:* (i) FI. (ii) mo RDI_75-III_. (iii) severe RDI_50-III_.	‘Nonpareil’ ‘Aldrich’ ‘Butte’ grafted Peach *Mature*	4	4.3 × 6	Variety-specific RDI improved selected physical quality traits in ‘Aldrich’ and ‘Butte’ cultivars, but did not significantly increase marketable kernel yield after two years.
Conti et al., 2023 [153] Italy	*Three treatments:* (i) FI. (ii) mRDI_80-III_. (iii) msRDI_60-III_.	‘Guara’ Rootstock not reported *7*	1	5 × 5	Mild RDI_80_ and moderate to severe RDI_60_ reduced irrigation water by 85% and 75%, respectively, while maintaining yield at approximately 1.2 t ha^−1^. msRDI_60_ reduced An and gs more strongly and increased the incidence of adherent hulls.
Barreales et al., 2023 [18] Portugal	*Two treatments:* (i) FI. (ii) sRDI**_35_**_-__III_. *Both combined with foliar kaolin application*	‘Constantì’ ‘Vairo’ grafted GF-677 *Adult*	3	7 × 5	Severe RDI_35_ during kernel filling stage did not reduce yield in ‘Constantí’, but reduced it in ‘Vairo’. The combined use of RDI and foliar kaolin enhanced WUE and supported sustainable almond production.
Gutierrez-Gordillo et al., 2023 [154] Spain	*Three treatments:* (i) FI. (ii) mSDI**_75_**. (iii) moSDI_65_.	‘Guara’ ‘Marta’ ‘Lauranne’ grafted GN15 *Adult*	1	6 × 8	No significant irrigation-treatment or cultivar effects were detected in An, Ψleaf or yield. Triose phosphate utilization (TPU) limitation increased during kernel filling and postharvest, accompanied by reduced maximum electron transport rate (Jmax) and maximum Rubisco carboxylation capacity (Vcmax).
Sperling et al., 2023 [120] Israel	*Three treatments:* (i) FI. (ii) Deficit irrigation (def). (iii) Adaptive irrigation (adp) according to the physiological responses of almond trees to water availability.	‘UmElFahm’ Rootstock not reported *Adult*	2	6 × 8	Deficit irrigation reduced Ψstem water potential below −2 MPa and gs to 0.15 mol m^−2^ s^−1^, limiting An to ~14 µmol m^−2^ s^−1^ and reducing yield by 35% by the second season. An irrigation level of ~960 mm supported high yields
Quintanilla-Albornoz et al., 2024 [39] Spain	*Three treatments:* (i) FI, (ii) Mild stress. (iii) Severe stress. Four production systems: open vase with severe pruning with 6 × 6 m; minimal pruning (MP) with 5 × 3.5; central axis with 5 × 3; hedgerow with 4.5 × 3.	‘Marinada’ grafted INRA GF-677 *12–13*	2	Open vase 6 × 6 5.5 × 3.5 5 × 3 4 × 3	Severe water stress reduced kernel yield and nut load by 31.6% and 34.5%, respectively. Among the production systems, the hedgerow system maintained comparable kernel yields while exhibiting lower transpiration and achieved the highest water productivity (0.43 kg m^−3^), followed by the Central Axis (0.36 kg m^−3^), Open Vase-MP (0.34 kg m^−3^), and Open Vase (0.33 kg m^−3^) systems.
Calderón-Pavón et al., 2025 [155] Spain	*Two treatments:* (i) FI. (ii) mRDI_80-II and IV, and 40-III_. Under both organic (OPS) and conventional (CPS) production systems in both studied period corresponding to 3 and 7 years of trees.	‘Marcona’ grafted GN-15 *3 young* vs. *7 Adult*	2	7 × 6	DI reduced yield, particularly in young trees. CPS consistently achieved higher yields than OPS. Improvements in TPC, AA, OA and PUFAs were observed, although the magnitude of these responses varied with the production system and tree age.
Orozco et al., 2025 [156] Spain	*Four treatments:* (i) FI. (ii) SDI_122_ (moderate over irrigation). (iii) SDI_144_ (high over- Irrigation). (iv) SDI_70_.	‘Lauranne’ Rootstock not reported *3 to 6 young*	4	7 × 4	Over-irrigation did not increase yields; 30% deficit cut ~20% over four years, showing almonds’ sensitivity to prolonged stress. Irrigation scheduling based on water balance and remote sensing improved water management, allowing precise irrigation control while maintaining almond orchard performance and increasing water-use efficiency.
Bellvert et al., 2026 [157] Spain	*Two treatments:* (i) Rainfed. (ii) Control (limited irrigation, ~1100 m^3^/ha (May–Nov) *Kaolin Spray.*	‘Vairo’ grafted GF-677 *Adult*	2	6 × 6	Rainfed (~22% water) lost 92% yield and had 42% mortality; control and limited irrigation sustained moderate yields; kaolin had little effect.
Gispert et al., 2026 [158] Spain	*Two treatments:* (i) RDI_40-III_. (ii) Rainfed.	‘Ferragnès’ ‘Masbovera’ ‘Glorieta’ ‘Francolí’ ‘Guara’ ‘Lauranne’ grafted GF-677 *Adult*	1	Open vase	Water stress tolerance varied among almond cultivars, with drought-resilient genotypes maintaining better physiological performance, water status, and stress adaptation under limited water conditions. ‘Guara’ and ‘Masbovera’ were the most resistant, ‘Glorieta’ and ‘Francolí’ were intermediate, and ‘Lauranne’ and ‘Ferragnès’ were the least resistant.

**Table 3:** Yield and water-use responses to deficit irrigation in almond.

Strategy	Cultivar/Age	Deficit	Yield vs. FI	Reported Water Saving	Key Finding	Evidence Strength
SDI	Ruby, mature	SDI_50–125_	Increased to SDI125; then plateaued	NR	Yield response was nonlinear, with no additional yield benefit above SDI_125_.	Moderate [109]
SDI	Nonpareil, mature	SDI_85–55_	~5–30% reduction	~15–45%	Increasing deficit reduced yield, mainly through smaller kernels.	Well-supported [70]
SDI	Ferragnès, mature	SDI_70–50_	Reduced; ~23% at SDI50	30–50%	Severe deficit reduced vegetative growth and yield.	Well-supported [57,136]
SDI	Marta, young	SDI_80–60_	Minor to significant reduction	20–40%	Increasing deficit reduced yield and vegetative growth.	Moderate [126]
SDI	Guara, mature	SDI_65–25_	Maintained at SDI65; substantial loss at SDI25	~35–75%	Moderate deficit maintained yield, whereas severe stress reduced productivity and kernel quality.	Moderate [148,150]
SDI	Vairo, mature	Rainfed/extreme drought	~92% reduction	Near-total	Extreme drought caused major yield loss and tree mortality.	Well-supported; limited comparability [155]
RDI	Ferragnès, mature	Stage III	~31% reduction	~50%	Severe Stage III deficit reduced yield despite substantial water saving.	Moderate [136]
RDI	Ferragnès/Guara, mature–young	Moderate, kernel filling	Maintained or slightly reduced	NR	Appropriately timed moderate deficits can maintain productivity.	Moderate [76,141]
RDI	Nonpareil, mature	~85–70% FI	Little/no reduction to reduced	~15–30%+	Yield response increased with deficit severity and varied among seasons.	Well-supported [70,140]
PRD	Marta, mature	PRD_70–30_	Minor to greater reduction	~30–70%	Increasing deficit reduced yield and growth, while water productivity increased.	Moderate [108,129]

**Note:** For SDI_85–55_, reported values represent nominal irrigation reductions relative to FI and should not necessarily be interpreted as measured irrigation-water savings. Water-saving values are study-specific reported ranges and are not statistically pooled estimates. NR, not reported; FI, full irrigation; PRD, partial root-zone drying; RDI, regulated deficit irrigation; SDI, sustained deficit irrigation; WP, water productivity.

**Table 4:** Overall evidence synthesis of deficit irrigation strategies in almond.

Strategy	Reported Water-Saving Range Across Cited Studies	Yield Response Under Moderate Deficit	Yield Response Under Severe Deficit	Long-Term Evidence	Evidence Strength	Main Uncertainty/Qualification
SDI	Approximately 20–63% across cited studies; substantially greater under extreme drought/rainfed conditions	Yield can remain relatively close to FI in some cultivars, particularly under moderate deficits	Yield, kernel size and vegetative growth can decline substantially as deficit severity increases	Moderate to strong	Well-supported	Magnitude of yield response is strongly cultivar-, tree-age- and environment-dependent; no universal SDI threshold can be established
RDI	Approximately 27–50% in several cited Stage III treatments	Yield can remain close to FI when deficits are appropriately timed and moderate in severity	Severe or prolonged deficits can cause substantial yield and kernel-weight reductions	Moderate to strong, depending on cultivar and timing	Well-supported	Water-saving potential and yield penalty depend strongly on deficit timing, severity and cultivar; the optimum threshold remains unresolved
PRD	Approximately 30–70% across cited treatments	Moderate PRD can maintain relatively high productivity in some cultivars	Severe PRD can reduce kernel weight, vegetative growth and yield	Limited to moderate	Moderate	Evidence is insufficient to establish a consistent yield or agronomic advantage of PRD over RDI
Rainfed/extreme drought	Very high or near-total reduction in irrigation input; not equivalent to managed deficit irrigation					

**Table 5:** Evidence strength and unresolved conclusions regarding deficit irrigation in almond.

Topic	Synthesized Conclusion	Evidence Strength	Contested/Unresolved Aspect
Deficit severity	Increasing SDI, RDI, or PRD severity generally increases the risk of reductions in yield, kernel weight, and vegetative growth.	Well-supported	The magnitude of the response varies among cultivars, tree ages, environments, and seasons.
Moderate SDI	Moderate SDI can maintain relatively high yield in some cultivars, particularly when water deficits are not severe or prolonged.	Moderate	A universal SDI threshold for maintaining yield cannot be established across cultivars.
Deficit timing	Yield response depends strongly on the phenological stage at which water deficit is imposed.	Well-supported	The quantitative sensitivity thresholds for individual stages remain incompletely defined.
Stage I sensitivity	Severe early-season water stress can impair reproductive and vegetative development.	Moderate	Insufficient evidence exists to define a universal Stage I yield-penalty threshold or optimal RDI level.
Stage II response	Severe water stress during Stage II can substantially reduce yield, whereas moderate deficits may have smaller effects.	Moderate	The yield response to moderate Stage II RDI remains unresolved across cultivars and environments.
Stage III RDI	Moderate, appropriately timed Stage III RDI can substantially reduce irrigation inputs while maintaining yield close to FI in several cultivars.	Well-supported	The magnitude of achievable water saving and the associated yield penalty remain cultivar- and environment-dependent.
Severe Stage III deficit	Severe or prolonged Stage III deficits increase the risk of reductions in yield, kernel weight, and vegetative performance.	Well-supported	The deficit threshold beyond which yield losses become substantial remains unresolved.
Postharvest RDI	Moderate postharvest RDI can maintain current-season productivity in several cultivars while reducing irrigation inputs.	Moderate	The magnitude of effects on subsequent-season flowering, fruit set, and yield varies among cultivars and remains unresolved quantitatively.
RDI vs. SDI	Available evidence suggests that deficit severity and timing may have greater effects on yield than the nominal irrigation strategy itself.”	Moderate	Direct comparisons are limited, and the relative response can vary with cultivar and season.
PRD vs. RDI	PRD can reduce water use and may improve water productivity under some conditions.	Moderate	A consistent yield or agronomic advantage of PRD over RDI remains unresolved.
Guara tolerance	SDI65 maintained yield within approximately 0–5% of FI over six years.	Moderate	This provides strong longitudinal evidence for ‘Guara’ but should not be interpreted as a universal threshold for other cultivars.
Water productivity vs. yield	Water productivity can increase even when yield declines under severe deficit.	Well-supported	High water productivity should not be interpreted as evidence of yield preservation or overall production sustainability.
Long-term deficit effects	Repeated or prolonged severe deficits can reduce tree growth, kernel development, and subsequent productivity.	Moderate	Quantitative evidence for cumulative multi-year effects remains limited across cultivars and environments.
Physiological acclimation	Repeated moderate water stress can induce physiological adjustments that may improve water-use efficiency.	Moderate	Whether physiological acclimation consistently prevents long-term yield losses remains unresolved.
Yield–water trade-off	Greater water saving can eventually be accompanied by substantial yield loss when deficit severity exceeds crop tolerance.	Well-supported	The economically or agronomically optimal balance between water saving and yield preservation remains unresolved.

**Note:** Evidence strength was classified as well-supported, moderate, unresolved, or insufficient evidence according to the consistency, quantity, duration, and comparability of the available studies. “Unresolved” indicates that available studies provide conflicting or strongly context-dependent results, whereas “insufficient evidence” indicates that too few comparable studies are available to support a reliable conclusion.

**Table 6:** Comparative summary of irrigation strategies for almond under water deficit.

Irrigation Approach	Reported Water-Saving Potential	Yield Response Commonly Reported	Main Advantage	Main Limitation/Uncertainty	Evidence Strength
FI	Low or none relative to FI	Generally highest yield under non-limiting water supply	Minimizes water stress and supports high productivity	Requires relatively high irrigation inputs and may not be feasible under water scarcity	Well-supported
Moderate SDI	Approximately 20–63% in the cited studies	Yield can remain relatively high in some cultivars, but moderate reductions may occur	Simple and consistent seasonal deficit; can improve water productivity	Response is strongly cultivar- and environment-dependent; cumulative stress may occur	Moderate
Severe SDI	Generally high in the cited studies	Yield, kernel size, and vegetative growth can decline substantially	Provides substantial irrigation-water reduction under severe water limitation	Greater risk of yield loss, kernel-quality effects, and cumulative tree stress	Well-supported
Moderate RDI	Approximately 27–50% in several cited treatments	Yield can remain close to FI when deficits are appropriately timed	Allows water stress to be targeted to relatively less-sensitive developmental periods	Requires accurate timing and management; optimum deficit level varies among cultivars	Well-supported
Severe RDI	High in the cited treatments	Yield response is variable; substantial losses can occur when deficits are severe or poorly timed	Can achieve large water savings under severe water limitation	Greater risk of yield, kernel-weight, vegetative-growth, and subsequent-season losses	Well-supported
Moderate PRD	Approximately 30–70% in the cited treatments	Relatively high productivity can be maintained under moderate restriction in some cultivars	May improve water productivity and alter plant water relations	Evidence is more limited than for RDI/SDI, and benefits over RDI are inconsistent	Moderate
Severe PRD	High in the cited treatments	Kernel weight, tree growth, and yield may decline as water restriction increases	High water productivity can occur under strong water limitation	Yield and vegetative-growth penalties increase with deficit severity; comparative advantage over RDI remains unresolved	Moderate
Rainfed/extreme drouht	Very high or near-total reduction in irrigation input	Very large yield losses and, under prolonged drought, tree mortality may occur	Eliminates irrigation input	Uncontrolled water stress makes it unsuitable as a general managed deficit-irrigation strategy	Well-supported

**Note:** Reported water-saving values represent ranges observed in the cited studies and are not statistically pooled estimates. Where treatments were defined as a percentage of full irrigation, the percentage represents a nominal reduction in irrigation input unless actual water use was measured. FI, full irrigation; PRD, partial root-zone drying; RDI, regulated deficit irrigation; SDI, sustained deficit irrigation.

## Data Availability

Data are contained within the article.

## Abbreviations

Deficit irrigation strategies: DI = deficit irrigation; RDI = regulated deficit irrigation; SDI = sustained deficit irrigation; PRD = partial root-zone drying; LFDI = low-frequency deficit irrigation. Evapotranspiration: ETo = reference evapotranspiration; ETc = crop evapotranspiration; ETa = actual evapotranspiration; Kc = crop coefficient. Defined levels of water-deficit treatments: FI = full irrigation (100% ETc); OI = over-irrigation; CTRL = control; RF = rainfed; FRM = farmer-managed treatment. Water-stress categories were defined according to the proportion of crop evapotranspiration (ETc) supplied: mm = very mild stress (≈80% ETc); m = mild stress (≈70% ETc); mo = moderate stress (≈60% ETc); ms = moderate-to-severe stress (≈50% ETc); s = severe stress (≈40% ETc); and ss = very severe stress (≈20–25% ETc). Fruit growth stages and deficit irrigation application: Stage I = early season (flowering to fruit set and rapid fruit growth); Stage II = mid-season (kernel expansion and pit hardening); Stage III = kernel filling and preharvest; Stage IV = maturity and harvest, including hull split and the postharvest period. Subscripted Roman numerals and Arabic numerals indicate the phenological stage and irrigation level, respectively. Both the percentage of full irrigation and the phenological stage (I, II, III, or IV) are indicated as subscripts. For example, RDI_70_–_III_ indicates 70% of full irrigation applied during Stage III. When cut-off is applied during a specific phenological stage, cut-off is indicated as a subscript alongside the specific phenological stage (e.g., RDI_III_-cut-off). When PRD is applied during a specific phenological stage, the stage is indicated as a subscript alongside the irrigation level (e.g., PRD_70_–_III_ = 70% of full irrigation applied during Stage III). When PRD is applied continuously throughout the irrigation season, it is denoted as PRD_70_–_SDI_. Over-irrigation treatments are similarly indicated using subscripts (e.g., SDI_125_ = 125% of full irrigation). Morphological, physiological, and chemical indicators of water stress: TDV = trunk diameter variation; MDS = maximum daily trunk shrinkage; TGR = daily trunk growth rate; SI_m_DS = MDSactual/MDSreference (normalized form of MDS intensity signal); SI_MDS_ = daily trunk shrinkage signal intensity; SPAC = soil–plant–atmosphere continuum; PWS = plant water stress; WP = water productivity; IWP = irrigation water productivity; IWU = irrigation water use; WUEI = Yield/Irrigation water applied (kg m^−3^); WPET = water productivity based on evapotranspiration; WUE = water use efficiency; WPT = transpiration efficiency; RWC = relative water content; WPF = water production function; VWC = volumetric water content; Ψs = soil water potential; Ψatm = atmosphere water potential; Ψr = root water potential; Ψx = xylematic water potential; Ψleaf = leaf water potential; Ψstem = stem water potential; Ψms = midday stem water potential; Ψml = midday leaf water potential; Ψpd = predawn leaf water potential; Rs = soil resistance; Rr = radical resistance; Rst = stems resistance; Rl = leaf resistance; E = external environment; g_s_ = stomatal conductance; A_n_ = net CO_2_ assimilation rate; Tr = transpiration rate; Tl_eaf_ = leaf temperature; Tc = canopy temperature; Fᵥ = variable fluorescence; F_m_ = maximum fluorescence; Fᵥ/F_m_ = maximum quantum efficiency of photosystem II (PSII), as determined by chlorophyll fluorescence; TPU = triose phosphate utilization; Jmax = maximum electron transport rate; Vcmax = maximum Rubisco carboxylation capacity; CWSI = crop water stress index; SF = sap flow; MDTC = maximum daily transport capacity; DCFA = discounted cash-flow analysis; RMSD = Root Mean Square Deviation. Kernel composition and nutritional quality: TPC = total phenolic content; AA = antioxidant activity; SC = sugar content; OA = organic acids; USFA = unsaturated fatty acids; SFA = saturated fatty acids; MUFA = monounsaturated fatty acids; PUFA = polyunsaturated fatty acids; PUFA/SFA = ratio of polyunsaturated to saturated fatty acids; N = hardness, expressed in newtons. Remote-sensing tools and technologies: RS = remote sensing; UAV = unmanned aerial vehicle (drone); MAVs = manned aerial vehicles; AI = artificial intelligence; Cloud = internet-based computing and data-storage systems; CSA = Climate-smart agriculture; S1-SWC = Sentinel-1 C-band radar-derived soil water content; Vis = vegetation indices; CCCI = Canopy Chlorophyll Content Index; MTCI = MERIS Terrestrial Chlorophyll Index; CRI2 = Carotenoid Reflectance Index 2; SAVI = Soil-Adjusted Vegetation Index; FRF = Far-red fluorescence; fAPAR = Fraction of Absorbed Photosynthetically Active Radiation; TSEB = two-source energy balance model; TSEB–2TR = two-source energy balance model integrated with thermal remote sensing; TSEBS2 + S3 = remote-sensing approach based on sharpened Sentinel-2 and Sentinel-3 imagery; NDII = Normalized Difference Infrared Index; NDVI = Normalized Difference Vegetation Index; LiDAR = Light Detection and Ranging; NDWI = Normalized Difference Water Index; ΔNDVI = change in the Normalized Difference Vegetation Index; HYDRUS-1D-SWC = soil water content simulated using the one-dimensional HYDRUS-1D model; HYDRUS-2D/3D = two- and three-dimensional HYDRUS models for spatial simulation of water movement in soil; IoT = Internet of Things; WSNs = wireless sensor networks; ITC = increment in canopy temperature; NWSB = non-water-stress baselines; WSB = water-stress baselines; LVDTs = linear variable displacement transducers; RMSE = root mean square error; SCOPE = soil canopy observation of photosynthesis and energy fluxes.
